# Fe^II^-catalysed insertion reaction of α-diazocarbonyls into X–H bonds (X = Si, S, N, and O) in dimethyl carbonate as a suitable solvent alternative†

**DOI:** 10.1039/c9ra07203a

**Published:** 2019-10-02

**Authors:** Nour Tanbouza, Hoda Keipour, Thierry Ollevier

**Affiliations:** Département de Chimie, Université Laval 1045 Avenue de la Médecine Québec QC G1V 0A6 Canada

## Abstract

The insertion reaction of a broad range of diazo compounds into Si–H bonds was found to be efficiently catalysed by Fe(OTf)_2_ in an emerging green solvent *i.e.* dimethyl carbonate (DMC). The α-silylated products were obtained in good to excellent yields (up to 95%). Kinetic studies showed that the extrusion of N_2_ to form an iron carbene intermediate is rate-limiting. The iron-catalysed insertion reaction of methyl α-phenyl-α-diazoacetate into polar X–H bonds (S–H, N–H, and O–H) was also established in DMC.

## Abstract 2

The insertion reaction of a broad range of diazo compounds into Si–H bonds was found to be efficiently catalysed by Fe(OTf)_2_ in an emerging green solvent *i.e.* dimethyl carbonate (DMC).

## Graphical abstract

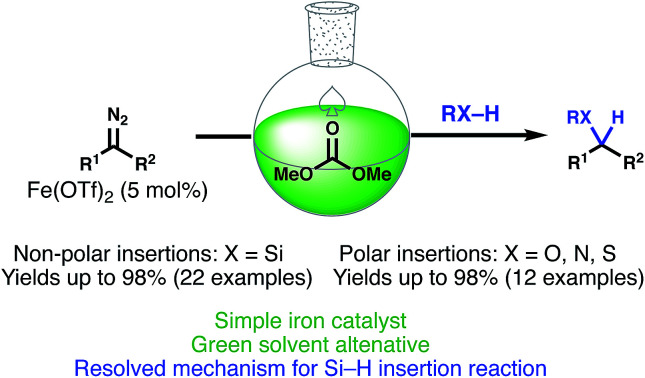

Dimethyl carbonate (DMC), the simplest among the dialkyl carbonate family (DACs), is an emerging and interesting green reagent and solvent alternative in organic synthesis due to its high biodegradability and low toxicity.^1,2^ It is exploited industrially for its efficiency as a solvent for numerous applications,^3^ however it still remains underused as a solvent in homogeneous catalysis. Even though major contributions have been achieved to promote the twelve principles of green chemistry,^4^ searching for efficient green solvent alternatives still remains a challenge for chemists.^5^ Solvent selection guides are huge influencers for rendering the choice of an appropriate solvent less tedious in terms of life cycle evaluation.^6^

For this study, we were successful in establishing dimethyl carbonate as an appropriate solvent for an iron-catalysed insertion reaction of acceptor/donor diazo compounds into Si–H bonds and polar X–H bonds (X = S, N, and O). Diazo compounds are the most commonly used carbene precursors. They can be de-diazonized to obtain highly reactive free carbene intermediates or metal carbene species. Then, varieties of chemical transformations can occur, such as X–H (X = C, S, N, O, and Si) insertions,^7^ cyclopropanation,^7*b*^ ylide formation,^8^ and Wolff rearrangement.^9,10^ Notably, the transition metal-catalysed insertion reaction of diazo compounds into various X–H bonds represents a facile route towards different tailored functionalized compounds which are arduous to obtain through other routes.^11^

Special attention has been paid to the insertion reaction of diazo compounds into Si–H bonds, because even though silicon is vastly abundant on earth, routes towards organosilicons are not. Organosilicon compounds are increasingly mainstream within a variety of applications.^12^ They are pertinent in the pharmaceutical sector where the integration of silicon motifs as carbon isosteres has allowed distinctive therapeutic potential in a number of biologically active molecules.^13^ The transition metal-catalysed insertion reaction into Si–H bonds is a facile and atom-economic method to produce organosilicons.^14^ This reaction was first described by Kramer and Wright in 1963.^15^ Since then, pivotal breakthroughs have been made with Ru-,^16^ Rh-,^17^ Ir-,^18^ Ag-,^19^ Cu-,^20^ and enzyme-catalysed^21^ insertion reactions of diazo compounds into the Si–H bond. Iron catalysis being one of our group's major areas of research,^22^ we developed a highly efficient iron-catalysed insertion reaction of α-diazoesters into Si–H bonds.^23^ Afterwards, an enantioselective iron-catalysed version was developed by Xie and Lin.^24^ The use of iron salts in transition metal catalysis is gaining traction due to their low toxicity, abundance, and environmental benignity. Iron catalysts have proved on and on to be efficient and green substitutes of conventional noble metal catalysts.^25^

Even though iron-catalysed Si–H insertion reactions were successfully developed to render it more sustainable, they were still conducted in dichloromethane (CH_2_Cl_2_), a solvent which is commonly used in homogeneous catalysis due to its good solubility of most organics, low polarity, and non-coordinating properties. However, it is notorious for its toxicity, harmfulness to both human health and the environment, and costly disposal.^26^ Herein, we disclose the use of DMC as an appropriate solvent alternative to CH_2_Cl_2_ in a ferrous system for the insertion reaction of α-diazocarbonyl compounds into Si–H bonds. Kinetic studies are then employed to establish the rate determining step of an iron-catalysed insertion of α-diazoesters into Si–H bonds. This study is then further extended to cover insertion reactions of α-diazoesters into different polar X–H bonds (X = O, N, and S).

A series of Fe^II^ and Fe^III^ salts were selected for screening in DMC with Et_3_SiH (Table 1).^27^ The following screening shows that the nature of the catalyst greatly influences the reactivity of the diazo compound. Fe(OTf)_2_ was capable of affording the α-silylated product 2a in a 95% yield within 6 h (entry 1).^28^ FeBr_2_ and FeCl_2_ led to poor conversions after 72 h (entries 2 and 3). The diazo compound 1a was unreactive towards Fe(OAc)_2_ and was recovered quantitatively (entry 4). Fe(BF_4_)_2_·6H_2_O resulted in a low 6% yield which was due to insertion with H_2_O and dimerization (entry 5). Interestingly, both Fe(OTf)_3_ and Fe(acac)_3_ led to the formation of the silyl enolate, which was attributed to the strong Lewis acidity of Fe^III^ salts (entries 6 and 7).

**Table 1:** Screening of different metal salts for the insertion of methyl α-phenyl-α-diazoacetate into Si–H bonds^a^

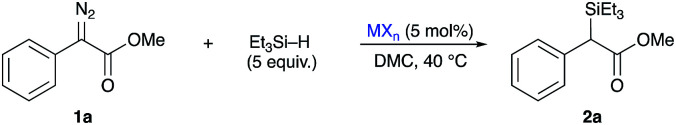			
Entry	MX_*n*_	Time (h)	Yield (%)
1	Fe(OTf)_2_	6	95
2	FeBr_2_	72	4^b^
3	FeCl_2_	72	45^b^
4	Fe(OAc)_2_	48	0^c^
5	Fe(BF_4_)_2_·6H_2_O	18	6^d^
6	Fe(OTf)_3_	48	—e
7	Fe(acac)_3_	48	—e

a 1a (0.25 mmol), catalyst (5 mol%), Et_3_SiH (1.25 mmol), DMC (2 ml).

b Incomplete conversion.

c No conversion and recovery of α-diazoester 1a.

d Insertion with H_2_O and dimerization as major pathways.

e A mixture of *O*-silyl enolate and dimerization product was obtained.

In order to conduct an appropriate screening of solvents, and to figure out the correlation between the solvent's solvatochromic properties and how it affects the course of this reaction, a Kamlet–Taft plot of aprotic solvents was used to this advantage (Fig. 1).^29^ The plot includes different common organic solvents previously screened for this reaction in addition to a screening of different classical and less common green solvent alternatives (see ESI† for individual yields and reaction times). Solvents that produced very good yields of the insertion product (>90%) are coloured green (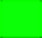), whilst those leading to moderate yields (between 90% and 50%) and poor yields (<50%) are identified in yellow (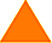) and red (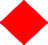), respectively. As it can be seen from the distribution of solvents on the Kamlet–Taft plot, solvents with high basicity (*β* > 0.4) and low polarizability (π* < 0.5) resulted in moderate and low yields of the desired insertion product. It can be deduced that the reaction prefers a reaction media that is low in polarity and basicity which is delivered by all of CH_2_Cl_2_, dichloroethane (DCE, (CH_2_Cl)_2_), benzene (PhH), chlorobenzene (PhCl), bromobenzene (PhBr), and DMC. An exception is observed with toluene (PhMe) which is reactive towards metal carbenes. Another is with MeCN; this can be attributed to the low solubility of the silane. Following the solvent selection guides, DMC was chosen for this reaction. Other organic carbonates, *i.e.* diethyl carbonate (DEC) and propylene carbonate (PC), were not as efficient as dimethyl carbonate. Polar protic solvents, H_2_O and EtOH, were also tested in this reaction where no sign of the insertion product was observed, and only O–H insertion products were obtained in high yields (see ESI†).

**Fig. 1:**
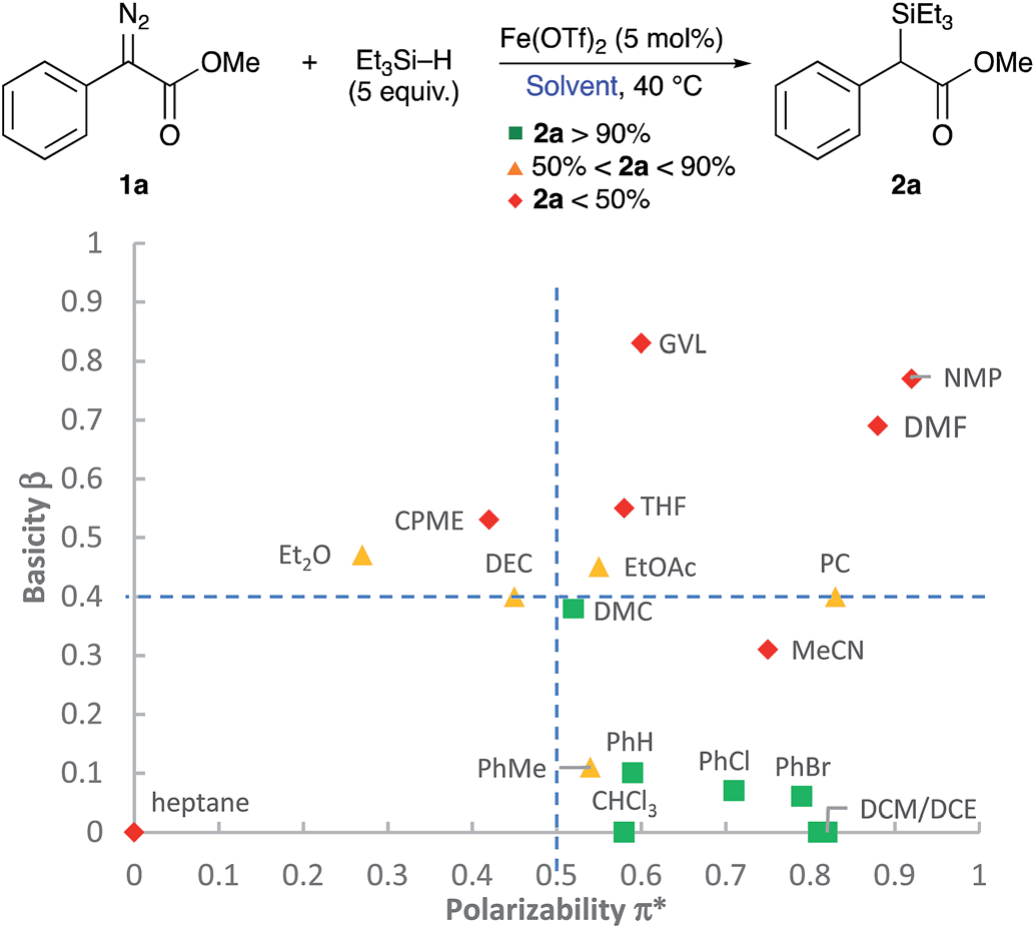
Kamlet–Taft plot of screened aprotic solvents. Reaction conditions: 1a (0.25 mmol), Fe(OTf)_2_ (5 mol%), Et_3_SiH (1.25 mmol), solvent (2 ml).

A broad range of acceptor/donor α-diazo compounds and silanes was examined to demonstrate the tolerance of this catalytic system (Table 2). Electron-donating groups in the *p*-position of the aryl group were tolerated as seen for cases 2b and 2c. Also, α-diazoesters with electron-withdrawing groups in the *o*-, *p*-, and *m*-positions of the aromatic ring (2d–h) allowed the reaction to proceed with good to excellent yields. Different ester functionalities were found to be well-tolerated (2i–k). Interestingly, the α-diazophosphonate 1l underwent complete conversion after 72 h to yield 48% of the corresponding insertion product 2l. The diazotrifluoromethyl substrates 1m and 1n efficiently underwent the insertion reaction to give rise to the α-silylated products in 75% and 90% yields, respectively. The Si–H insertion reaction of methyl α-phenyl-α-diazoacetate 1a was then conducted with different silanes. An insertion with PhMe_2_SiH led to an 82% yield of 2o with completion in 12 h. The reaction of the α-diazoester 1a with (2-naphthyl)Me_2_SiH achieved completion in 6 h with the corresponding product 2p in 76%. When tuning the aryl group of an ArMe_2_SiH series, it was observed that a *p*-OMe substituent resulted in an extended reaction time of 24 h with the corresponding silylated product in a 20% yield. An (*o*,*o*′-F)C_6_H_3_ substituted silane produced the insertion product 2r in 28%. A (*m*,*m*′-CF_3_)C_6_H_3_ substituted silane resulted in a moderate yield (50% of 2s) with completion in 6 h. Bulkier silanes, such as Ph_2_MeSiH and Ph_3_SiH, were tougher to insert where yields dramatically dropped to 36% and 30%, respectively, in addition to extended reaction times (up to 48 h). For alkyl silanes, the reaction of methyl α-phenyl-α-diazoacetate with (*t*-Bu)Me_2_SiH and (Hex)_3_SiH afforded the insertion products 2v and 2w in moderate yields (66% and 60%, respectively) previous attempts to establish the rate-determining step of an iron catalysed Si–H insertion reaction have been inconclusive.^23,24^ Unlike Si–H insertions with other metal catalysts, no H/D kinetic isotope effect was observed after conducting a competition experiment with a d-silane. The competition experiment between Et_3_Si–H and Et_3_Si–D was run again (Fig. 2, eqn (1)), but this time in DMC, using equimolar amounts of both silanes under the previously established optimum conditions. This experiment did not show any sign that the activation of the Si–H bond is rate-determining, where a value of 1.04 was obtained. Thus, we hypothesized that the extrusion of nitrogen to form the iron carbene is most likely the rate-determining step and that the activation of Si–H bond occurs quickly after the formation of the metal carbene.

**Table 2:** Screening of various acceptor/donor diazo compounds and silanes for the insertion reaction into Si–H bonds^a^

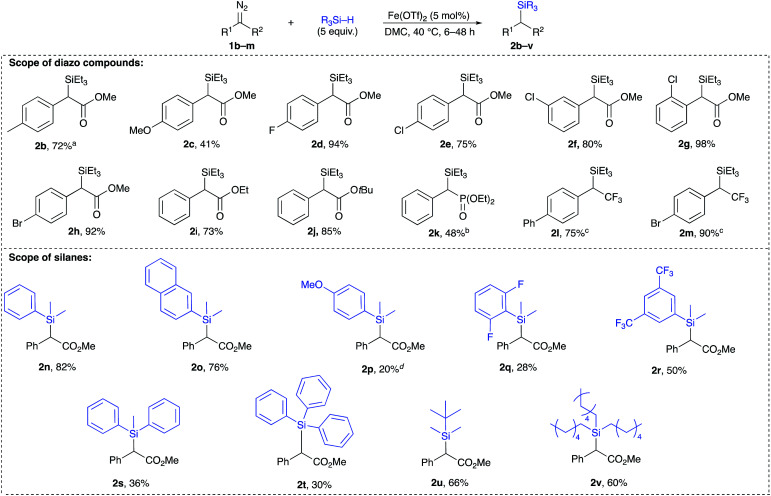

a Reaction conditions: diazo compound (0.25 mmol), Fe(OTf)_2_ (5 mol%), silane (1.25 mmol), DMC (2 ml), individual reaction time (see ESI).

b 10 equiv. of Et_3_SiH were used.

c 25 mg of 4 Å MS were used.

d Reaction was conducted at rt.^30^

e Yield calculated by ^1^H NMR.

**Fig. 2:**
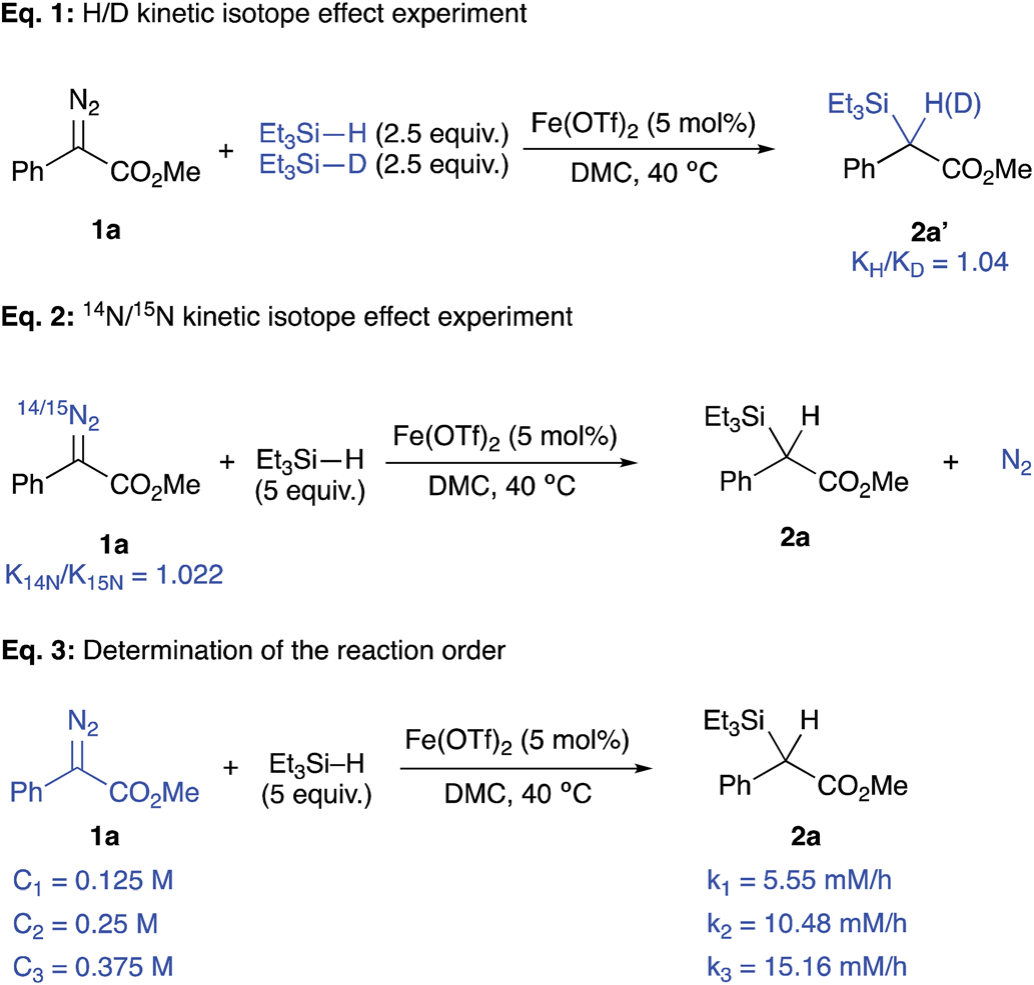
Kinetic studies for the determination of the rate-limiting step.

So, the kinetics of this reaction were placed under scrutiny in order to validate the hypothesis of a rate-determining step governed by the formation of the iron carbene species. In a study by Wu, a nitrogen kinetic isotope effect showed that the formation of a Rh carbene is rate-limiting for a Si–H insertion (Fig. 2, eqn (2)).^31^ Such a study was done by using isotope ratio mass spectrometry (IRMS) for the determination of a nitrogen kinetic isotope effect at natural abundance. The ratio of ^14^N/^15^N resulting from the natural ^15^N-enrichment of unreacted α-diazoester 1a during the progression of the insertion reaction was measured by IRMS. This ratio can be used to calculate the kinetic isotope effect from enrichment in the heavy isotope in a substrate.^32^ Given the fact that the extrusion of N_2_ is irreversible and that it is not involved in any subsequent step, the measured nitrogen ratios are only relevant to the carbene formation step.

Here, a large and normal heavy KIE value of 1.022 ± 0.007 was obtained, which supports the hypothesis that the formation of the iron carbene intermediate is rate-determining.^33^ Also, the insertion reaction was run under pseudo-first order conditions under the optimum conditions with different concentrations of the α-diazoester 1a (Fig. 2, eqn (3)). The formation of 2a was monitored by GC analysis and initial rates were found to be first-order with regard to the concentration of methyl α-phenyl-α-diazoacetate 1a. A linear variation was observed between the initial rate and the concentration of 1a, with [1a] = 0.125, 0.25, and 0.5 M, resulted in initial rates of 5.55, 10.48, and 15.16 mM h^−1^, respectively. Such results are in accordance with those obtained from a study of an iron-catalysed insertion reaction of diazo compounds into C–H bonds.^34^

With these results in hand, a mechanism can be therefore drawn where the coordination of the diazo species to the iron centre enriches it thus allowing π-back bonding to extrude nitrogen gas (I, Fig. 3). The formation of the Fe carbene II is rate-determining which subsequently reacts with the activated silane in a one-step manner to yield the insertion product 2a and regenerate the iron catalyst. An alternative mechanism involving the coordination of the terminal nitrogen to the metal centre has thus been refuted.^35^

**Fig. 3:**
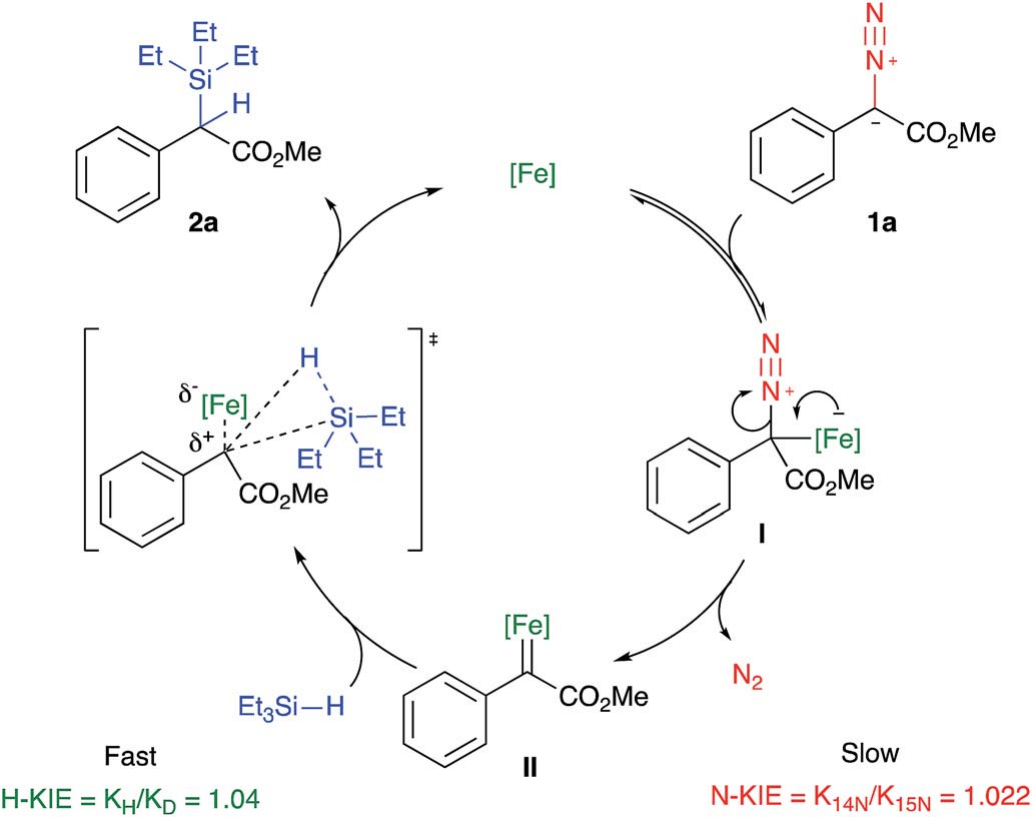
Plausible mechanism for the iron-catalysed insertion reaction of methyl α-phenyl-α-diazoacetate into Si–H bonds.

Polar X–H insertion reactions of methyl α-phenyl-α-diazoacetate 1a was then examined (Table 3). Insertions into O–H bonds were conducted in DMC where alcohol 3a and ethers 3b and 3c were obtained in very good yields with completion in 6 h.^36^ HOAc has been tested for O–H insertion and proceeds to give the corresponding ester 3d in an 88% yield. N–H insertions, however, were strongly dependent on the nature of the amine used. Secondary amines 3e and 3f were afforded in moderate yields of 57% and 68%, respectively, with prolonged reaction times (72 h) and even required increased temperatures (80 °C) to achieve completion. Improved yields were obtained with primary aromatic amines, where an insertion with aniline led to the corresponding product 3g in 84% yield but completion could only be reached after 72 h. Also, when using *o*-anisidine and *m*-anisidine, corresponding amines were obtained in 81% and 74%, respectively (Table 3, 3h and 3i). It is important to mention that in all of the three cases using primary amines, the reaction was selective toward a mono-insertion with no sign of a double insertion product. S–H insertions were mediated by an excess of the aryl thiol substrate and using 4 Å MS as an additive along with heating to reflux of DMC. The insertion reaction with thiophenol proceeded in a modest yield of 47% (Table 3, 3j). A higher yield was obtained when employing a *p*-OMe or a *p*-Br substituted thiophenol affording the corresponding products 3k and 3l in 93% and 98%, respectively. The reaction of 2a with benzyl thiol resulted in 3m in an 88% yield.

**Table 3:** Polar X–H (X = O, N, and S) insertion reactions with methyl α-phenyl-α-diazoacetate^a^

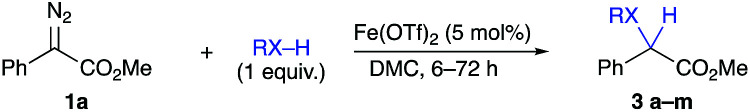
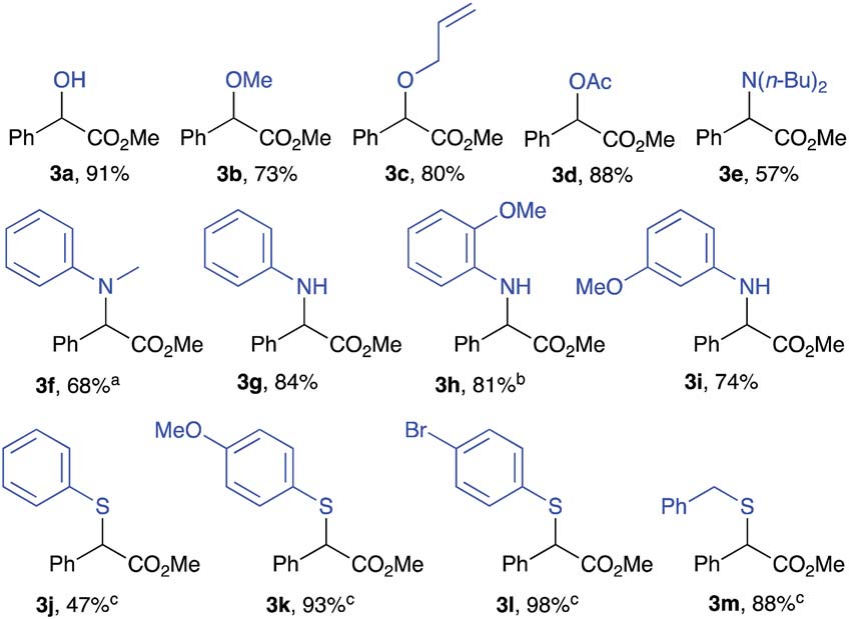

a Reaction conditions: methyl α-phenyl-α-diazoacetate (0.25 mmol), Fe(OTf)_2_ (5 mol%), X–H (0.25 mmol), DMC (2 ml).

b 5 equiv. of the amine were used and reaction was conducted at 80 °C.

c The reaction was concentrated to 0.5 M.

d 4 equiv. of the thiol and 25 mg of 4 Å MS were used at 80 °C.

In conclusion, we have successfully realized an iron-catalysed insertion reaction of α-diazo compounds into X–H bonds in DMC as a suitable solvent alternative. A wide range of α-silylesters were obtained in good to excellent yields. The mechanism of the insertion reaction into Si–H bonds was studied while showing that the formation of the iron carbene intermediate is rate-limiting. This work demonstrates the efficiency of iron carbenes used in insertion reactions and also shows that chlorinated solvents can be advantageously replaced by greener alternatives in diazo chemistry. Further developments in the use of iron carbenes will be reported in due course.

## Conflicts of interest

There are no conflicts to declare.

## Supplementary Material

RA-009-C9RA07203A-s001
