# Supplementary material for: FeII-catalysed insertion reaction of α-diazocarbonyls into X–H bonds (X = Si, S, N, and O) in dimethyl carbonate as a suitable solvent alternative
Source: RSC Adv. 2019 Oct 2;9(54):31241–6. doi: 10.1039/c9ra07203a (PMC9073380; doi:10.1039/c9ra07203a)
Supplement: RA-009-C9RA07203A-s001 [file RA-009-C9RA07203A-s001.pdf]

Electronic Supplementary Material (ESI) for RSC Advances.
This journal is © The Royal Society of Chemistry 2019

Supplementary Information for

Fe^{II}-Catalysed Insertion Reaction of α-Diazocarbonyls into X–H Bonds (X = Si, S, N, and O) in Dimethyl Carbonate as a Sustainable Solvent Alternative

Nour Tanbouza, Hoda Keipour and Thierry Ollevier*

Département de chimie, Université Laval, 1045 avenue de la Médecine

Québec, QC, G1V 0A6, Canada

Table of Contents

General information 2

Experimental details 3

General procedure for the $Fe(OTf)_2$-catalyzed insertion reaction of diazo compounds into Si–H bonds 3

Results of screening solvents for the insertion reaction of methyl α-phenyl-α-diazoacetate 1a with triethylsilane 3

General procedure for X–H insertion reaction into methyl α-phenyl-α-diazoacetate 1a. 9

Competitive H/D Kinetic Isotopic Experiment 12

Determination of the initial rate of the reaction 13

Determination of $^{14}N/^{15}N$ KIE 13

References 14

Copies of ^{1}H NMR, ^{13}C NMR, and ^{19}F NMR spectra 15

General information

All reactions were performed in flame-dried flasks and test tubes under an argon atmosphere. Dimethyl carbonate (Reagent plus®, 99%) was purchased from Sigma-Aldrich. $Fe(OTf)_2$ (98% purity) was purchased from Strem. All starting materials purchased from commercial suppliers were used without further purification. Thin-layer chromatography (TLC) was carried out on 250 µm commercial silica gel plates and compounds were visualized using UV absorbance and/or aqueous $KMnO_4$. Flash column chromatography was performed on Biotage (SNAP Ultra cartridge, 25g). ^{1}H, ^{13}C, and ^{19}F NMR spectra were recorded on a Bruker AC 300 MHz and a Varian Inova 400 MHz spectrometer in $CDCl_3$. For ^{1}H NMR (400 MHz), chemical shifts were reported downfield from tetramethylsilane (TMS) used as internal standard (δ = 0 ppm) and data are reported as follows: chemical shift (in ppm), multiplicity (s = singlet, d = doublet, t = triplet, q =quartet, m = multiplet), coupling constant (in Hz), and integration. High-resolution mass spectra (HRMS) were recorded on ESI-TOF (time-of-flight) mass spectrometer. IR spectra were recorded on a BOMEM Arid-Zone™ FT–IR spectrometer with a ZnSe ATR accessory and are reported in reciprocal centimeter (cm^{-1}). Coupling constant values (in Hertz) and number of protons for each signal are also indicated. Melting points (mp) are uncorrected and were recorded on a melting point apparatus. Gas chromatography measurements were conducted on an Agilent 6890N apparatus.

Isotopic analyses were performed at the Laboratoire d'Océanographie, Université Laval, Québec, Canada. Stable nitrogen isotope ratios were measured by a Thermo Electron Delta Advantage isotope ratio mass spectrometer in continuous-flow mode (Thermo Electron ConFlo III) using an ECS 4010 Elemental Analyzer/ZeroBlank Autosampler (Costech Analytical Technologies).

Caution! Diazo compounds should be handled with caution in a fume hood, as they are presumed to be toxic and potentially explosive. We recommend that their preparation be conducted behind a safety shield, although we have never experienced any explosive incident.

Experimental details

The diazo compounds (**1a–q**) were synthesized according to previously reported procedures.[1]

General procedure for the $Fe(OTf)_2$-catalyzed insertion reaction of diazo compounds into Si–H bonds

In a flame-dried test tube purged with argon and sealed with a septum, $Fe(OTf)_2$ (5 mol%) was dissolved in DMC (1 mL). The silane (1.25 mmol, 5 equiv) was then introduced *via* a syringe and the diazo compound (0.25 mmol, 1 equiv), dissolved in DMC (1 mL), was added dropwise. The reaction was monitored by TLC. After complete consumption of the diazo compound the crude mixture was passed through a pad of Celite and the pure product was obtained by silica gel flash chromatography on Biotage (SNAP Ultra cartridge, 25 g) using 9:1 hexanes/EtOAc as the eluent.

Results of screening solvents for the insertion reaction of methyl α-phenyl-α-diazoacetate 1a with triethylsilane

Entry	Solvent	β [a]	π* [a]	Time (h)	Yield 2a (%)
1	DMC	0.38	0.52	6	95
2	DEC	0.4	0.45	24	60
3	*n*-heptane	0	0	72	40
4	PC	0.4	0.83	24	65
5	EtOAc	0.45	0.55	24	53
6	CPME	0.53	0.42	24	40
7	GVL	0.83	0.6	1	–[b]
8	PhH	0.1	0.59	18	95
9	PhCl	0.07	0.71	18	96
10	PhBr	0.06	0.79	19	93
11	NMP	0.77	0.92	48	NR
12	DMF	0.69	0.88	72	(6)
13	H_2O	0.47	1.09	6	–
14	MeOH	0.66	0.6	6	–
15[c]	DCM	0	0.82	1	97
16[c]	$CHCl_3$	0	0.58	3	95
17[c]	DCE	0	0.81	3	91
18[c]	Et_2O	0.47	0.27	12	78
19[c]	PhMe	0.11	0.54	12	75
20[c]	MeCN	0.31	0.75	48	10
21[c]	THF	0.55	0.58	18	48

[a] Values were obtained from ref. 2, [b] Complete dimerization, [c] Solvents previously screened by us in ref. 1.

Methyl α-phenyl-α-triethylsilylacetate (2a).[2] The product was obtained according to the general procedure (reaction time = 6 h) as a colorless oil (63 mg, 0.23 mmol, 95% yield). ^{1}H NMR (400 MHz, $CDCl_3$): δ 7.38 (d, *J* = 7.1 Hz, 2H), 7.28 (t, *J* = 7.6 Hz, 2H), 7.16 (t, *J* = 7.3 Hz, 1H), 3.68 (s, 3H), 3.54 (s, 1H), 0.91 (t, *J* = 7.9 Hz, 9H), 0.64–0.57 (m, 6H) ppm; ^{13}C NMR (101 MHz, $CDCl_3$) δ 173.65, 136.53, 128.45, 128.10, 125.55, 51.34, 42.73, 7.04, 2.67 ppm; IR (ZnSe): 2951, 2878, 1720, 1497, 1313, 1144, 1005, 787 cm^{-1}.

Methyl α-(*p*-methylphenyl)-α-triethylsilylacetate (2b).[2] The product was obtained according to the general procedure (reaction time = 24 h) as a colorless oil (49 mg, 0.18 mmol, 72% yield). ^{1}H NMR (400 MHz, $CDCl_3$): δ 7.25 (d, *J* = 8.0 Hz, 2H), 7.11 (d, *J* = 8.0 Hz, 2H), 3.68 (s, 3H), 3.50 (s, 1H), 2.32 (s, 3H), 0.92 (t, *J* = 7.9 Hz, 9H), 0.64–0.55 (m, 6H) ppm; ^{13}C NMR (101 MHz, $CDCl_3$) δ 173.82, 135.00, 133.36, 128.81, 128.37, 51.30, 42.24, 20.94, 7.07, 2.70 ppm; IR (ZnSe): 2950, 2875, 1719, 1511, 1303, 1141, 1005, 818 cm^{-1}.

Methyl α-(*p*-methoxyphenyl)-α-triethylsilylacetate (2c).[2] The product was obtained according to the general procedure (reaction time = 48 h) as a colorless oil (30 mg, 0.10 mmol, 41% yield). ^{1}H NMR (400 MHz, $CDCl_3$): δ 7.26 (d, *J* = 8.8 Hz, 2H), 6.83 (d, *J* = 8.8 Hz, 2H), 3.78 (s, 3H), 3.67 (s, 3H), 3.47 (s, 1H), 0.91 (t, *J* = 7.9 Hz, 9H), 0.61–0.53 (m, 6H) ppm; ^{13}C NMR (101 MHz, $CDCl_3$) δ 174.00, 157.58, 129.42, 128.52, 113.54, 55.20, 51.30, 41.59, 7.06, 2.70 ppm; IR (ZnSe): 2953, 2878, 1720, 1511, 1332, 1146, 1005, 804 cm^{-1}.

Methyl α-(*p*-fluorophenyl)-α-triethylsilylacetate (2d).[2] The product was obtained according to the general procedure (reaction time = 6 h) as a colorless oil (66 mg, 0.23 mmol, 94% yield). ^{1}H NMR (400 MHz, $CDCl_3$): δ 7.33–7.30 (m, 2H), 6.96 (tt, *J* = 8.8, 2.0 Hz, 2H), 3.67 (s, 3H), 3.52 (s, 1H), 0.90 (t, *J* = 7.9 Hz, 9H), 0.61–0.54 (m, 6H) ppm; ^{13}C NMR (101 MHz, $CDCl_3$) δ 173.62, 161.10 (d, *J* = 243.8 Hz), 132.25 (d, *J* = 3.4 Hz), 128.80 (d, *J* = 7.6 Hz), 114.91 (d, *J* = 21.2 Hz), 51.36, 41.79, 6.99, 2.63 ppm; ^{19}F NMR (282 MHz, $CDCl_3$) δ –117.94 ppm; IR (ZnSe): 2953, 2878, 1720, 1506, 1435, 1146, 1013, 836 cm^{-1}.

Methyl α-(*p*-chlorophenyl)-α-triethylsilylacetate (2e).[2] The product was obtained according to the general procedure (reaction time = 6 h) as a colorless oil (56 mg, 0.18 mmol, 75% yield). ^{1}H NMR (400 MHz, $CDCl_3$): δ 7.29 (d, *J* = 8.6 Hz, 2H), 7.24 (d, *J* = 8.6 Hz, 2H), 3.68 (s, 3H), 3.51 (s, 1H),

0.90 (t, J = 7.9 Hz, 9H), 0.61–0.54 (m, 6H) ppm; ^{13}C NMR (101 MHz, $CDCl_3$) δ 173.34, 135.14, 131.33, 129.70, 128.19, 51.42, 42.11, 7.01, 2.61 ppm; IR (ZnSe): 2953, 2878, 1720, 1491, 1433, 1148, 1013, 820 cm^{-1}.

Methyl α-(*m*-chlorophenyl)-α-triethylsilylacetate (2f).[2] The product was obtained according to the general procedure (reaction time = 6 h) as a colorless oil (59 mg, 0.20 mmol, 80% yield). ^{1}H NMR (400 MHz, $CDCl_3$): δ 7.37 (t, J = 1.8 Hz, 1H), 7.24–7.13 (m, 3H), 3.68 (s, 3H), 3.51 (s, 1H), 0.91 (t, J = 7.9 Hz, 9H), 0.62–0.56 (m, 6H) ppm; ^{13}C NMR (101 MHz, $CDCl_3$) δ 173.10, 138.67, 133.93, 129.23, 128.34, 126.60, 125.72, 51.46, 42.51, 6.99, 2.61 ppm; IR (ZnSe): 2953, 2878, 1720, 1491, 1433, 1198, 1013, 910 cm^{-1}.

Methyl α-(*o*-chlorophenyl)-α-triethylsilylacetate (2g).[2] The product was obtained according to the general procedure (reaction time = 6 h) as a colorless oil (73 mg, 0.24 mmol, 98% yield). ^{1}H NMR (400 MHz, $CDCl_3$): δ 7.78 (dd, J = 7.9, 1.6 Hz, 1H), 7.33 (dd, J = 8.0, 1.3 Hz, 1H), 7.26 (t, J = 8.0 Hz, 1H), 7.12 (t, J = 8.0 Hz, 1H), 4.32 (s, 1H), 3.69 (s, 3H), 0.91 (t, J = 7.9 Hz, 9H), 0.66–0.60 (m, 6H) ppm; ^{13}C NMR (101 MHz, $CDCl_3$) δ 173.12, 134.52, 132.73, 130.84, 129.19, 126.72, 126.43, 51.46, 37.64, 6.99, 2.92 ppm; IR (ZnSe): 2951, 2876, 1720, 1329, 1196, 1145, 1005, 690 cm^{-1}.

Methyl α-(*p*-bromophenyl)-α-triethylsilylacetate (2h).[2] The product was obtained according to the general procedure (reaction time = 6 h) as a colorless oil (79 mg, 0.23 mmol, 92% yield). ^{1}H NMR (400 MHz, $CDCl_3$): δ 7.39 (d, J = 8.6 Hz, 2H), 7.24 (d, J = 8.6 Hz, 2H), 3.67 (s, 3H), 3.49 (s, 1H), 0.90 (t, J = 7.9 Hz, 9H), 0.61–0.54 (m, 6H) ppm; ^{13}C NMR (101 MHz, $CDCl_3$) δ 173.23, 135.68, 131.13, 130.09, 119.35, 51.44, 42.18, 7.02, 2.61 ppm; IR (ZnSe): 2951, 2876, 1719, 1489, 1265, 1145, 1012, 702 cm^{-1}.

Ethyl α-phenyl-α-triethylsilylacetate (2i).[2] The product was obtained according to the general procedure (reaction time = 6 h) as a colorless oil (51 mg, 0.18 mmol, 73% yield). ^{1}H NMR (400 MHz, $CDCl_3$): δ 7.37 (dd, J = 8.3, 1.2 Hz, 2H), 7.27 (t, J = 7.6 Hz, 2H), 7.16 (t, J = 7.3 Hz, 1H), 4.18–4.11 (m, 2H), 3.53 (s, 1H), 1.29 (t, J = 7.2 Hz, 3H), 0.92 (t, J = 7.9 Hz, 9H), 0.64–0.57 (m, 6H) ppm; ^{13}C NMR (101 MHz, $CDCl_3$) δ 173.18, 136.66, 128.49, 128.07, 125.49, 60.19, 42.86, 14.34, 7.08, 2.71 ppm; IR (ZnSe): 2954, 2878, 1720, 1496, 1305, 1107, 1007, 784 cm^{-1}.

***tert*-Butyl α-phenyl-α-triethylsilylacetate (2j).**[2] The product was obtained according to the general procedure (reaction time = 6 h) as a colorless oil (65 mg, 0.21 mmol, 85% yield). ^{1}H NMR (400 MHz, $CDCl_3$): δ 7.33 (dd, *J* = 8.3, 1.4 Hz, 2H), 7.27 (t, *J* = 7.7 Hz, 2H), 7.16 (t, *J* = 7.2 Hz, 1H), 3.44 (s, 1H), 1.49 (s, 9H), 0.93 (t, *J* = 7.9 Hz, 9H), 0.64–0.58 (m, 6H) ppm; ^{13}C NMR (101 MHz, $CDCl_3$) δ 172.36, 137.11, 128.70, 127.99, 125.36, 80.21, 43.70, 28.28, 28.02, 7.19, 2.88 ppm; IR (ZnSe): 2960, 2880, 1713, 1454, 1367, 1132, 1007, 843 cm^{-1}.

Diethyl (phenyl(triethylsilyl)methyl)phosphonate (2k).[2] The product was obtained according to the general procedure (reaction time = 48 h) as a colorless oil (41 mg, 0.12 mmol, 48% yield). ^{1}H NMR (400 MHz, $CDCl_3$): δ 7.24–7.13 (m, 5H), 4.05–3.99 (m, 2H), 3.90–3.77 (m, 2H), 2.77 (d, *J* = 25.4 Hz, 1H), 1.25 (t, *J* = 7.0 Hz, 5H), 1.06 (t, *J* = 7.3 Hz, 3H), 0.91 (t, *J* = 7.8 Hz, 13H), 0.75–0.59 (m, 6H) ppm; ^{13}C NMR (101 MHz, $CDCl_3$) δ 135.06 (d, *J* = 7.5 Hz), 129.52 (d, *J* = 8.1 Hz), 128.30 (d, *J* = 2.0 Hz), 125.57 (d, *J* = 2.7 Hz), 61.64 (dd, *J* = 105.5, 7.0 Hz), 33.95 (d, *J* = 125.8 Hz), 16.19 (dd, *J* = 8.0, 7.1 Hz), 7.28, 3.37 (d, *J* = 2.5 Hz); IR (ZnSe): 2982, 2906, 2075, 1599, 1293, 1163, 1014, 962, 856, 790 cm^{-1}.

(1-([1,1'-Biphenyl]-4-yl)-2,2,2-trifluoroethyl)triethylsilane (2l).[2] The product was obtained according to the general procedure (reaction time = 6 h) as a colorless oil (66 mg, 0.19 mmol, 75% yield). ^{1}H NMR (300 MHz, $CDCl_3$): δ 7.69–7.57 (m, 5H), 7.54–7.39 (m, 2H), 7.37–7.32 (m, 2H), 3.14 (q, *J* = 13.3 Hz, 1H), 1.03 (t, *J* = 13.3 Hz, 9H), 0.78–0.72 (m, 6H) ppm; ^{13}C NMR (101 MHz, $CDCl_3$) δ 140.53, 139.38, 133.33 (q, *J* = 3.4 Hz), 129.43, 128.78, 128.46 (q, *J* = 277.2 Hz), 127.30, 127.19, 126.94, 40.36 (q, *J* = 28.3 Hz), 7.11, 2.93 ppm; ^{19}F NMR (282 MHz, $CDCl_3$) δ –56.72 (d, *J* = 3.3 Hz) ppm; IR (ZnSe): 2956, 2880, 1489, 1306, 1132, 1008, 828 cm^{-1}.

(1-(4-Bromophenyl)-2,2,2-trifluoroethyl)triethylsilane (2m).[2] The product was obtained according to the general procedure (reaction time = 6 h) as a colorless oil (79 mg, 0.22 mmol, 90% yield). ^{1}H NMR (300 MHz, $CDCl_3$): 7.43 (d, *J* = 8.4 Hz, 2H), 7.07 (d, *J* = 8.4 Hz, 2H), 2.97 (q, *J* = 13.1 Hz, 1H), 0.92 (t, *J* = 7.8 Hz, 9H), 0.61 (qd, *J* = 7.7, 2.8 Hz, 6H) ppm; ^{13}C NMR (101 MHz, $CDCl_3$) δ 133.41 (q, *J* = 3.5 Hz), 131.67, 130.62, 129.08 (q, *J* = 277.9 Hz), 120.55, 40.34 (q, *J* = 28.5 Hz), 7.02, 2.79 ppm; ^{19}F NMR (282 MHz, $CDCl_3$) δ –56.99 (d, *J* = 4.1 Hz) ppm; IR (ZnSe): 2948, 2871, 1490, 1300, 1135, 1012, 921, 883 cm^{-1}.

Methyl α-phenyl-α-dimethyl(phenyl)silylacetate (2n).[2] The product was obtained according to the general procedure (reaction time = 18 h) as a colorless oil (58 mg, 0.20 mmol, 82% yield). ^{1}H NMR (300 MHz, $CDCl_3$): δ 7.43–7.36 (m, 5H), 7.21–7.18 (m, 4H), 7.19–7.12 (m, 1H), 3.66 (s, 1H), 3.58 (s, 3H), 0.41 (s, 3H), 0.38 (s, 3H) ppm; ^{13}C NMR (101 MHz, $CDCl_3$) δ 173.11, 135.96, 135.50, 134.03, 129.65, 128.37, 128.06, 127.71, 125.70, 51.31, 46.05, –4.07, –4.48 ppm; IR (ZnSe): 2950, 2876, 1716, 1287, 1140, 1001, 783, 765 cm^{-1}.

Methyl α-phenyl-α-dimethyl(2-naphthyl)silylacetate (2o). The product was obtained according to the general procedure (reaction time = 24 h) as a colorless viscous oil (63 mg, 0.19 mmol, 76% yield). ^{1}H NMR (400 MHz, $CDCl_3$) δ 7.87 (s, 1H), 7.85–7.76 (m, 4H), 7.54–7.47 (m, 3H), 7.43 (dd, *J* = 8.2, 1.1 Hz, 2H), 7.25–7.12 (m, 4H), 3.69 (s, 1H), 3.54 (s, 2H), 0.45 (s, 3H), 0.41 (s, 3H) ppm; ^{13}C NMR (101 MHz, $CDCl_3$) δ 173.13, 135.94, 135.07, 133.93, 133.02, 132.72, 129.96, 128.41, 128.18, 128.09, 127.71, 126.88, 126.68, 126.01, 125.74, 51.36, 46.06, –3.94, –4.32 ppm; IR (ZnSe): 3086, 3027, 3002, 2951, 2838, 2360, 2340, 1718, 1146, 1086, 857, 831, 805 cm^{-1}; HRMS (ESI-TOF) calculated for $C_{21}H_{22}O_2Si^+$ [M + NH_4^+]: 334.1389, found 334.1393.

Methyl α-phenyl-α-dimethyl(*o,o′*-difluorophenyl)silylacetate (2q). The product was obtained according to the general procedure (reaction time = 6 h) as a colorless oil (23 mg, 0.07 mmol, 28% yield). ^{1}H NMR (400 MHz, $CDCl_3$) δ 7.39–7.27 (m, 1H), 7.32–7.25 (m, 2H), 7.23 (m, 2H), 7.20–7.10 (m, 1H), 3.83 (s, 1H), 3.57 (s, 3H), 0.48 (t, *J* = 2.0 Hz, 3H), 0.36 (t, *J* = 1.8 Hz, 3H) ppm; ^{13}C NMR (101 MHz, $CDCl_3$) 172.72, 167.06 (d, *J* = 244.9 Hz), 166.92 (d, *J* = 244.9 Hz), 135.55, 132.76 (t, *J* = 10.8), 128.39, 128.10, 125.86, 111.00 (dd, *J* = 27.6, 2.9), 51.40, 45.18, –2.24, –2.44 ppm; ^{19}F NMR (282 MHz, $CDCl_3$) δ –96.35 ppm; IR (ZnSe): 3090, 3034, 2995, 2361, 2345, 1610, 1445, 1248, 1219, 976, 835, 789 cm^{-1}; HRMS (ESI-TOF) calculated for $C_{17}H_{18}F_2O_2Si^+$ [M + H^+]: 321.1122, found 321.1130.

Methyl α-phenyl-α-dimethyl(*m,m′*-di(trifluoromethyl)phenyl)silylacetate (2r). The product was obtained according to the general procedure (reaction time = 6 h) as a colorless oil (53 mg, 0.12 mmol, 50% yield). ^{1}H NMR (400 MHz, $CDCl_3$) δ 7.86 (s, 1H), 7.71 (s, 2H), 7.23 (m, 3H), 7.17 (t, *J* = 7.0 Hz, 2H), 3.60 (s, 1H), 3.59 (s, 3H), 0.45 (s, 3H), 0.41 (s, 3H) ppm; ^{13}C NMR (101 MHz, $CDCl_3$) δ 172.42, 139.28, 134.84, 133.80, 130.01 (q, *J* = 33.0 Hz), 128.35, 128.21, 126.24, 123.43 (q, *J* =

272.6 Hz), 123.27 (q, J = 3.8 Hz), 51.50, 45.32, –4.37, –4.68 ppm; ^{19}F NMR (282 MHz, $CDCl_3$) δ – 62.91 ppm; IR (ZnSe): 3034, 2957, 2341, 1742, 1414, 1277, 1105, 1097, 1070, 851 cm^{-1}; HRMS (ESI-TOF) calculated for $C_{19}H_{18}F_6O_2Si^+$ [M + H^+]: 420.0980, found 420.0994.

Methyl α-phenyl-α-methyldiphenylsilylacetate (2s).[2] The product was obtained according to the general procedure (reaction time = 24 h) as a colorless oil (32 mg, 0.09 mmol, 36% yield). ^{1}H NMR (400 MHz, $CDCl_3$): δ 7.62–7.58 (m, 2H), 7.46–7.31 (m, 6H), 7.31–7.21 (m, 2H), 7.20–7.17 (m, 5H), 4.03 (s, 1H), 3.48 (s, 3H), 0.62 (s, 3H) ppm; ^{13}C NMR (101 MHz, $CDCl_3$) δ 172.99, 135.05, 134.96, 134.84, 129.82, 129.71, 129.53, 128.81, 128.03, 127.97, 127.78, 125.85, 51.40, 44.79, 5.24 ppm; IR (ZnSe): 2946, 1712, 1427, 1195, 1104, 1009, 900, 790, 693 cm^{-1}.

Methyl α-phenyl-α-*tert*-butyldimethylsilylacetate (2u). The product was obtained according to the general procedure (reaction time = 6 h) as a colorless oil (44 mg, 0.16 mmol, 66% yield). ^{1}H NMR (400 MHz, $CDCl_3$): δ 7.37 (m, 2H), 7.27 (m, 2H), 7.17 (tt, J = 7.4, 2.0 Hz, 1H), 3.66 (s, 3H), 3.56 (s, 1H), 0.87 (s, 9H), 0.11 (s, 3H), –0.12 (s, 3H) ppm; ^{13}C NMR (100 MHz, $CDCl_3$) δ 173.77, 137.00, 128.71, 128.09, 125.66, 51.39, 42.92, 26.60, 17.79, –6.50, –6.97 ppm; IR (ZnSe): 2957, 2853, 2360, 2098, 1718, 1409, 1182, 962, 810 cm^{-1}; HRMS (ESI-TOF) calculated for $C_{15}H_{24}O_2Si^+$ [M + H^+]: 265.1618, found 265.1619.

Methyl α-phenyl-α-tri-*n*-hexylsilylacetate (2v). The product was obtained according to the general procedure (reaction time = 6 h) as a colorless oil (64 mg, 0.15 mmol, 60%). ^{1}H NMR (400 MHz, $CDCl_3$) δ 7.33 (m, 2H), 7.26 (m, 2H), 7.16 (tt, J = 7.3, 2.1 Hz, 1H), 3.66 (s, 3H), 3.51 (s, 1H), 1.34–1.15 (m, 24H), 0.88 (t, J = 6.9 Hz, 9H), 0.63–0.48 (m, 6H) ppm; ^{13}C NMR (100 MHz, $CDCl_3$) δ 173.67, 136.61, 128.42, 128.05, 125.50, 51.28, 43.31, 33.41, 31.39, 23.30, 22.56, 14.10, 11.58 ppm; IR (ZnSe): 2952, 2858, 1718, 1145, 837, 822, 808, 700 cm^{-1}, HRMS (ESI-TOF) calculated for $C_{27}H_{48}O_2Si^+$ [M + H^+]: 455.3315, found 455.3300.

General procedure for X–H insertion reaction into methyl α-phenyl-α-diazoacetate 1a.

In a flame-dried test tube purged with argon and sealed with a septum, $Fe(OTf)_2$ (5 mol%, 4.4 mg) was dissolved in DMC (1 ml). The X–H insertion substrate (1 equiv.) was then introduced via a syringe and the diazo compound (44 mg, 0.25 mmol), dissolved in DMC (1 ml), was added dropwise. The reaction was monitored by TLC. After complete consumption of the diazo compound the crude mixture was passed through a pad of Celite and the pure product was obtained by silica gel flash chromatography on Biotage (SNAP Ultra cartridge, 25 g).

Methyl 2-hydroxy-2-phenylacetate (3a).[3] The product was obtained according to the general procedure (reaction time = 6 h) as a colorless oil (38 mg, 0.23 mmol, 91% yield). ^{1}H NMR (300 MHz, $CDCl_3$): δ 7.44–7.31 (m, 5H), 5.18 (s, 1H), 3.77 (s, 3H); ^{13}C NMR (101 MHz, $CDCl_3$) δ 174.11, 138.25, 128.61, 128.50, 126.60, 72.90, 53.01 ppm; IR (ZnSe): 3446, 2858, 1721, 1494, 1456, 1382, 1310, 1190, 1062, 934, 872 cm^{-1}.

Methyl 2-methoxy-2-phenylacetate (3b).[3] The product was obtained according to the general procedure (reaction time = 6 h) as a colorless oil (33mg, 0.18 mmol, 73%). ^{1}H NMR (300 MHz, $CDCl_3$): δ 7.46–7.33 (m, 5H), 4.78 (s, 1H), 3.72 (s, 3H), 3.41 (s, 3H); ^{13}C NMR (101 MHz, $CDCl_3$): δ 171.12, 136.12, 128.77, 128.66, 127.20, 82.54, 57.35, 52.31 ppm; IR (ZnSe): 3032, 2829, 2127, 1749, 1492, 1353, 1210, 1171, 1110, 958, 847 cm^{-1}.

Methyl 2-(allyloxy)-2-phenylacetate (3c).[3] The product was obtained according to the general procedure (reaction time = 6 h) as a colorless oil (41 mg, 0,2 mmol, 80%). ^{1}H NMR (300 MHz, $CDCl_3$): δ 7.47–7.44 (m, 2H), 7.39–7.33 (m, 3H), 6.00–5.87 (m, 1H), 5.32–5.21 (m, 2H), 4.95 (s, 1H), 4.06 (d, *J* = 5.8 Hz, 2H), 3.71 (s, 3H); ^{13}C NMR (101 MHz, $CDCl_3$): δ 171.27, 136.30, 133.73, 128.70, 128.63, 127.29, 118.24, 79.69, 70.42, 52.27 ppm; IR (ZnSe): 3080, 2953, 2360, 1749, 1494, 1343, 1171, 1014, 926, 699 cm^{-1}.

Methyl 2-acetoxy-2-phenylacetate (3d).[4] The product was obtained according to the general procedure (reaction time = 6 h) as a colorless oil (46 mg, 0.22 mmol, 88%). ^{1}H NMR (400 MHz, $CDCl_3$): δ 7.49–7.45 (m, 1H), 7.44–7.33 (m, 2H), 7.18 (d, *J* = 11.3 Hz, 1H), 7.13–7.04 (m, 1H), 5.94

(s, 1H), 3.73 (s, 3H), 2.20 (s, 3H); ^{13}C NMR (101 MHz, $CDCl_3$): δ 170.31, 169.31, 133.74, 129.65, 128.79, 127.63, 74.43, 52.62, 20.72 ppm.

Methyl 2-(di-*n*-butylamino)-2-phenylacetate (3e).[5] The product was obtained according to the general procedure (reaction time = 72 h) as a yellow oil (39.5 mg, 0.14 mmol, 57%). ^{1}H NMR (300 MHz, $CDCl_3$): δ 7.39–7.36 (m, 2H), 7.35–7.26 (m, 3H), 4.57 (s, 1H), 3.72 (s, 3H), 2.54 (t, *J* = 7.4 Hz, 4H), 1.44–1.34 (m, 4H), 1.29–1.17 (m, 4H), 0.83 (t, *J* = 7.3 Hz, 6H); ^{13}C NMR (101 MHz, $CDCl_3$): δ 172.94, 137.32, 128.74, 128.20, 127.70, 68.87, 51.44, 50.40, 29.66, 20.35, 13.97 ppm; IR (ZnSe): 3060, 2985, 1730, 1611, 1354, 1178, 1005, 837, 812, 808, 700 cm^{-1}.

Methyl 2-(methyl(phenyl)amino)-2-phenylacetate (3f).[5] The product was obtained according to the general procedure (reaction time = 24 h) as a pale-yellow solid (43 mg, 0.17 mmol, 68%). ^{1}H NMR (300 MHz, $CDCl_3$): δ 7.41–7.34 (m, 3H), 7.31–7.25 (m, 4H), 6.88 (d, *J* = 8.8 Hz, 2H), 6.81 (t, *J* = 7.3 Hz, 1H), 5.67 (s, 1H), 3.79 (s, 3H), 2.80 (s, 3H); ^{13}C NMR (101 MHz, $CDCl_3$): δ 172.35, 149.87, 137.68, 129.31, 128.68, 128.42, 128.12, 118.09, 113.46, 60.73, 65.72, 52.01, 43.49 ppm; IR (ZnSe): 3063, 3027, 2951, 2884, 1734, 1595, 1452, 1291, 1168, 1004, 988, 951, 766 cm^{-1}.

Methyl 2-phenyl-2-(phenylamino)acetate (3g).[5] The product was obtained according to the general procedure (reaction time = 72 h) as a pale-yellow solid (50 mg, 0.21 mmol, 83% yield). ^{1}H NMR (400 MHz, $CDCl_3$) δ 7.49 (d, *J* = 7.2 Hz, 2H), 7.41–7.28 (m, 3H), 7.17–7.06 (m, 3H), 6.70 (t, *J* = 7.3 Hz, 1H), 6.56 (d, *J* = 8.1 Hz, 2H), 5.08 (d, *J* = 5.8 Hz, 1H), 4.96 (*br*-s, 1H), 3.74 (s, 3H) ppm; ^{13}C NMR (101 MHz, $CDCl_3$): δ 172.33, 145.95, 137.64, 129.26, 128.90, 128.33, 127.27, 118.14, 113.42, 60.75, 52.82 ppm; IR (ZnSe): 3401, 3062, 3030, 2892, 1730, 1605, 1431, 1319, 1176, 1014, 873, 737 cm^{-1}.

Methyl 2-((2-methoxyphenyl)amino)-2-phenylacetate (3h).[5] The product was obtained according to the general procedure (reaction time = 6 h) as a brown solid (57 mg, 0.21 mmol, 84% yield). ^{1}H NMR (400 MHz, $CDCl_3$) δ 7.52–7.50 (m, 2H), 7.38–7.30 (m, 3H), 6.79–6.57 (m, 3H), 6.35 (dd, *J* = 7.7, 1.6 Hz, 1H), 5.47 (s, 1H), 5.08 (s, 1H), 3.89 (s, 3H), 3.74 (s, 3H) ppm; ^{13}C NMR (101 MHz, $CDCl_3$) δ 172.28, 147.01, 137.68, 135.92, 128.82, 128.23, 127.25, 121.00, 117.36, 110.59, 109.52, 60.73, 55.45, 52.72 ppm; IR (ZnSe): 3406, 3060, 3001, 1736, 1593, 1454, 1171, 1003, 730 cm^{-1}.

Methyl 2-((3-methoxyphenyl)amino)-2-phenylacetate (3i).[5] The product was obtained according to the general procedure (reaction time = 24 h) as a colorless oil (50 mg, 0.18 mmol, 74% yield). ^{1}H NMR (300 MHz, $CDCl_3$) δ 7.50–7.42 (m, 2H), 7.38–7.29 (m, 3H), 7.03 (t, *J* = 8.1 Hz, 1H), 6.27 (ddd, *J* = 8.2, 2.4, 0.8 Hz, 1H), 6.22–6.15 (m, 1H), 6.11 (t, *J* = 2.3 Hz, 1H), 5.07 (d, *J* = 5.6 Hz, 1H), 4.98 (d, *J* = 5.6 Hz, 1H), 3.73 (s, 3H), 3.71 (s, 3H) ppm; ^{13}C NMR (101 MHz, $CDCl_3$) δ 172.25, 160.67, 147.29, 137.57, 130.00, 128.89, 128.32, 127.21, 106.39, 103.33, 99.50, 60.71, 55.01, 52.82 ppm; IR (ZnSe): 3408, 3062, 2952, 1736, 1596, 1494, 1161, 1001, 727 cm^{-1}.

Methyl 2-phenyl-2-(phenylthio)acetate (3j).[6] The product was obtained according to the general procedure (reaction time = 6 h) as colorless oil. (31 mg, 0.11 mmol, 47% yield). ^{1}H NMR (400 MHz, $CDCl_3$) δ 7.46–7.43 (dt, *J* = 7.6, 1.8 Hz, 2H), 7.39–7.34 (m, 3H), 7.34–7.29 (m, 3H), 7.28–7.26 (m, 2H), 4.92 (s, 1H), 3.68 (s, 3H) ppm; ^{13}C NMR (101 MHz, $CDCl_3$) δ 170.88, 135.57, 133.67, 132.65, 128.97, 128.68, 128.49, 128.33, 128.02, 56.34, 52.74 ppm; IR (ZnSe): 2951, 2876, 1717, 1489, 1265, 1145, 1089, 1011, 705 cm^{-1}.

Methyl 2-((4-methoxyphenyl)thio)-2-phenylacetate (3k).[6] The product was obtained according to the general procedure (reaction time = 6 h) as a colorless oil (70 mg, 0.23 mmol, 93% yield). ^{1}H NMR (400 MHz, $CDCl_3$) δ 7.40–7.38 (m, 2H), 7.33–7.28 (m, 5H), 6.80–6.78 (m, 2H), 4.76 (s, 1H), 3.79 (s, 3H), 3.67 (s, 3H) ppm; ^{13}C NMR (101 MHz, $CDCl_3$) δ 159.92, 136.22, 132.66, 128.59, 128.42, 128.20, 123.61, 114.63, 57.40, 55.37, 52.60 ppm; IR (ZnSe): 3005, 2961, 2938, 2837, 1573, 1510, 1285, 1259, 1030, 829, 775 cm^{-1}.

Methyl 2-((4-bromophenyl)thio)-2-phenylacetate (3l).[7] The product was obtained according to the general procedure (reaction time = 6 h) as a white solid (83 mg, 0.24 mmol, 98% yield). ^{1}H NMR (400 MHz, $CDCl_3$): δ 7.42–7.39 (m, 4H), 7.35–7.29 (m, 3H), 7.24–7.22 (m, 2H), 4.86 (s, 1H), 3.69 (s, 3H) ppm; ^{13}C NMR (101 MHz, $CDCl_3$) δ 170.59, 135.25, 134.42, 134.27, 131.92, 129.12, 128.12, 128.48, 128.24, 56.43, 52.81 ppm; IR (ZnSe): 2945, 1732, 1484, 1428, 1301, 1156, 1092, 1004, 831, 727, 695 cm^{-1}.

Methyl 2-(benzylthio)-2-phenylacetate (3m).[6] The product was obtained according to the general procedure (reaction time = 6 h) as a colorless oil. (60 mg, 0.22 mmol, 88% yield). ^{1}H NMR (400 MHz, $CDCl_3$) δ 7.42–7.39 (m, 2H), 7.36–7.24 (m, 8H), 4.43 (s, 1H), 3.77 (d, *J* = 13.5 Hz, 1H),

3.69 (s, 3H), 3.62 (d, J = 13.5 Hz, 1H) ppm; ^{13}C NMR (101 MHz, $CDCl_3$) δ 171.11, 137.09, 135.75, 129.02, 128.68, 128.59, 128.53, 128.20, 127.26, 52.69, 51.48, 36.21 ppm; IR (ZnSe): 2953, 2872, 1703, 1471, 1255, 1141, 1083, 1013, 708, 695 cm^{-1}.

Competitive H/D Kinetic Isotopic Experiment

In a flame-dried test tube purged with argon and sealed with a septum, $Fe(OTf)_2$ (5 mol%, 4.4 mg) was dissolved in DMC (1 mL). Equimolar amounts of Et_3Si–H (2.5 equiv.) and Et_3Si–D (2.5 equiv.) were then introduced *via* a syringe and the diazo compound (44 mg, 0.25 mmol), dissolved in DMC (1 mL), was added dropwise over 1 minute. The reaction was monitored by TLC. After complete consumption of the diazo compound the crude mixture was passed through a pad of Celite and silica. An H/D KIE value of 1.04 was obtained.

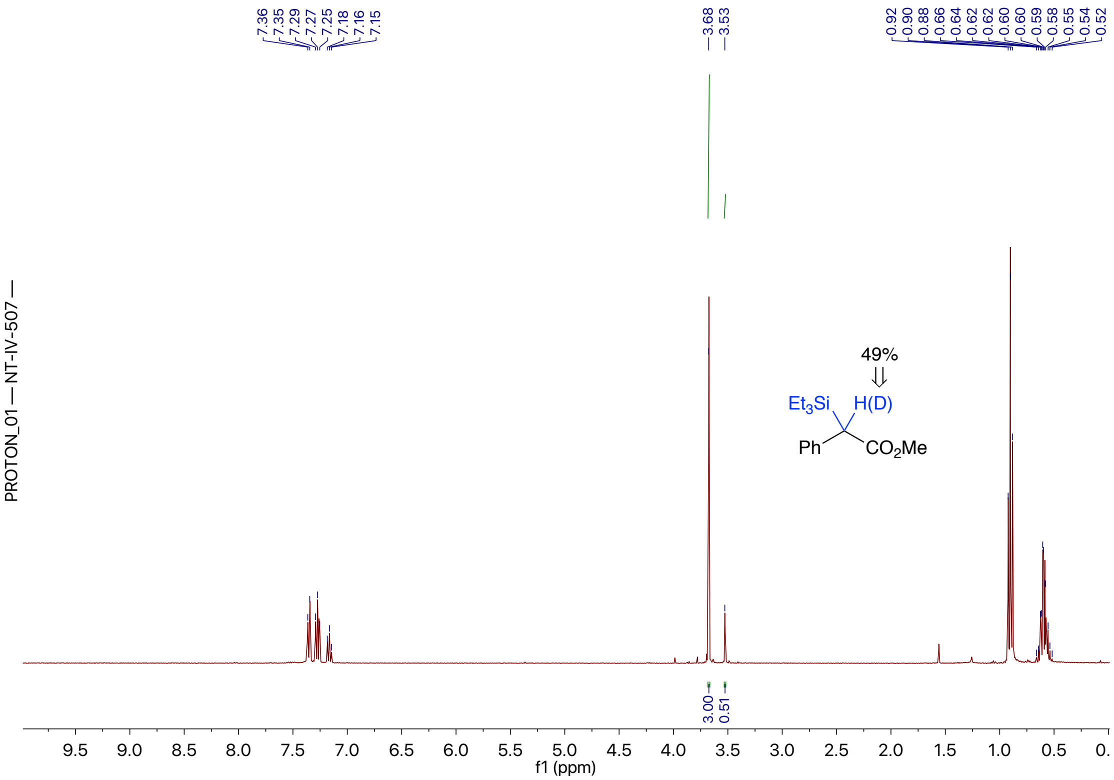

Determination of the initial rate of the reaction

The initial rate of the reaction was established by running the model reaction under pseudo-first order conditions with respect to the diazo compound. The concentration of the insertion product was determined via GC analysis using phenyl methylacetate as an internal standard. The initial rates were found to be 5.55, 10.48, and 15.16 mM/h for 0.125, 0.25, and 0.375 M of the diazo ester respectively.[7]

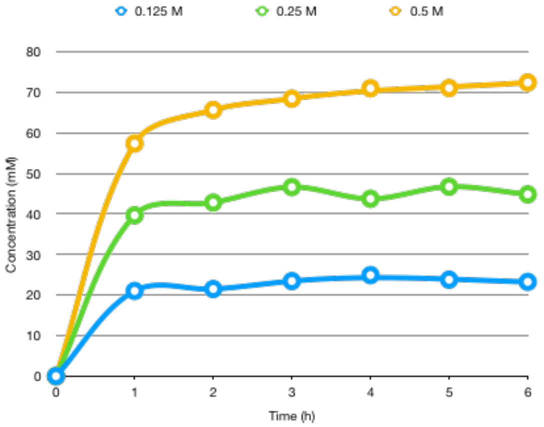

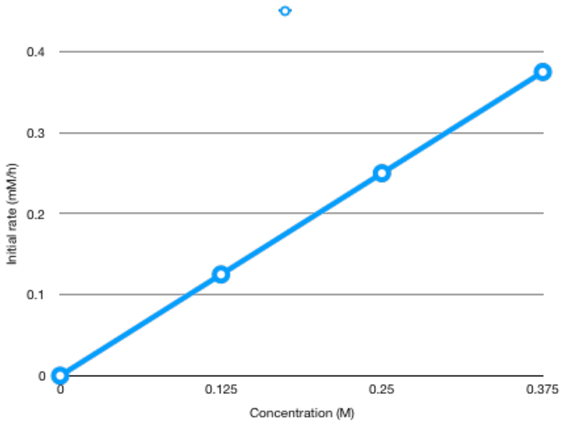

Determination of $^{14}N/^{15}N$ KIE

The model reaction was run to incomplete conversion with fractions (1-f) varying from 0.38 to 0.66. The remaining diazo compound was isolated by column chromatography (9:1 hexanes/EtOAc). 1 μL of the diazo was sampled on Chromosorb, loaded into tin capsules, and combusted (localized temperature up to 1800 °C) for the determination of nitrogen isotopic ratios (d15N (‰)).[8]

d15N (‰)	R_0	1-f	d15N (‰)	R	KIE
-13.30	0.986703	0.387	1.16	1.001157	1.015441
-13.15	0.986851	0.387	1.11	1.001113	1.015394
-13.17	0.986834	0.662	0.30	1.000296	1.034032
-13.16	0.986844	0.662	0.36	1.000357	1.034189
**$R_{0(av)}$**	0.986808	0.41	0.76	1.00076	1.015999
		0.41	0.76	1.000761	1.016
		0.453	5.53	1.005535	1.024318
		0.453	5.54	1.005543	1.024329
				**$KIE_{(av)}$**	1.022462
				**SD**	0.007592

References

1. (a) H. Keipour and T. Ollevier, *Org. Lett.*, 2017, **19**, 5736-5739. (b) D. Chen, D.-X. Zhu and M.-H. Xu, *J. Am. Chem. Soc.*, 2016, **138**, 1498-1501.
2. (a) Y. Marcus, *Chem. Soc. Rev.*, 1993, **22**, 409-416. (b) J.-L. M. Abboud and R. Notario, *Pure Appl. Chem.*, 1999, **71**, 645-718.
3. S.-F. Zhu, Y. Cai, H.-X. Mao, J.-H. Xie and Q.-L. Zhou, *Nat. Chem.*, 2010, **2**, 546-551.
4. X. Creary and C. C. Geiger, *J. Am. Chem. Soc.,* 1982, **104**, 4151-4162.
5. (a) T. Miura, M. Morimoto and M. Murakami, *Org. Lett.*, 2012, **14**, 5214–5217. (b) B. J. Anding and L. K. Woo, *Organometallics*, 2013, **32**, 2599-2607.
6. H. Keipour, A. Jalba, N. Tanbouza, V. Carreras and T. Ollevier, *Org. Biomol. Chem.*, 2019, **17**, 3098–3102.
7. H. M. Mbuvi and L. K. Woo, *Organometallics*, 2008, **27**, 637–645.
8. F. M. Wong, J. Wang, A. C. Hengge and W. Wu, *Org. Lett.*, 2007, **9**, 1663-1665.

Copies of ^{1}H NMR, ^{13}C NMR, and ^{19}F NMR spectra

Methyl α-phenyl-α-triethylsilylacetate (2a)

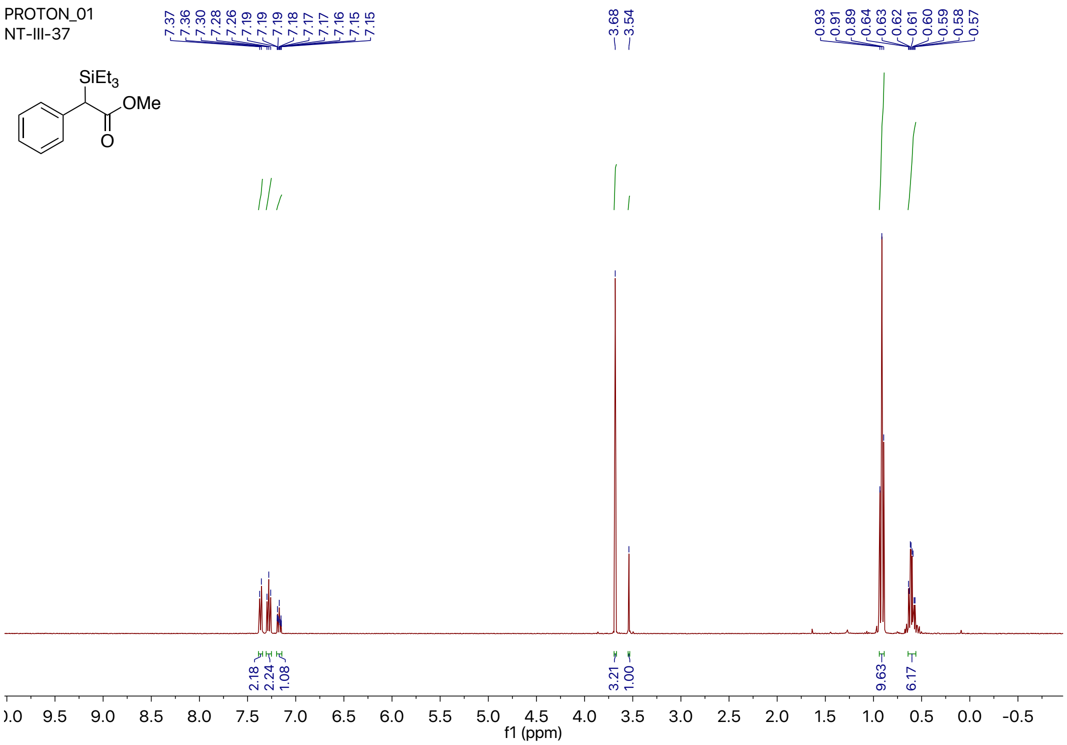

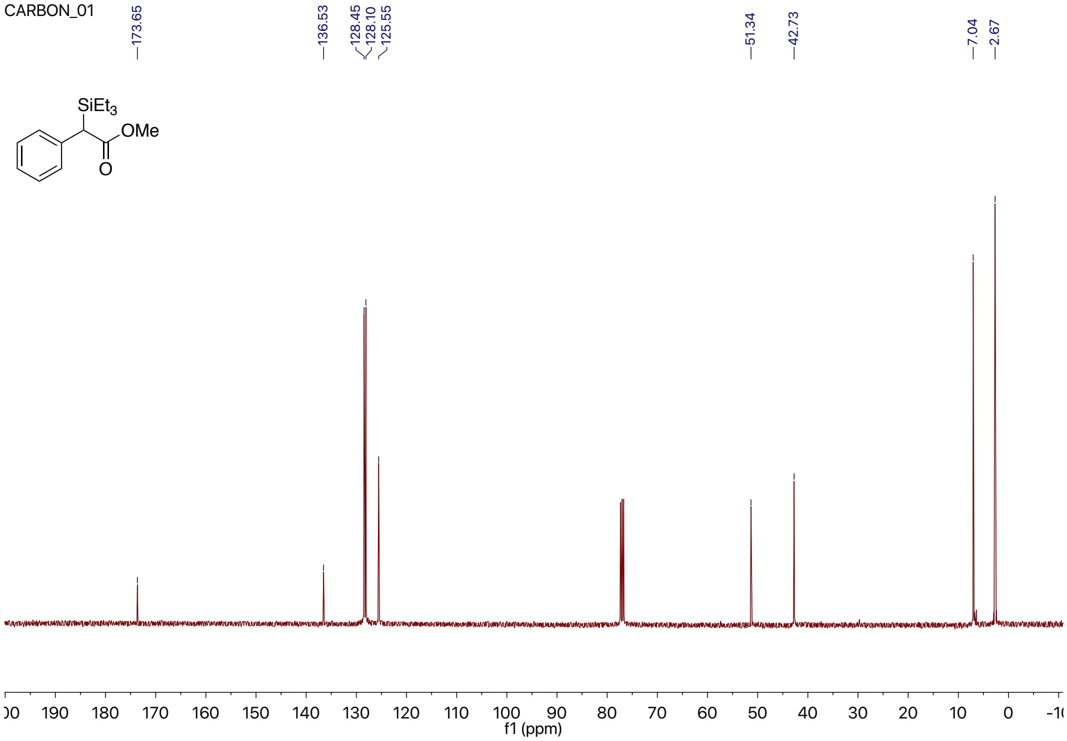

Methyl α-(*p*-methylphenyl)-α-triethylsilylacetate (2b)

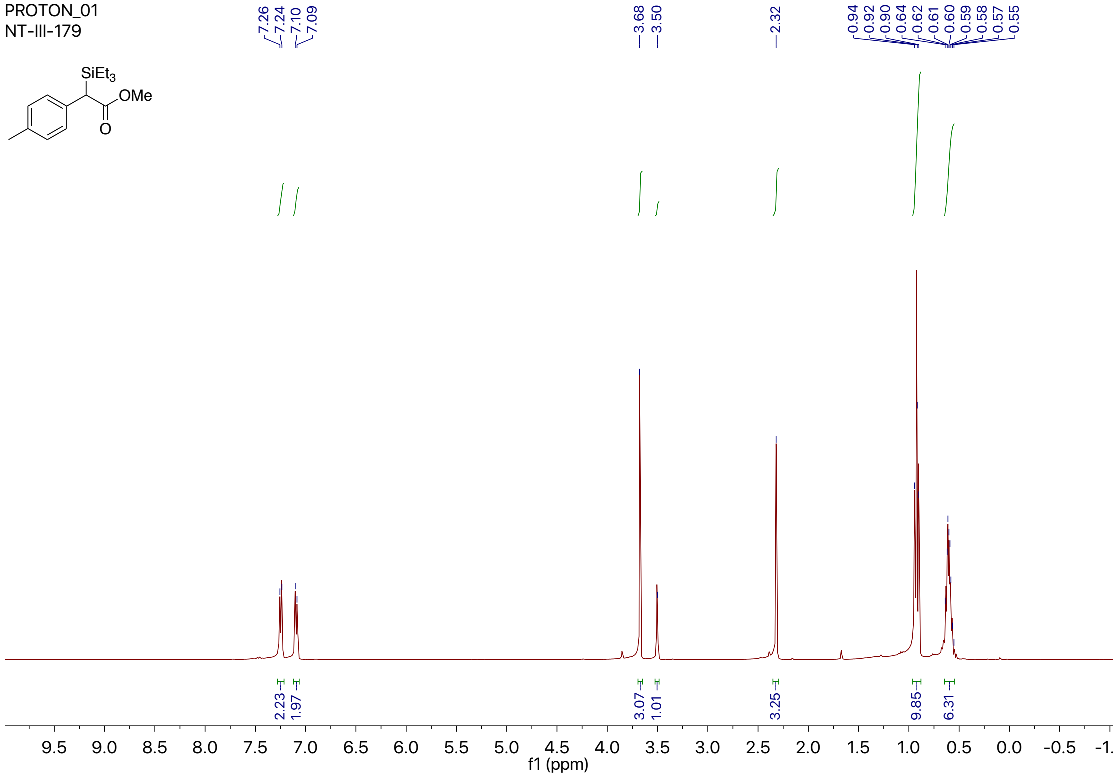

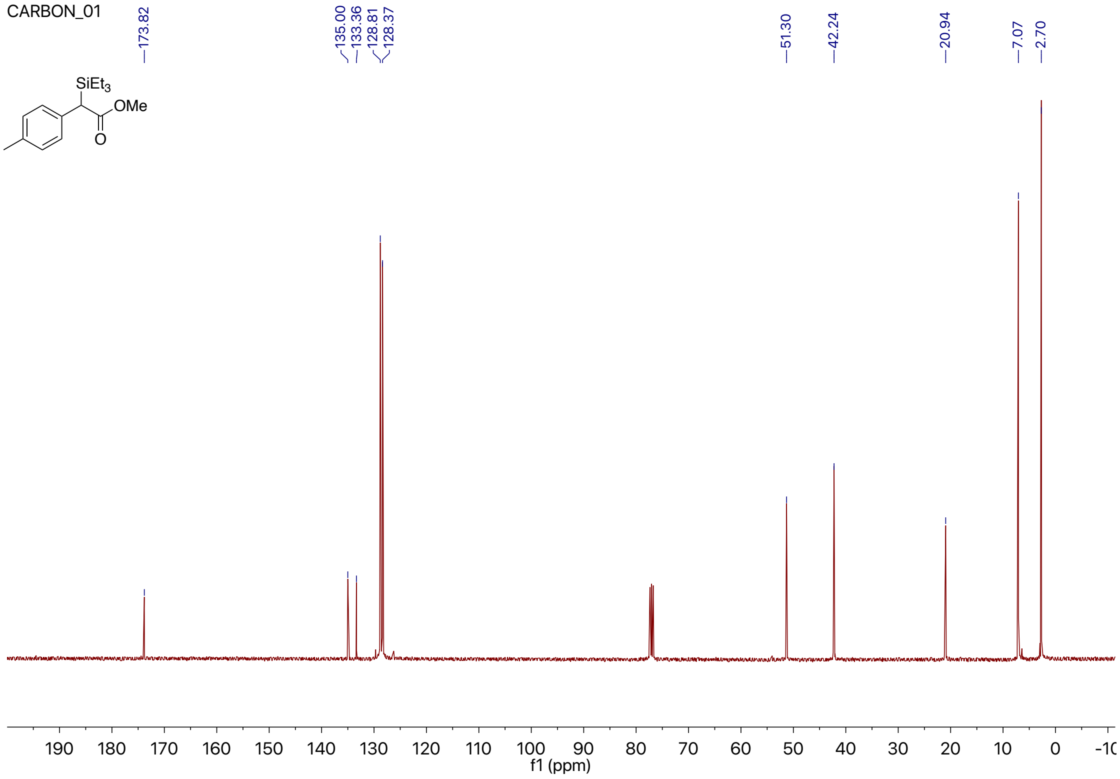

Methyl α-(*p*-methoxyphenyl)-α-triethylsilylacetate (2c)

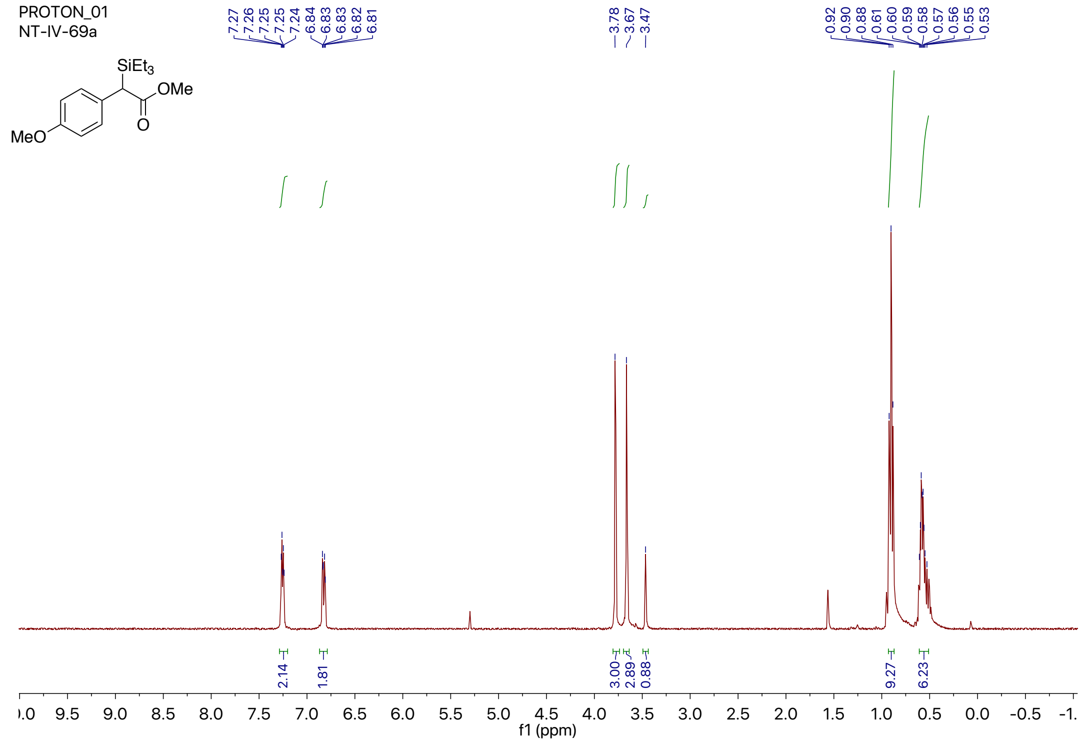

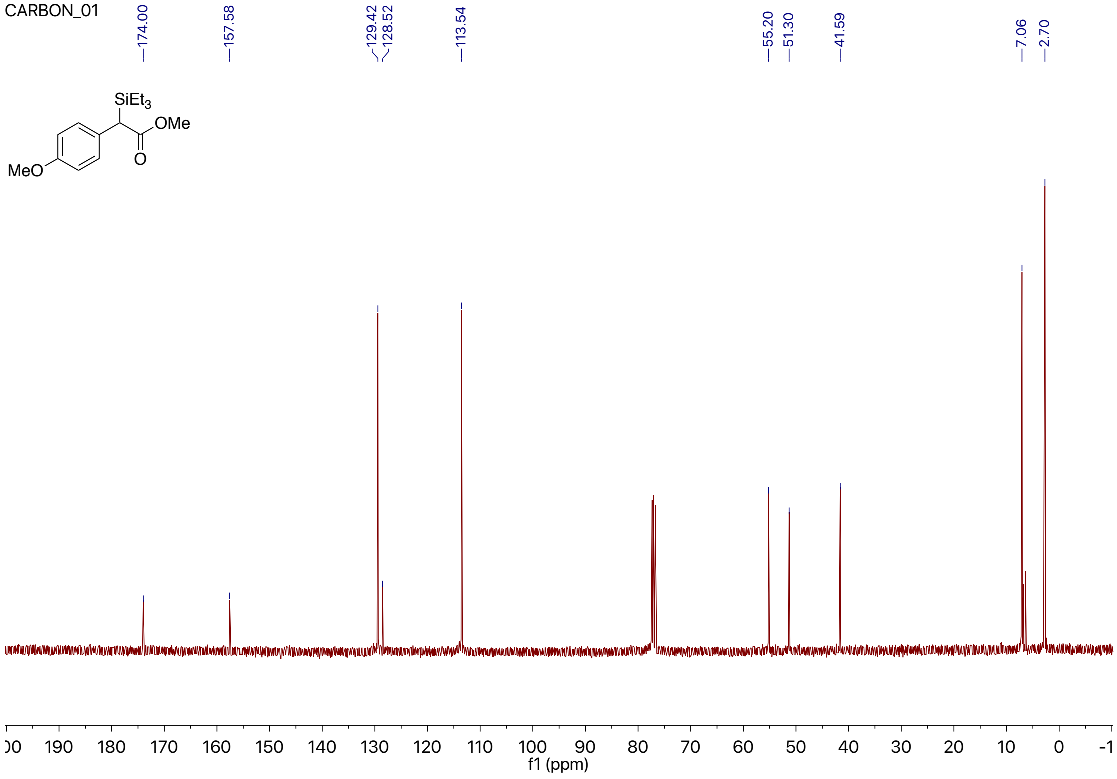

Methyl α-(*p*-fluorophenyl)-α-triethylsilylacetate (2d)

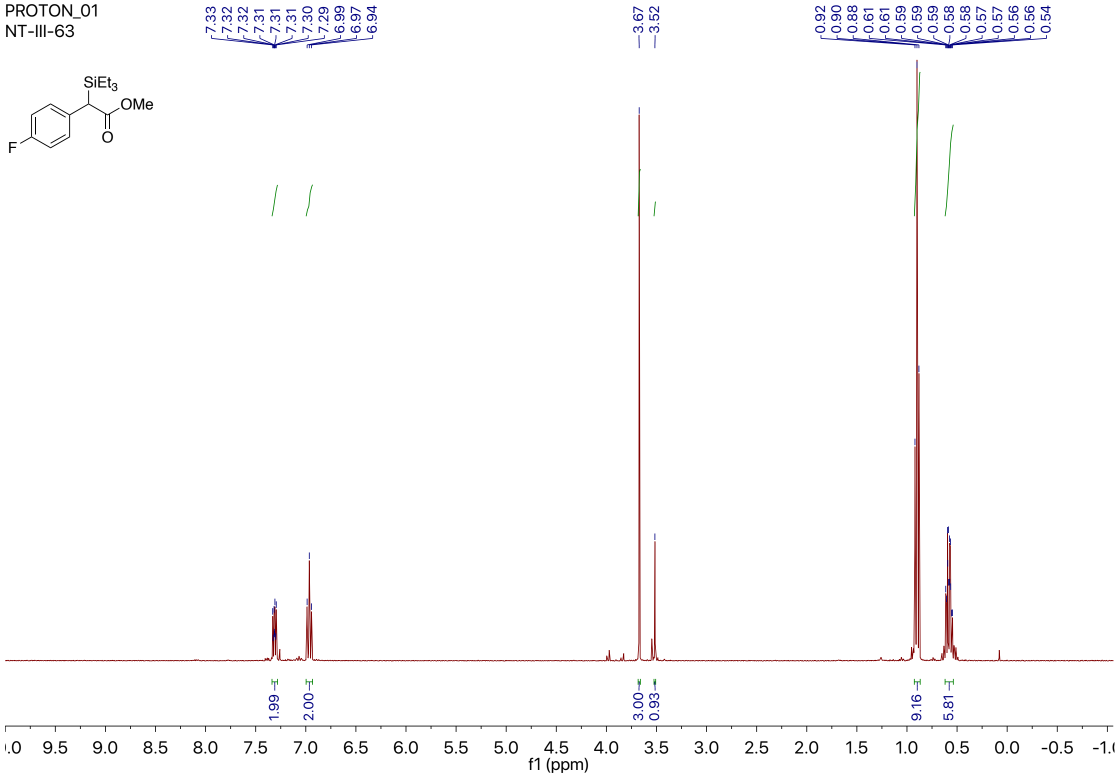

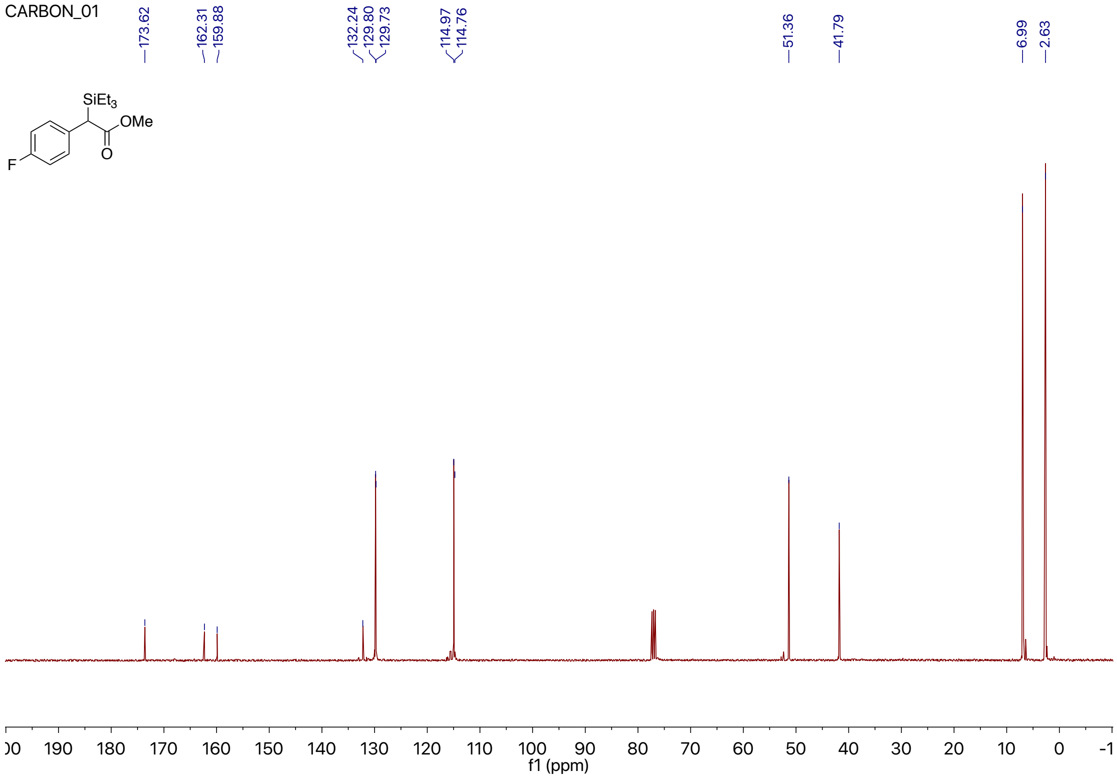

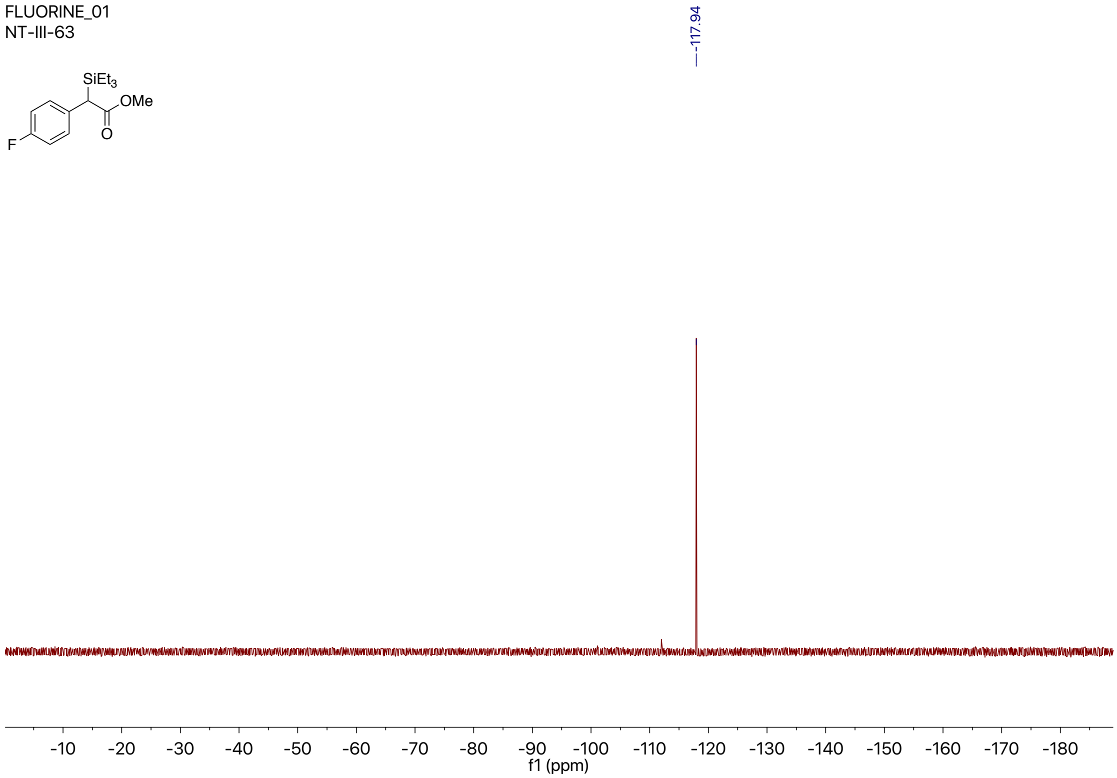

Methyl α-(*p*-chlorophenyl)-α-triethylsilylacetate (2e)

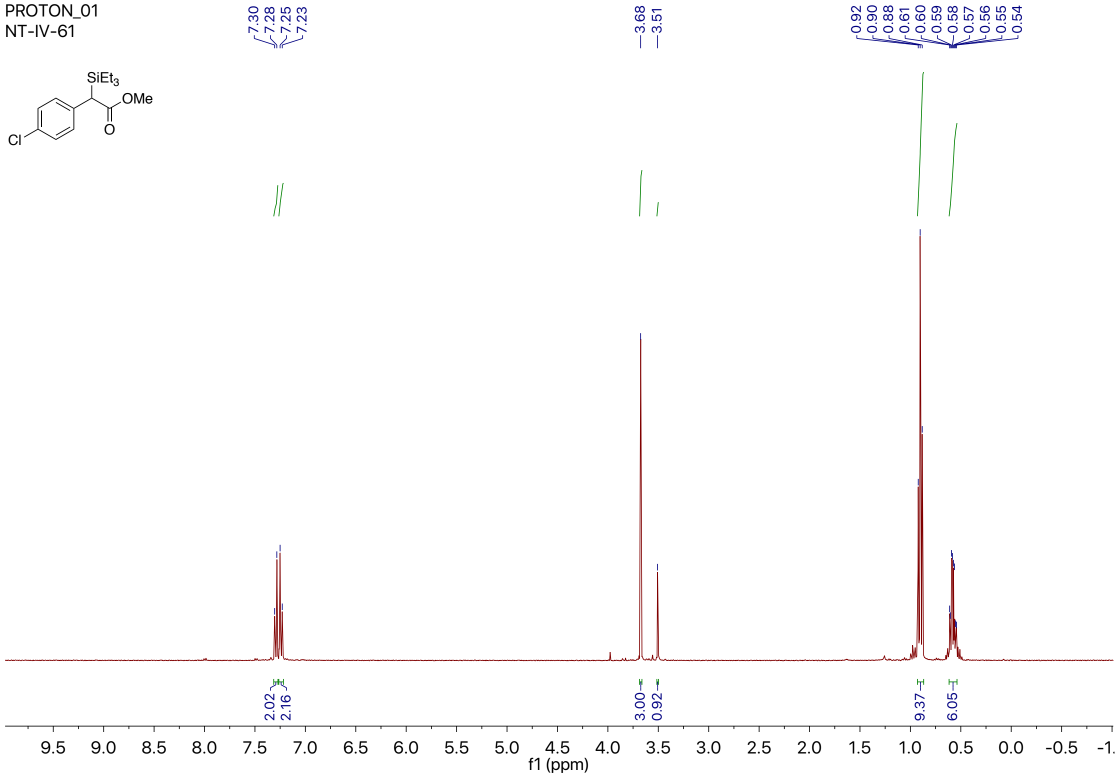

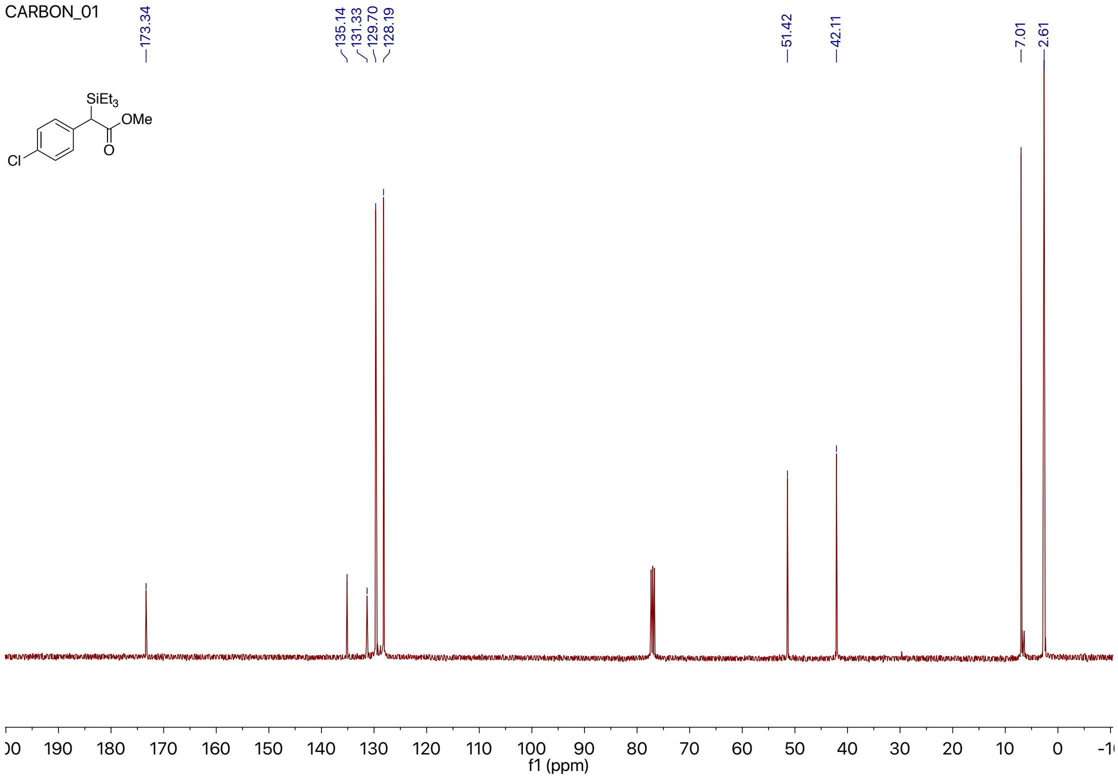

Methyl α-(*m*-chlorophenyl)-α-triethylsilylacetate (2f)

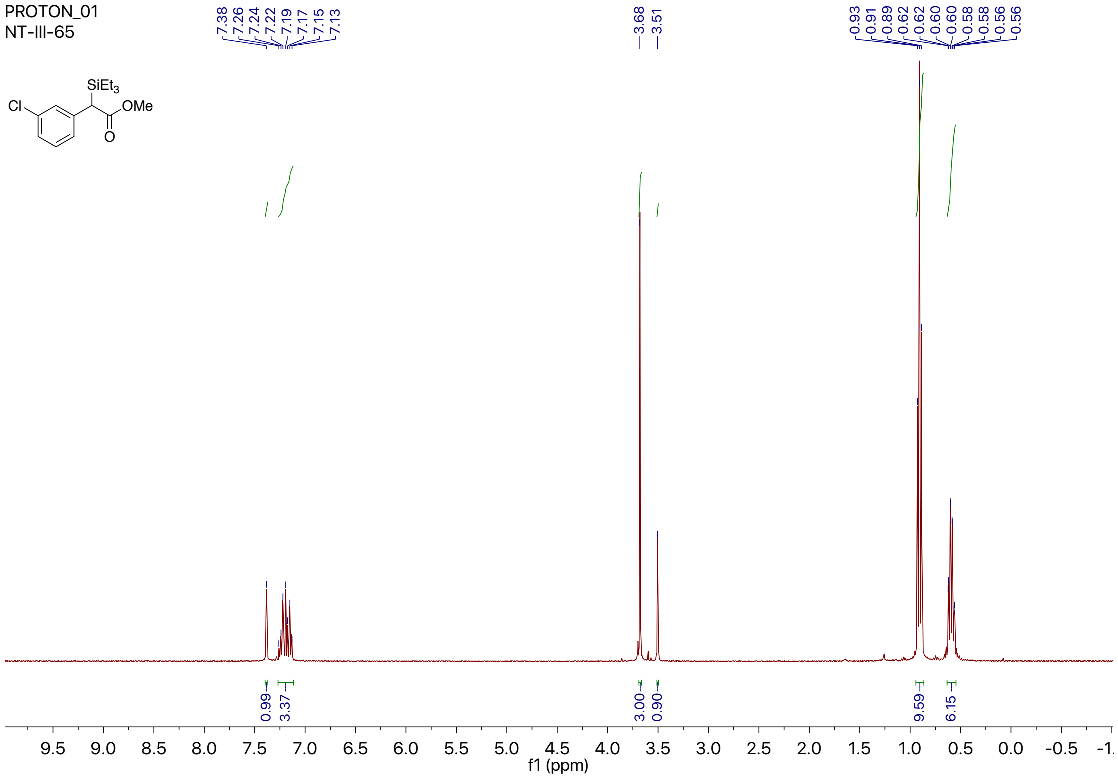

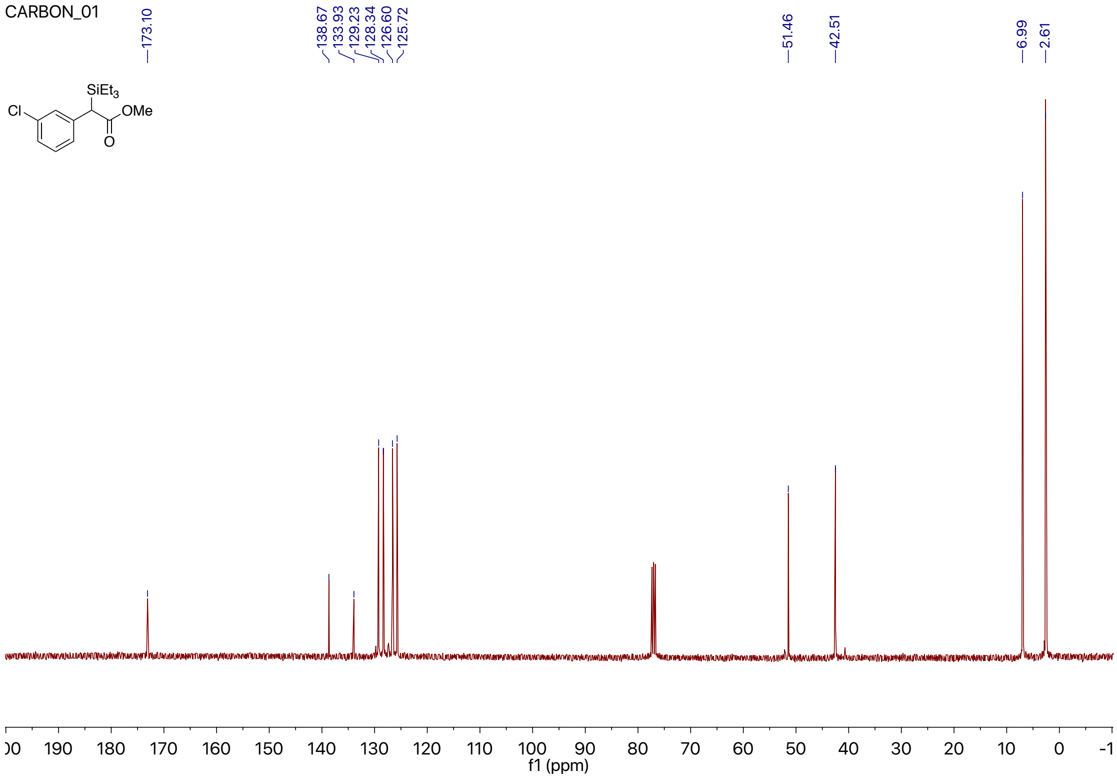

Methyl α-(*o*-chlorophenyl)-α-triethylsilylacetate (2g)

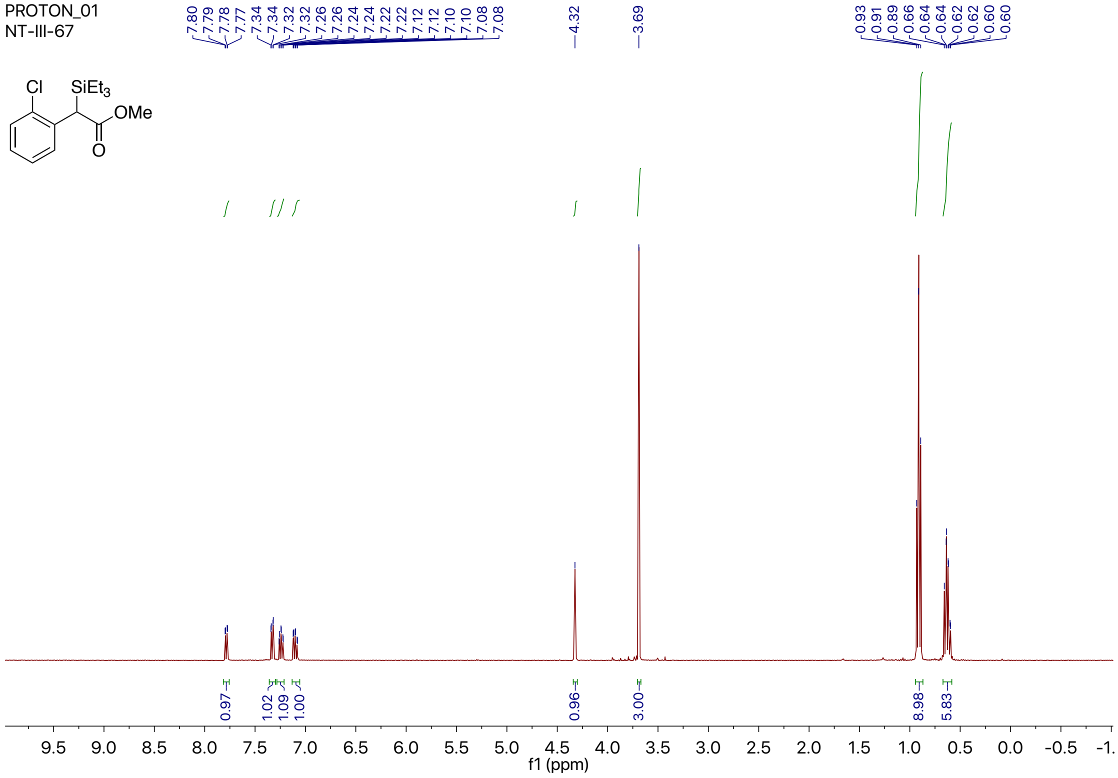

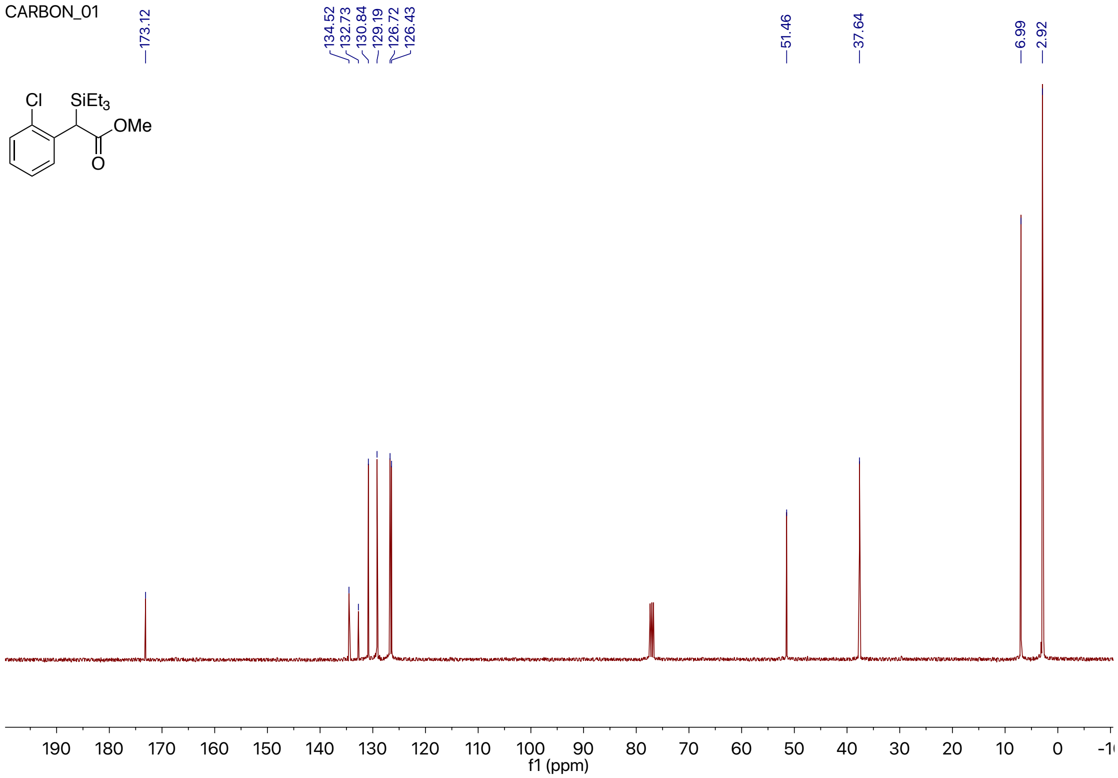

Methyl α-(*p*-bromophenyl)-α-triethylsilylacetate (2h)

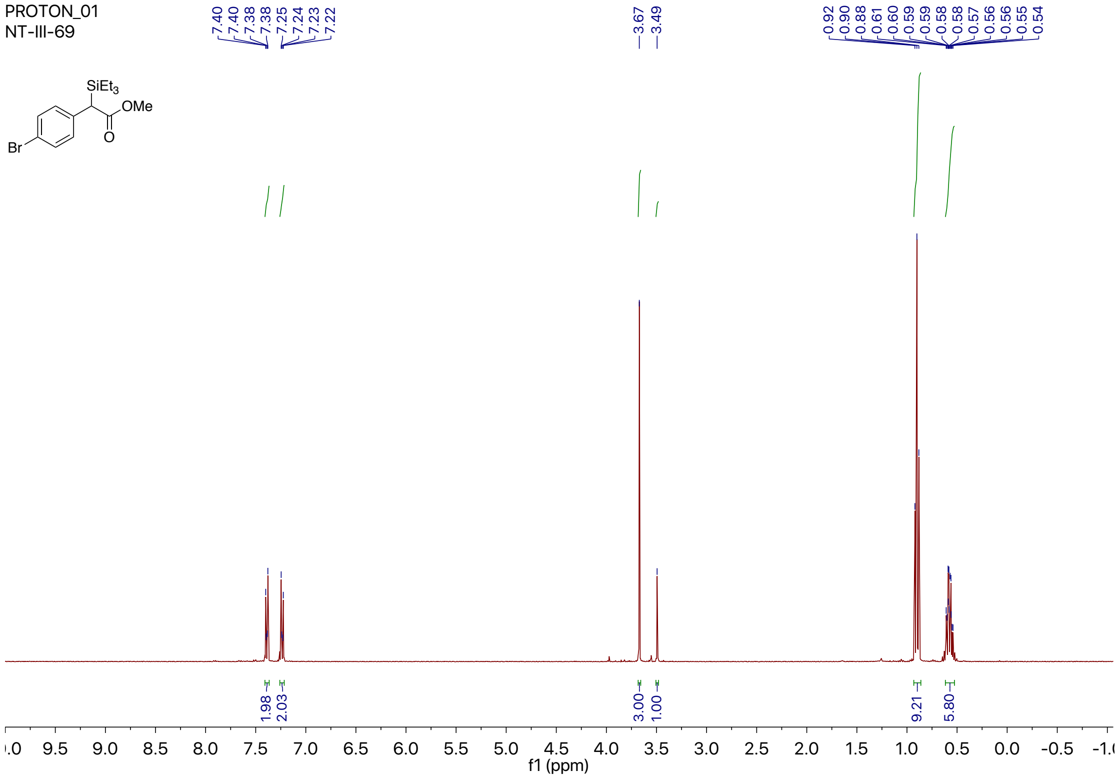

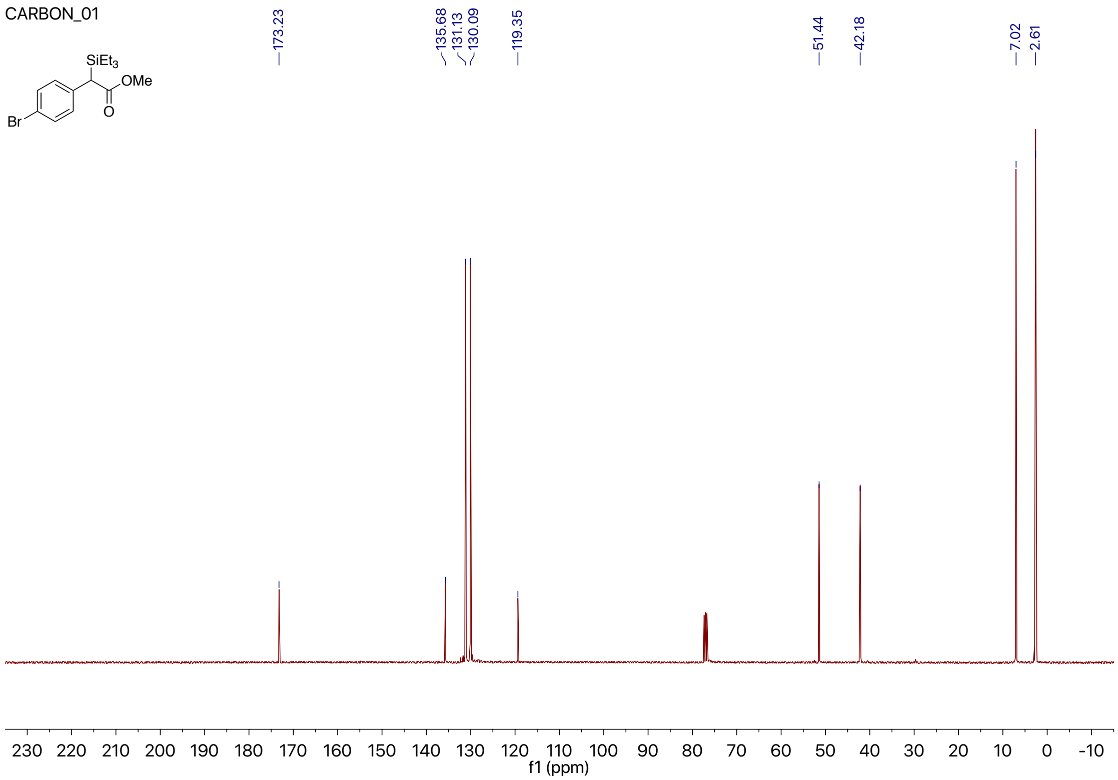

Ethyl α-phenyl-α-triethylsilylacetate (2i)

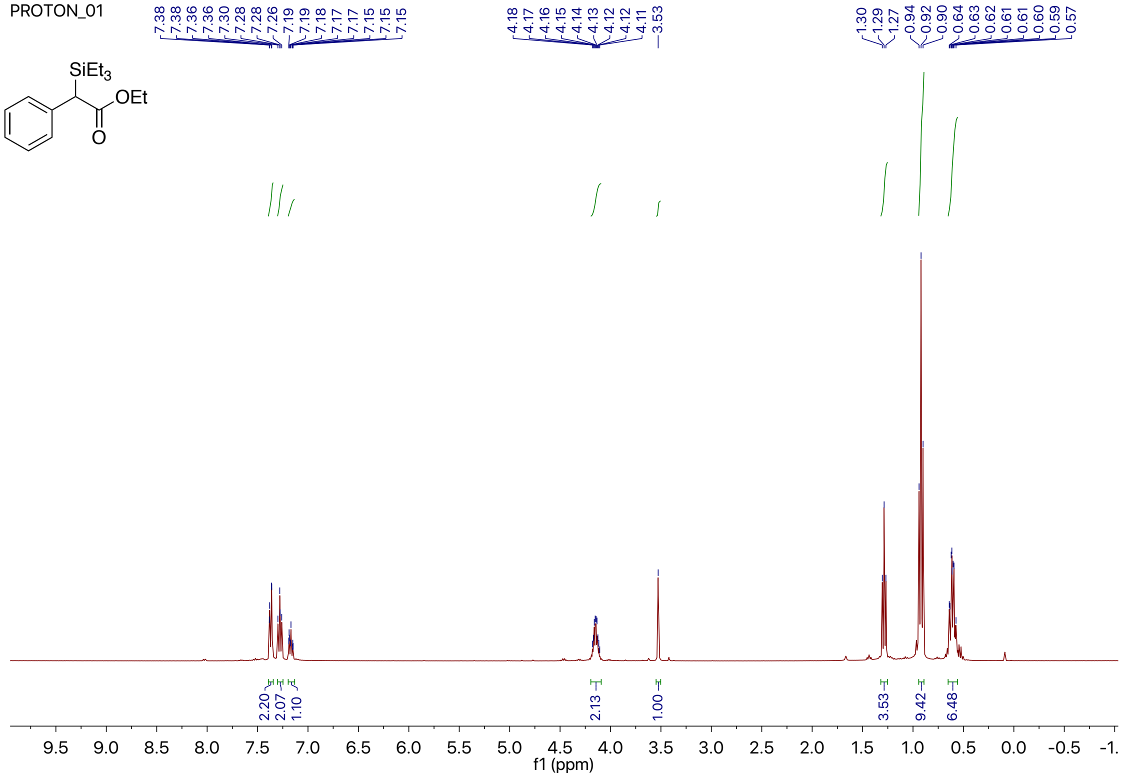

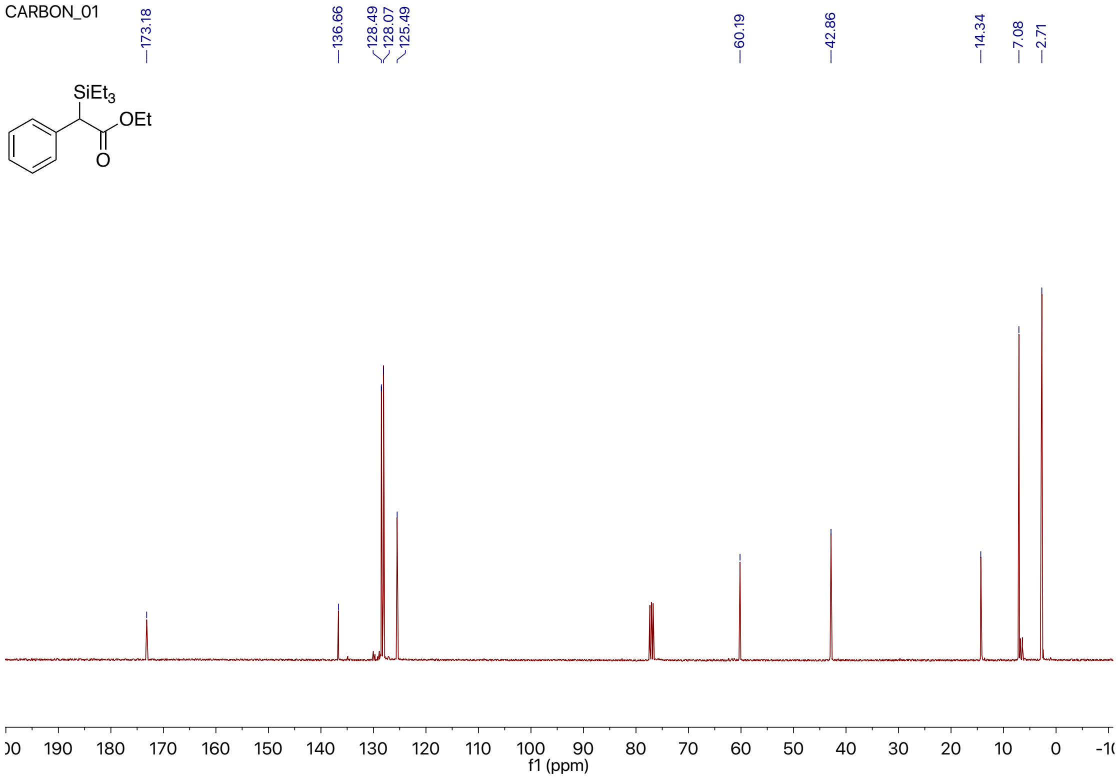

tert-Butyl α-phenyl-α-triethylsilylacetate (2j)

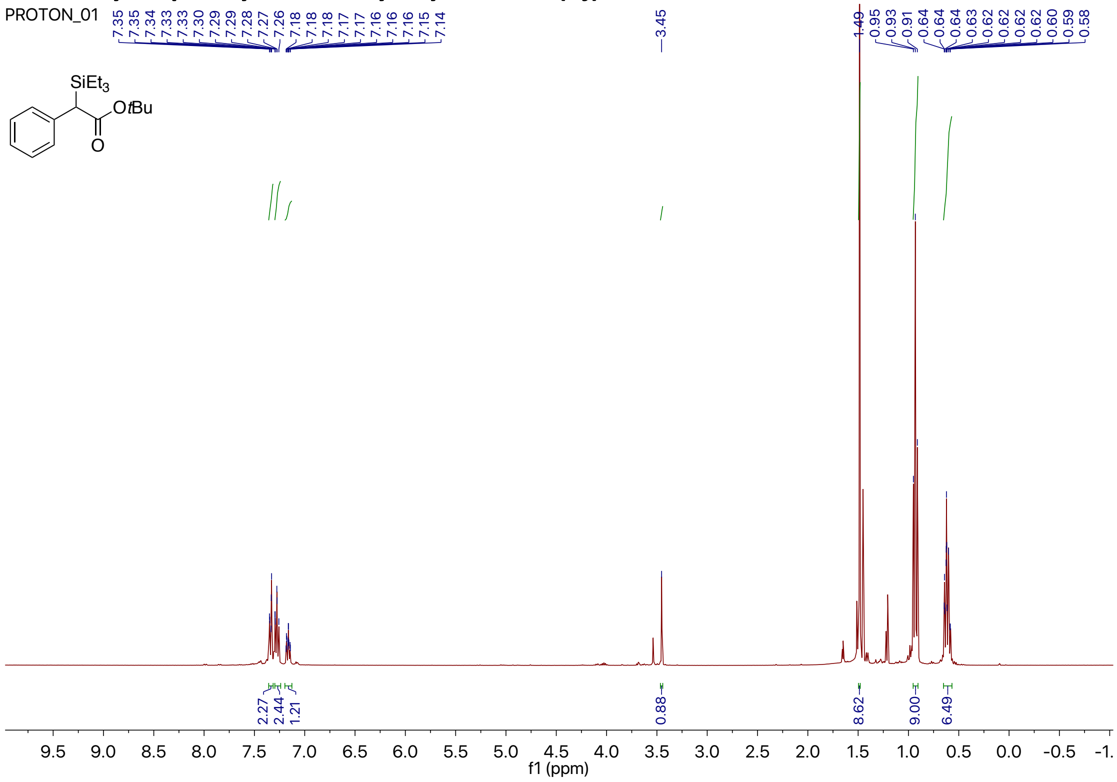

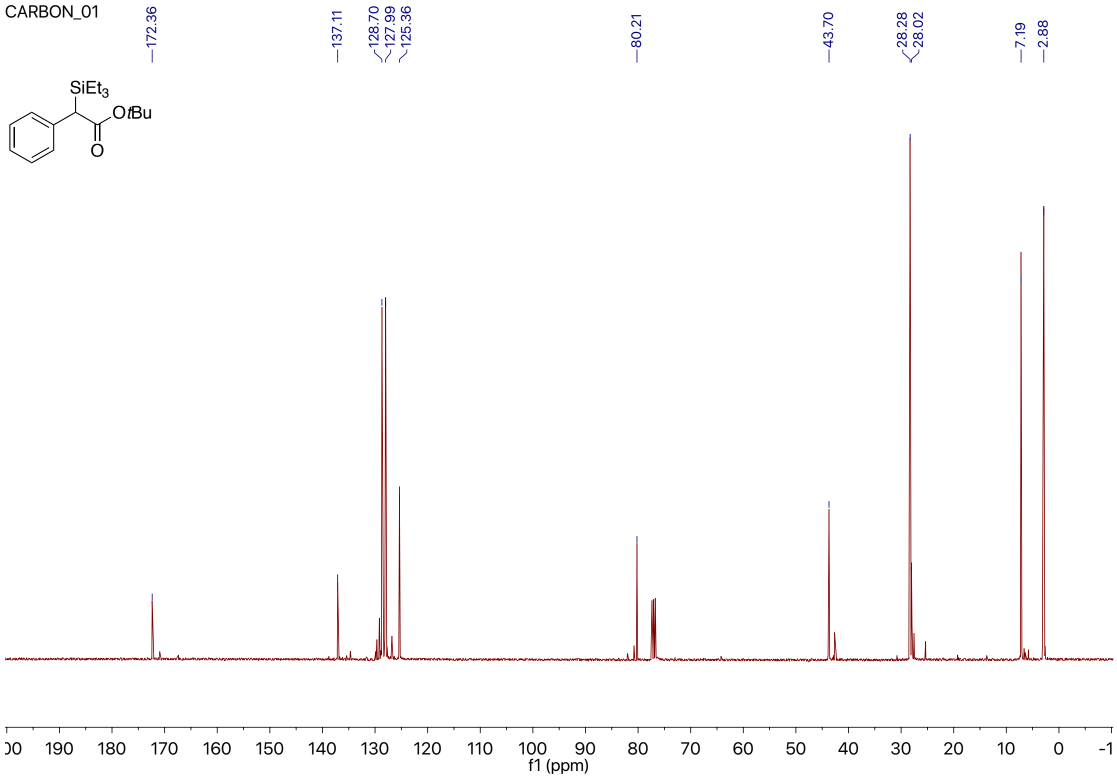

Diethyl (phenyl(triethylsilyl)methyl)phosphonate (2k)

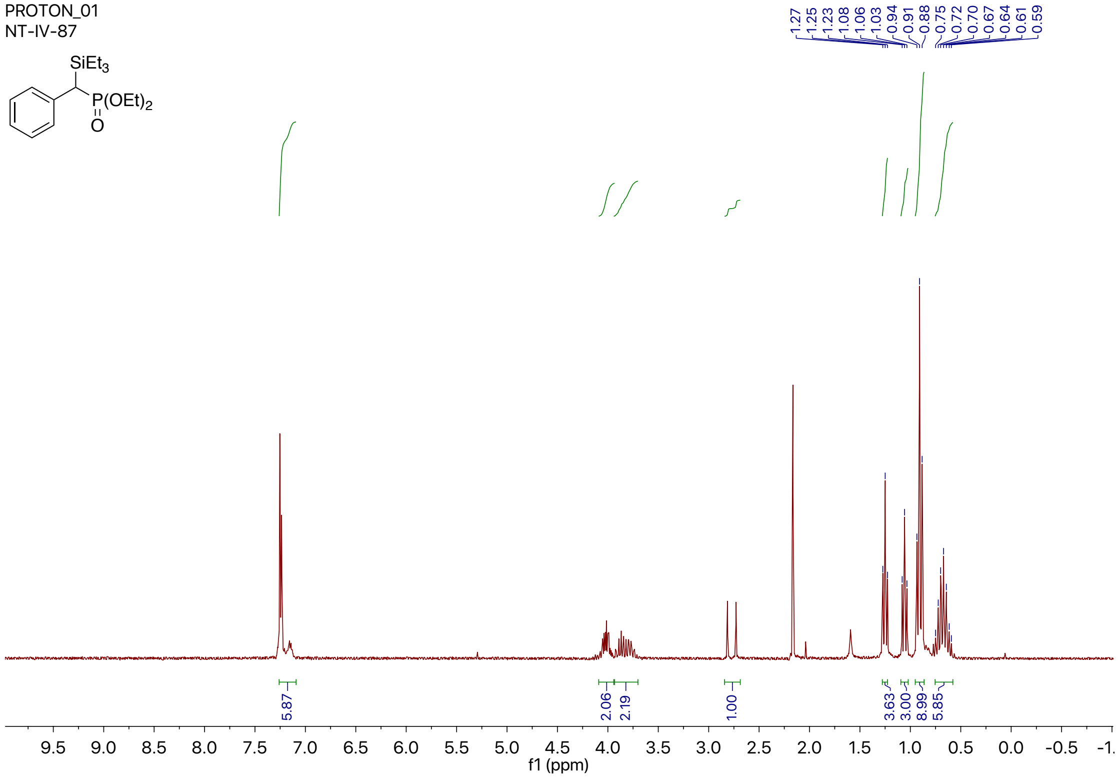

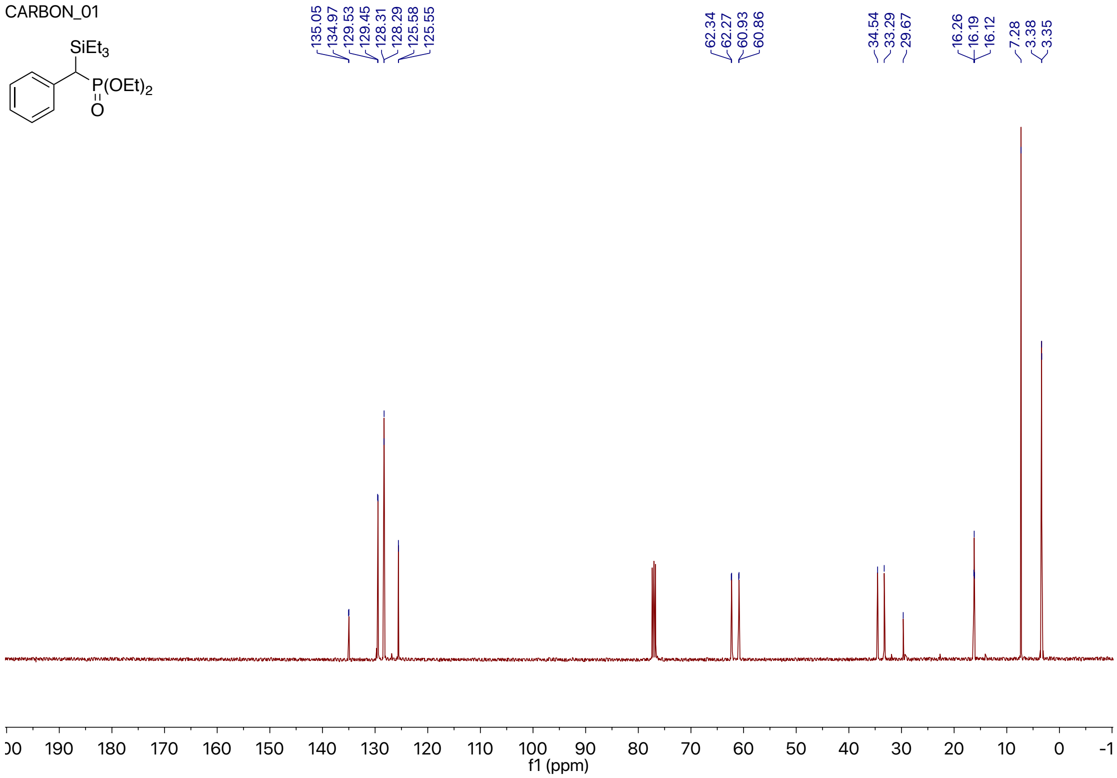

(1-([1,1'-Biphenyl]-4-yl)-2,2,2-trifluoroethyl)triethylsilane (2l)

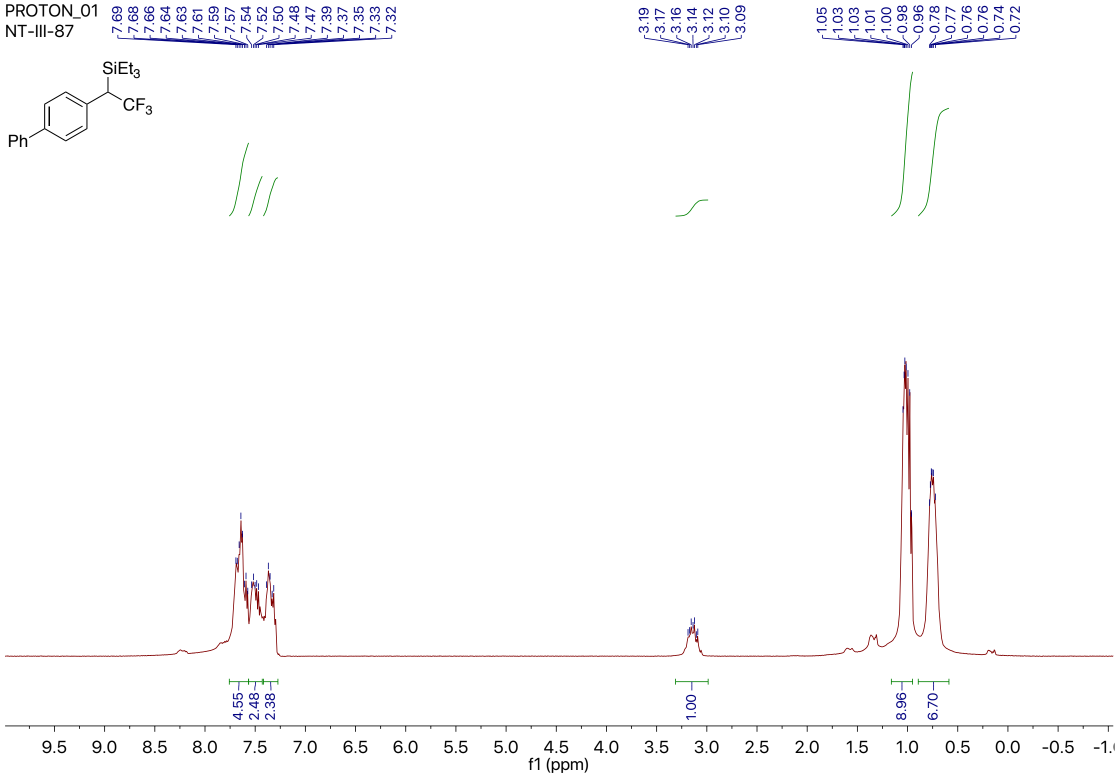

CARBON_01

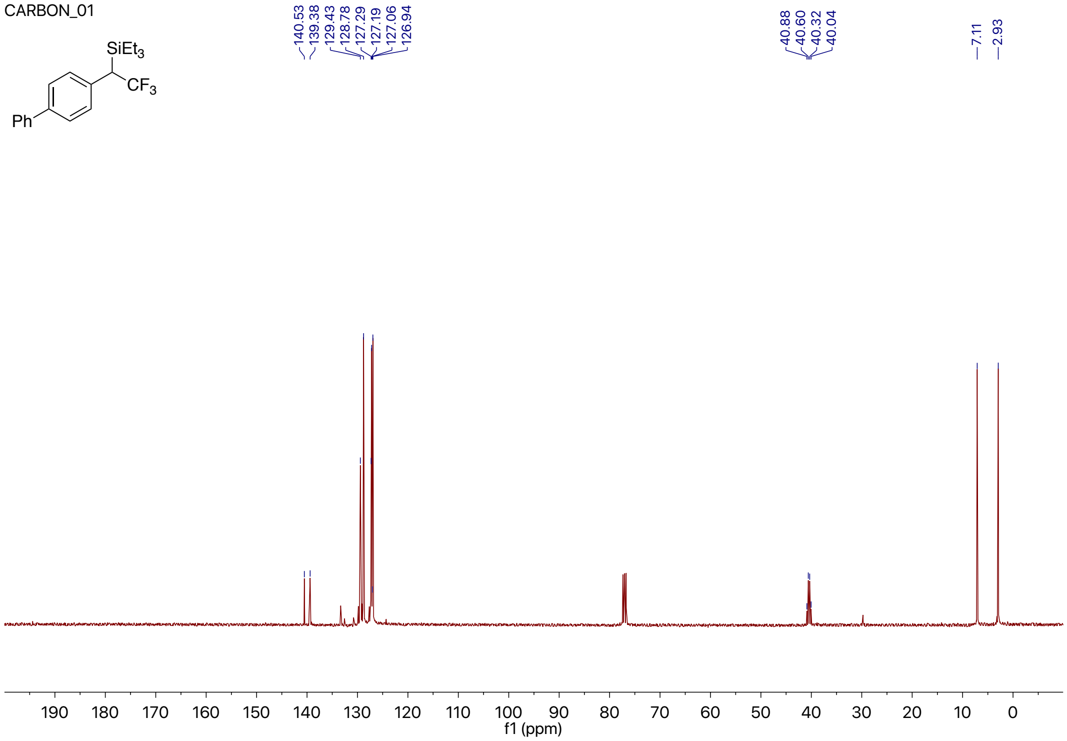

FLUORINE_01

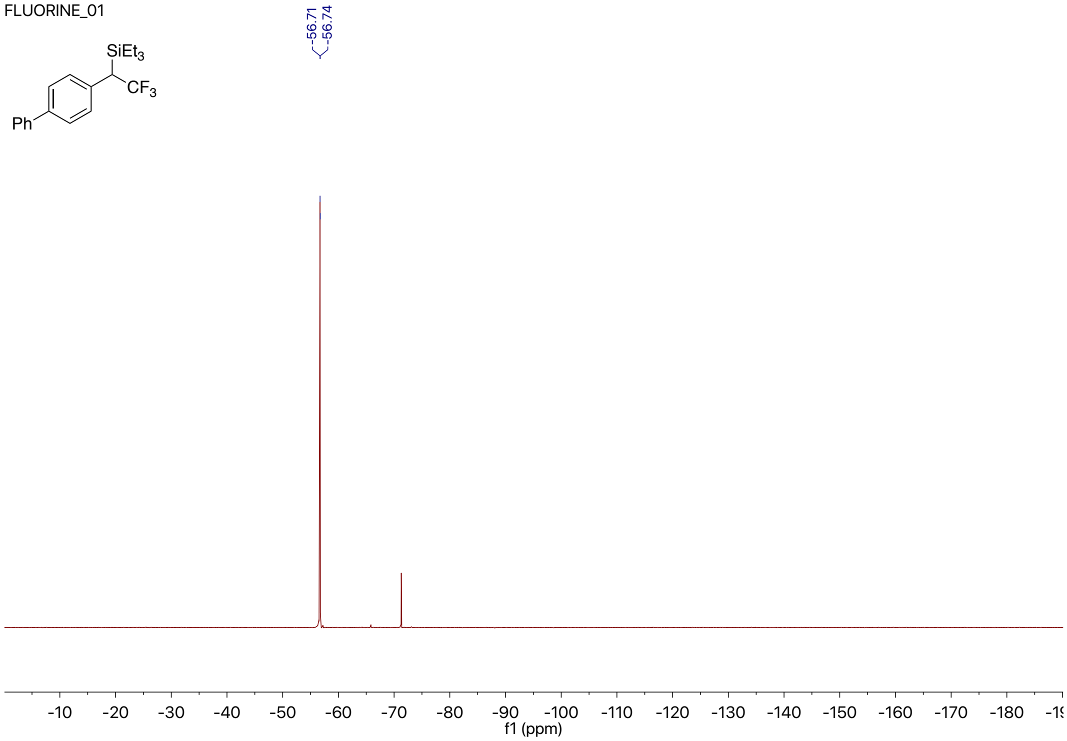

(1-(4-Bromophenyl)-2,2,2-trifluoroethyl)triethylsilane (2m)

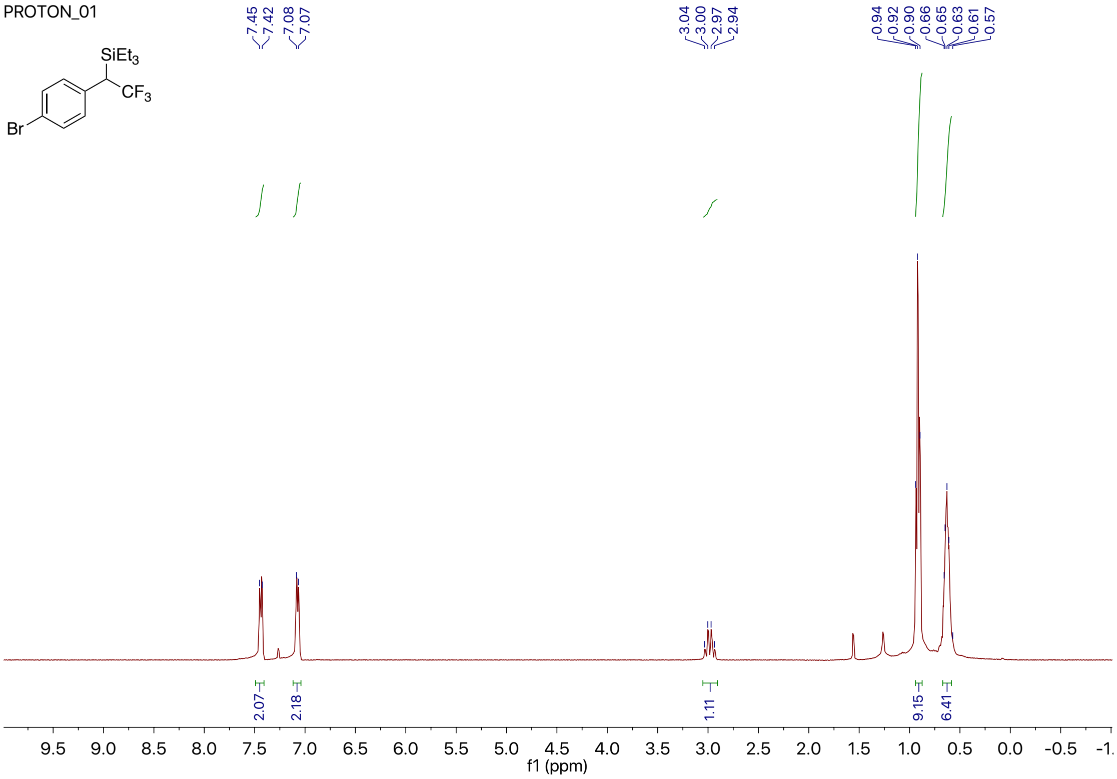

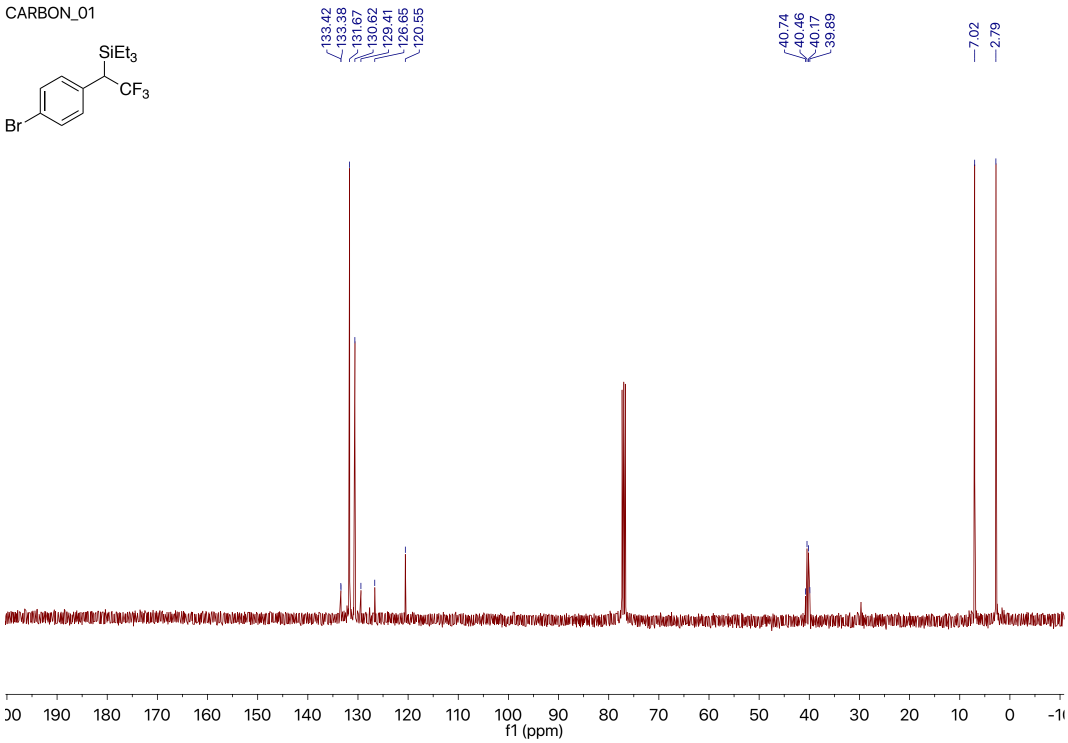

FLUORINE_01

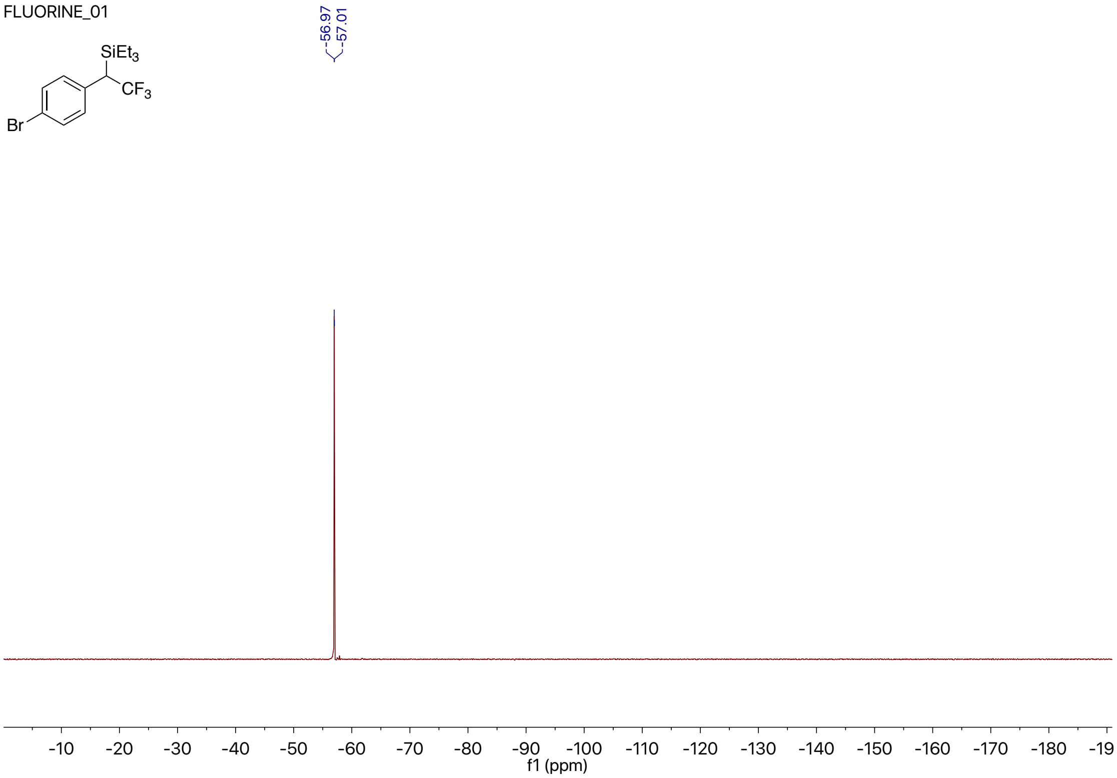

Methyl α-phenyl-α-dimethyl(phenyl)silylacetate (2n)

PROTON_01
NT-III-3

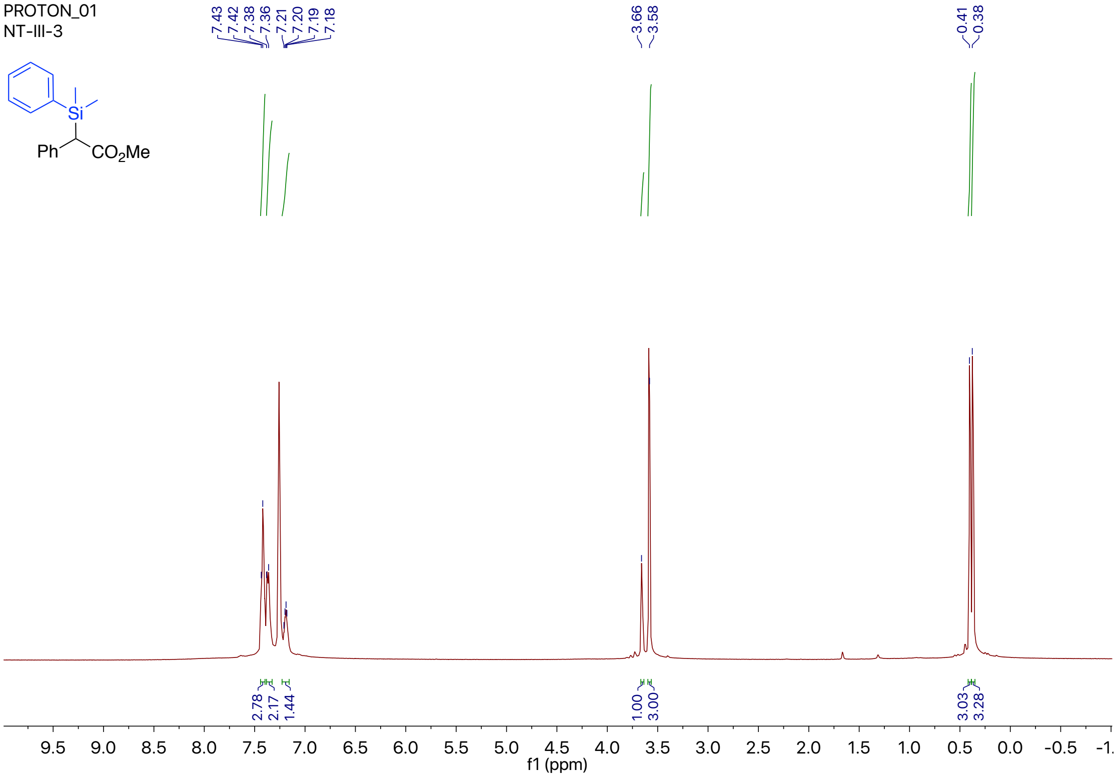

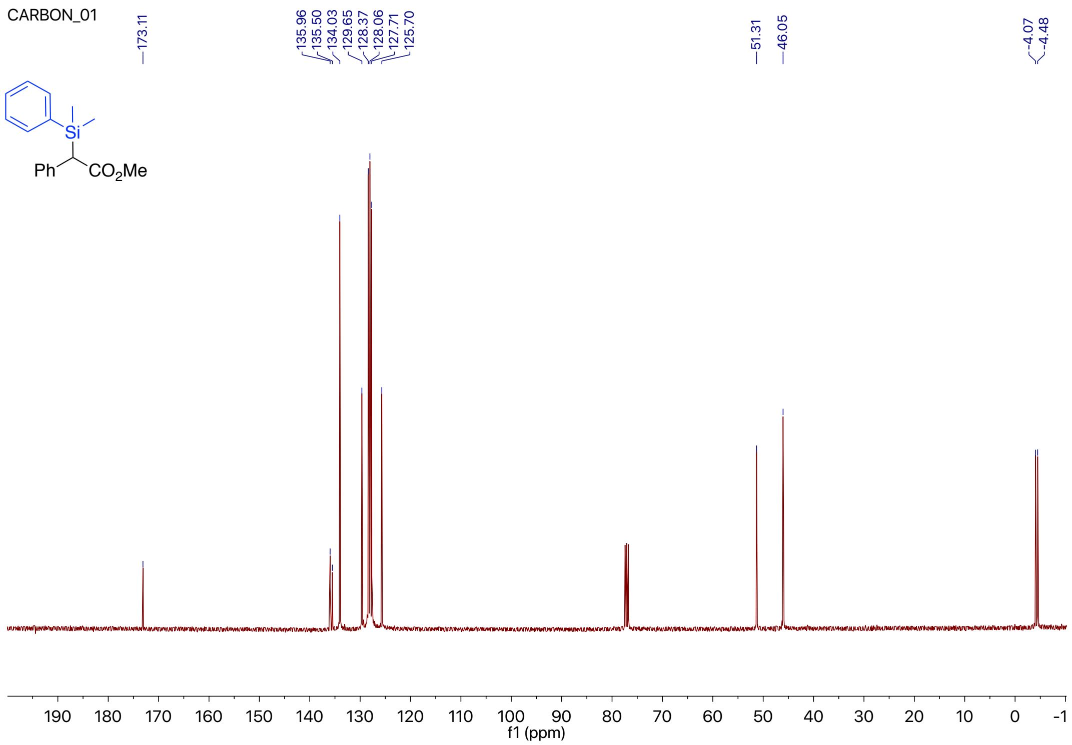

Methyl α-phenyl-α-dimethyl(2-naphthyl)silylacetate (2o)

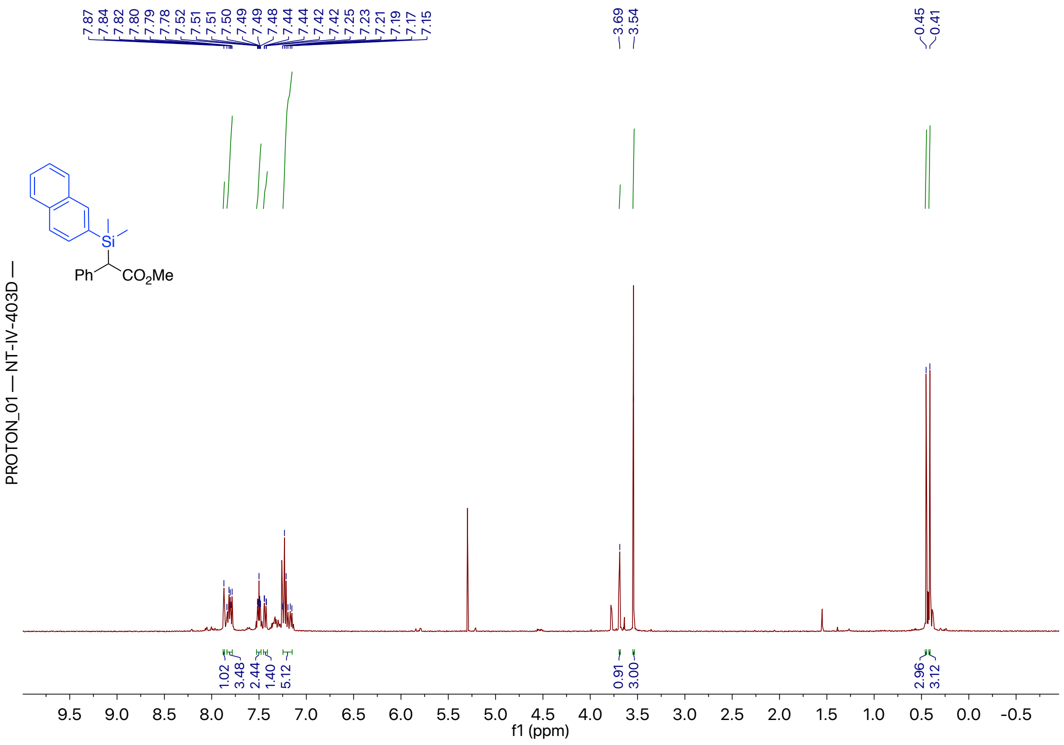

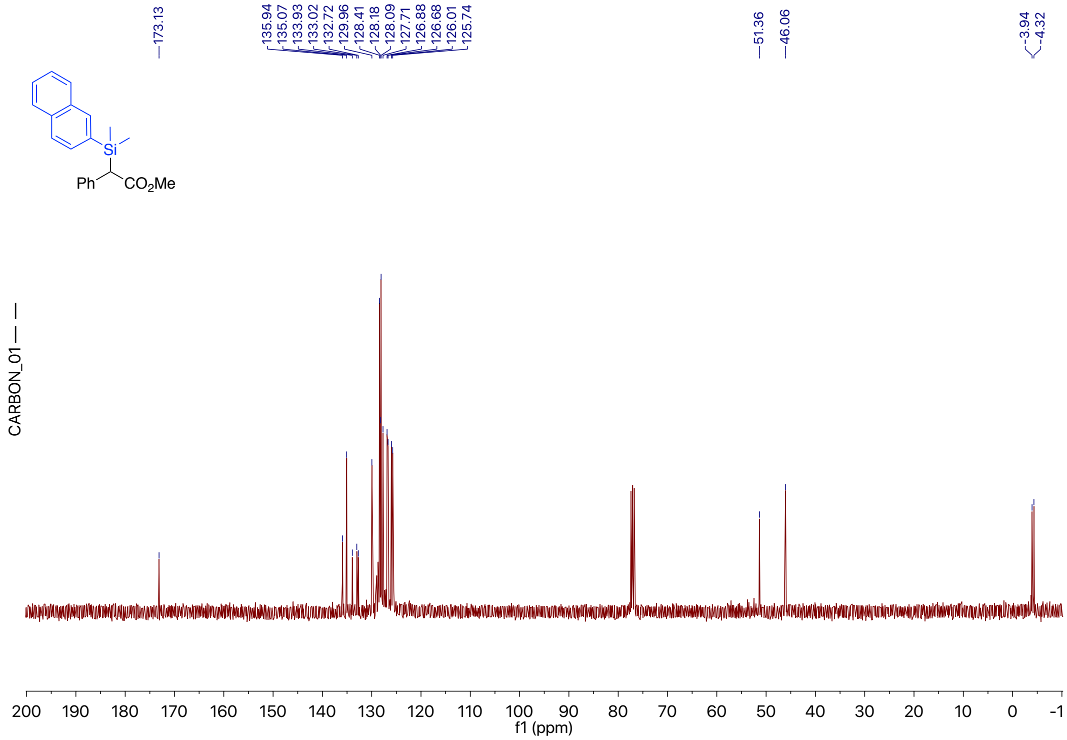

Methyl α-phenyl-α-dimethyl(*o,o'*-difluorophenyl)silylacetate (2q)

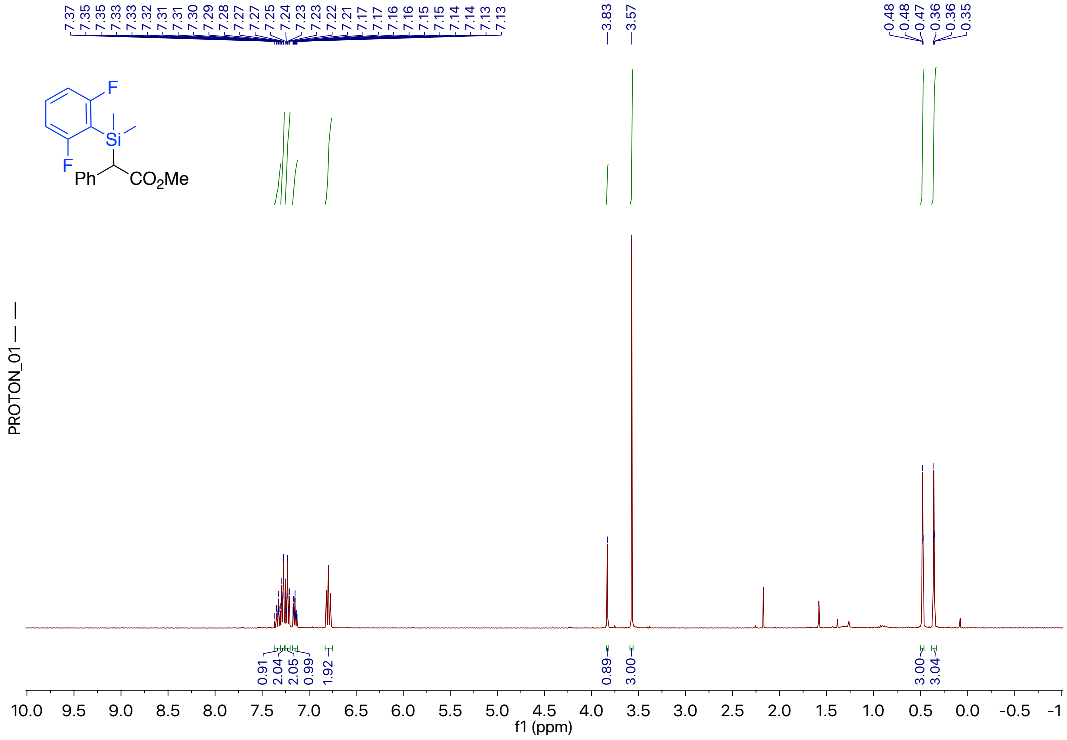

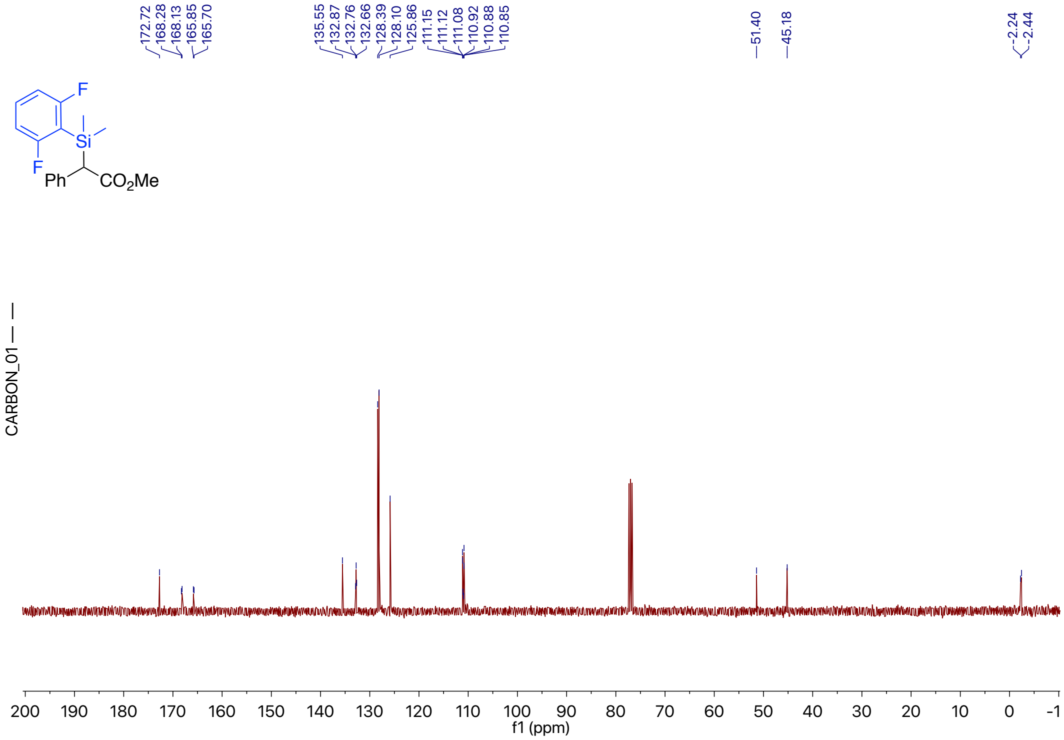
172.72
168.28
168.13
165.85
165.70
135.55
132.87
132.76
132.66
128.39
128.10
125.86
111.15
111.12
111.08
110.92
110.88
110.85
51.40
45.18
-2.24
-2.44
F
Si
F
Ph
CO2Me
CARBON_01
200 190 180 170 160 150 140 130 120 110 100 90 80 70 60 50 40 30 20 10 0 -1
f1 (ppm)

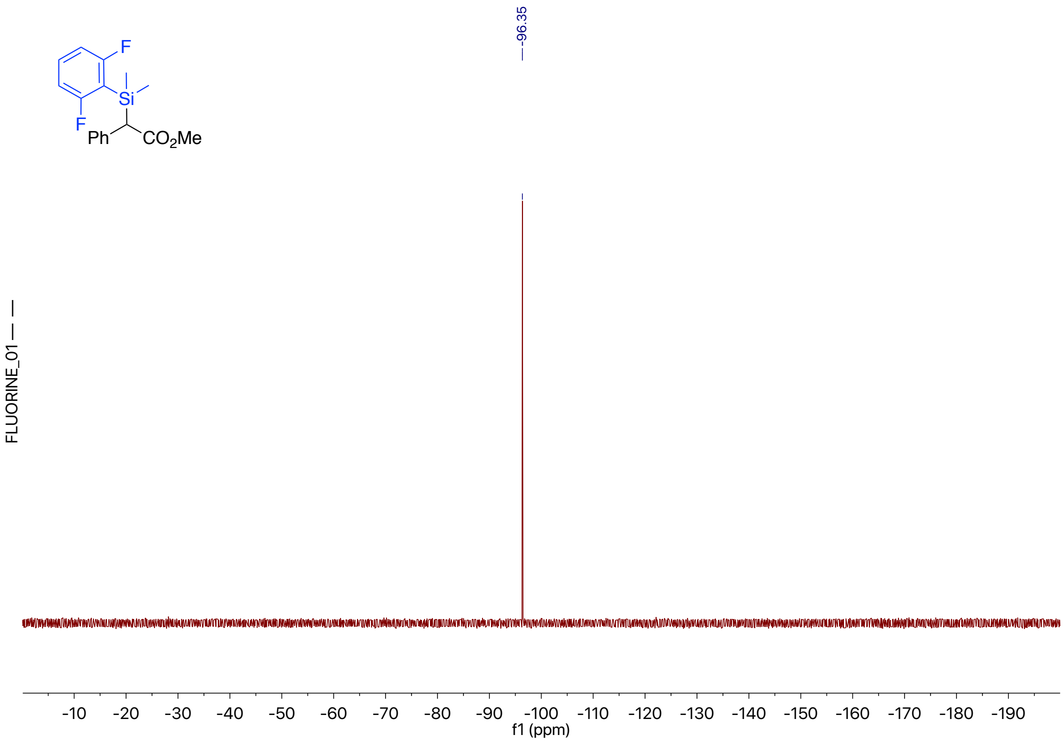
-96.35
F
Si
F
Ph
CO2Me
FLUORINE_01
-10 -20 -30 -40 -50 -60 -70 -80 -90 -100 -110 -120 -130 -140 -150 -160 -170 -180 -190
f1 (ppm)

Methyl α-phenyl-α-dimethyl(*m,m'*-di(trifluoromethyl)phenyl)silylacetate (2r)

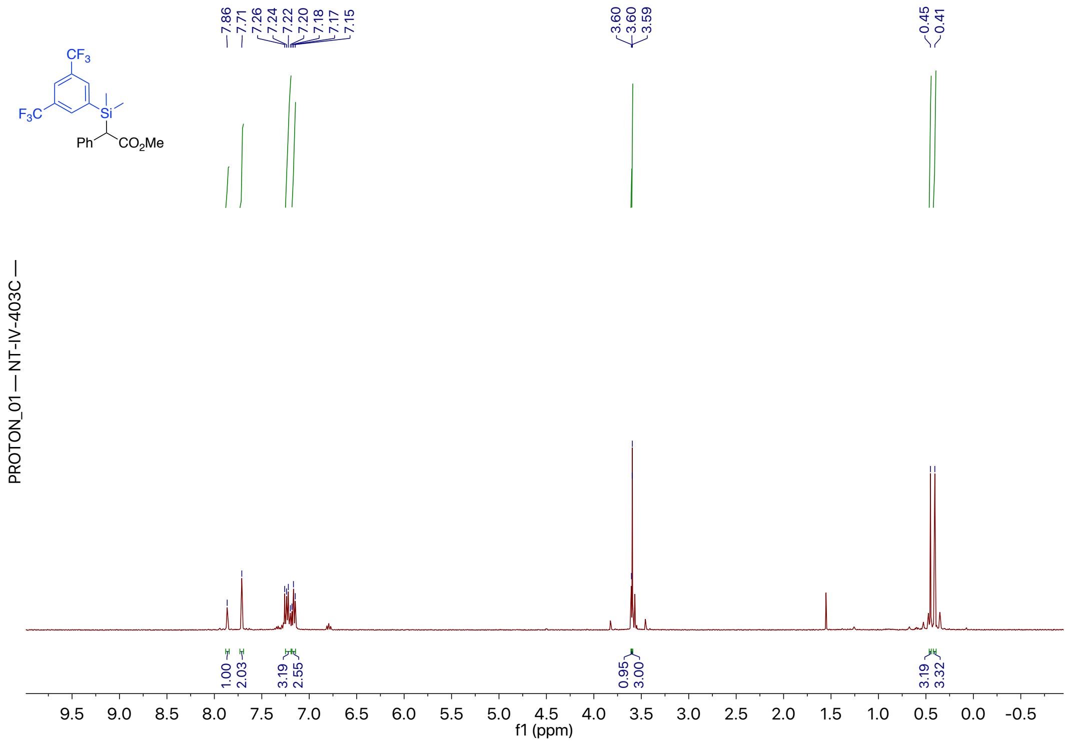

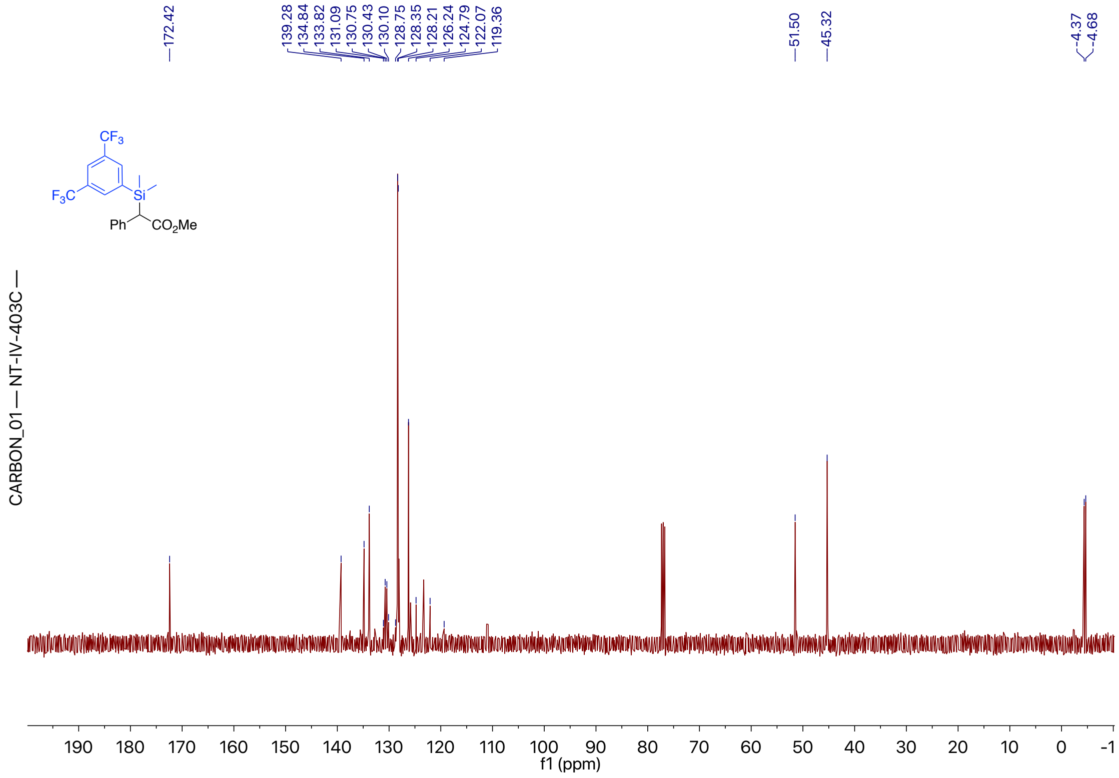

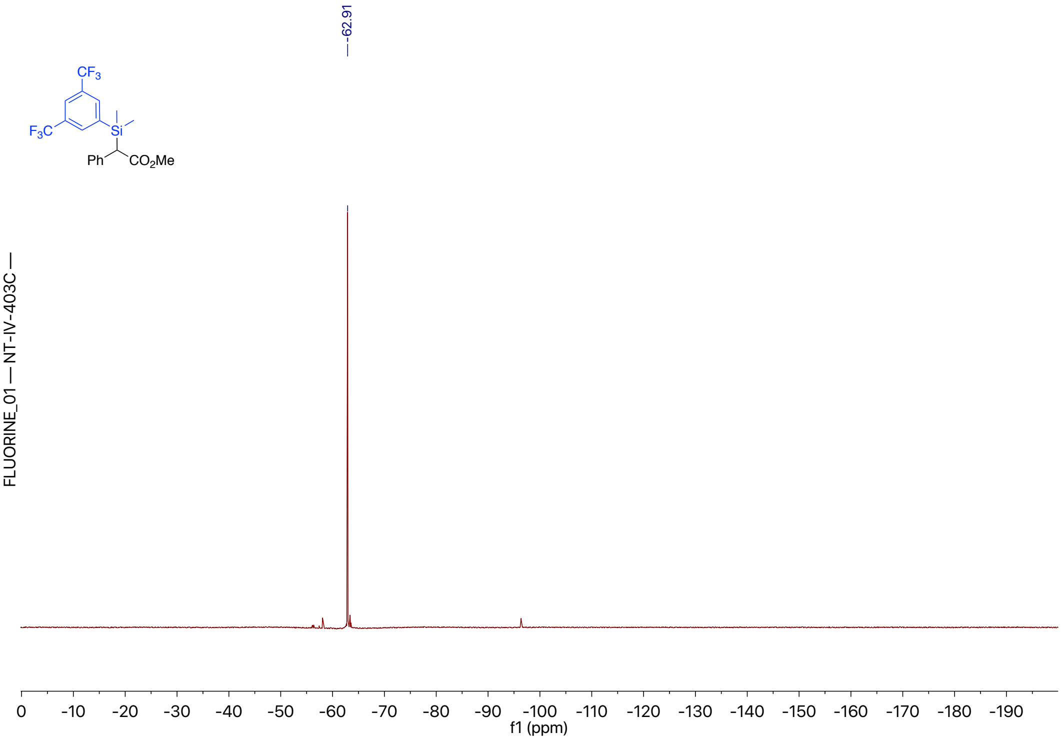

Methyl α-phenyl-α-methyldiphenylsilylacetate (2s)

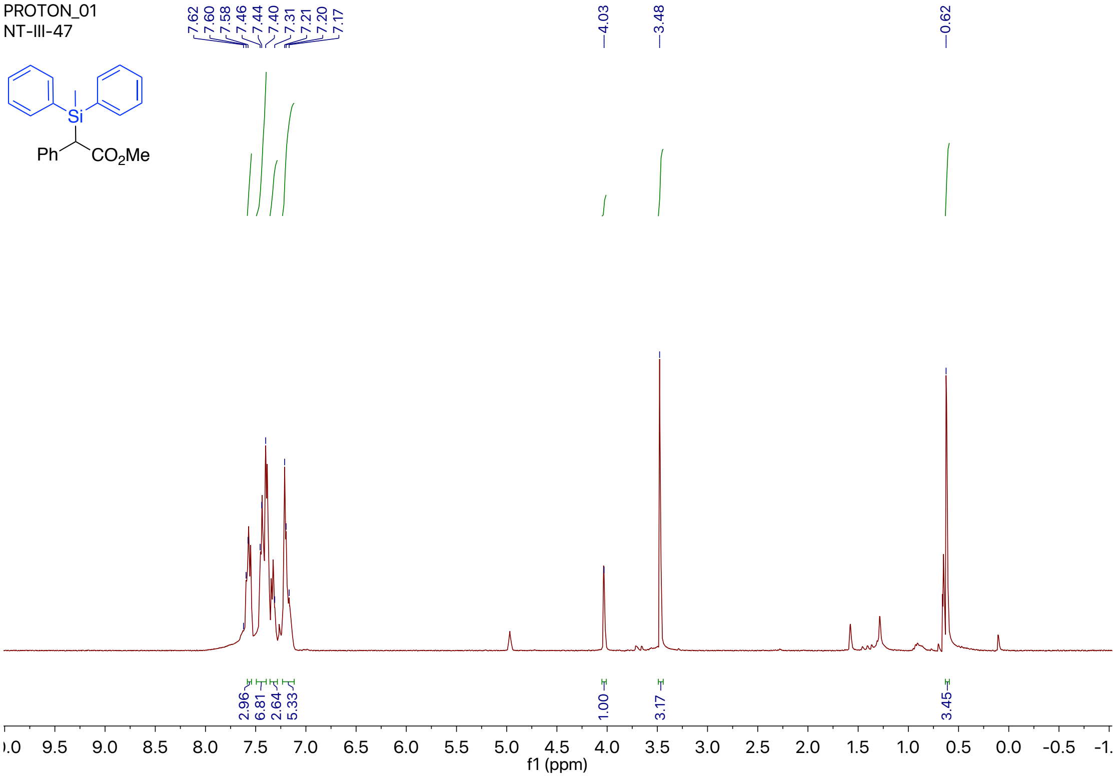

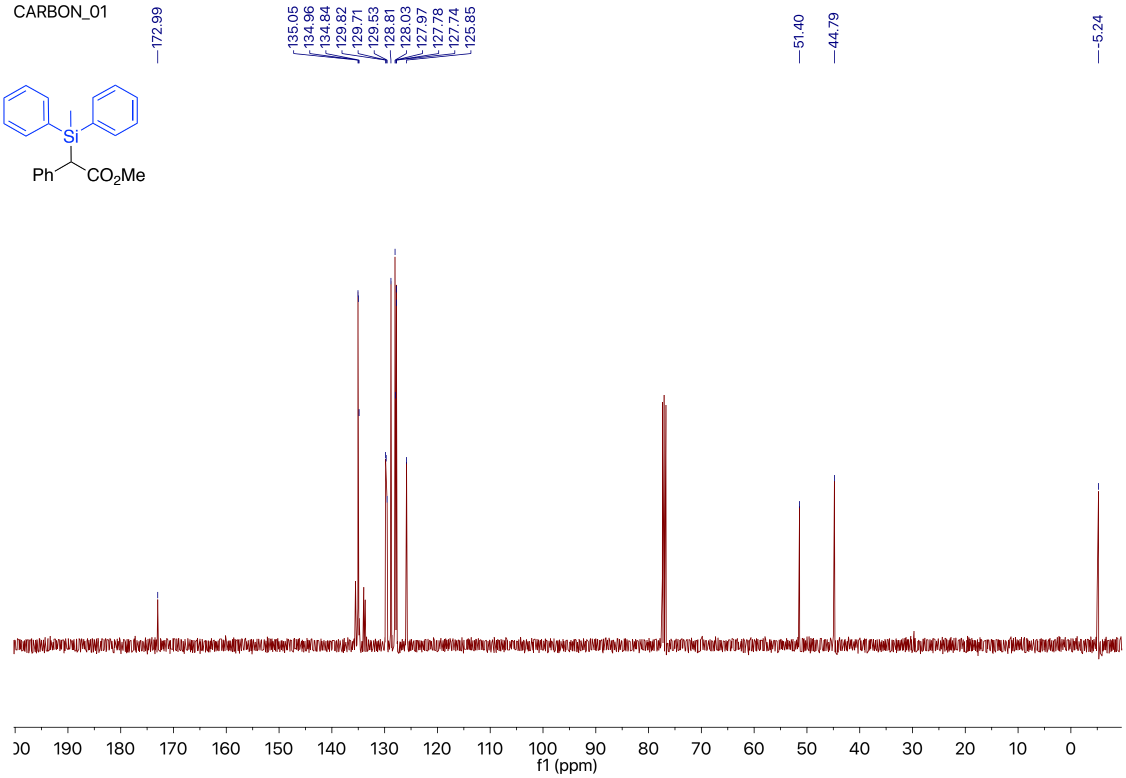

Methyl α-phenyl-α-*tert*-butyldimethylsilylacetate (2u)

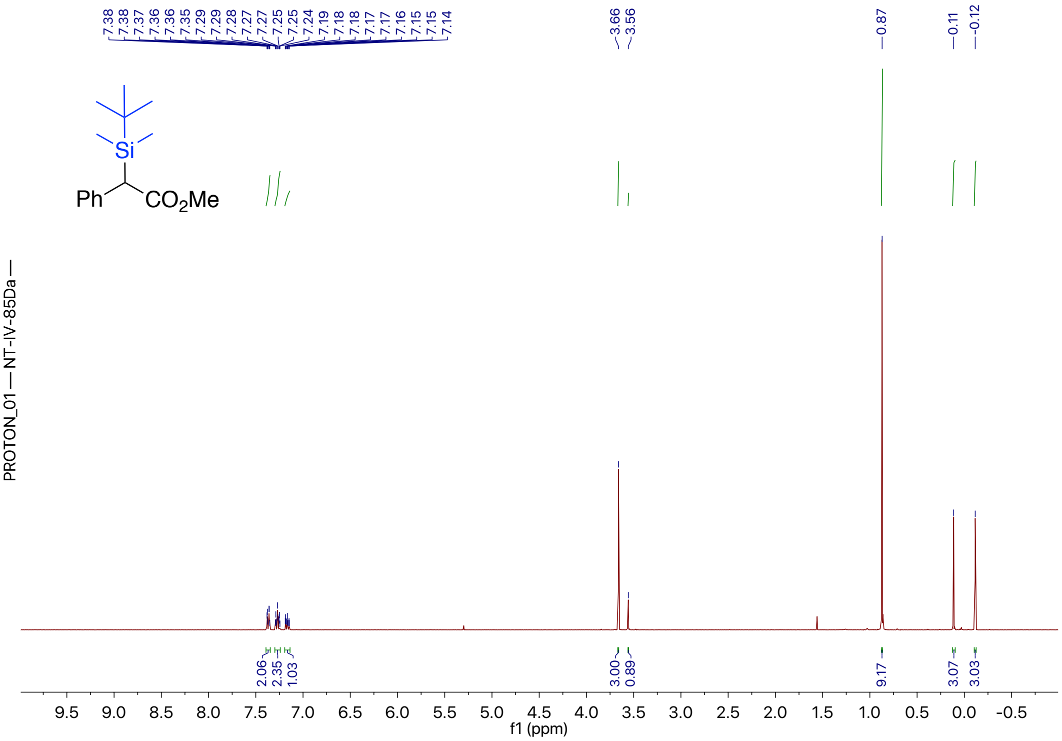

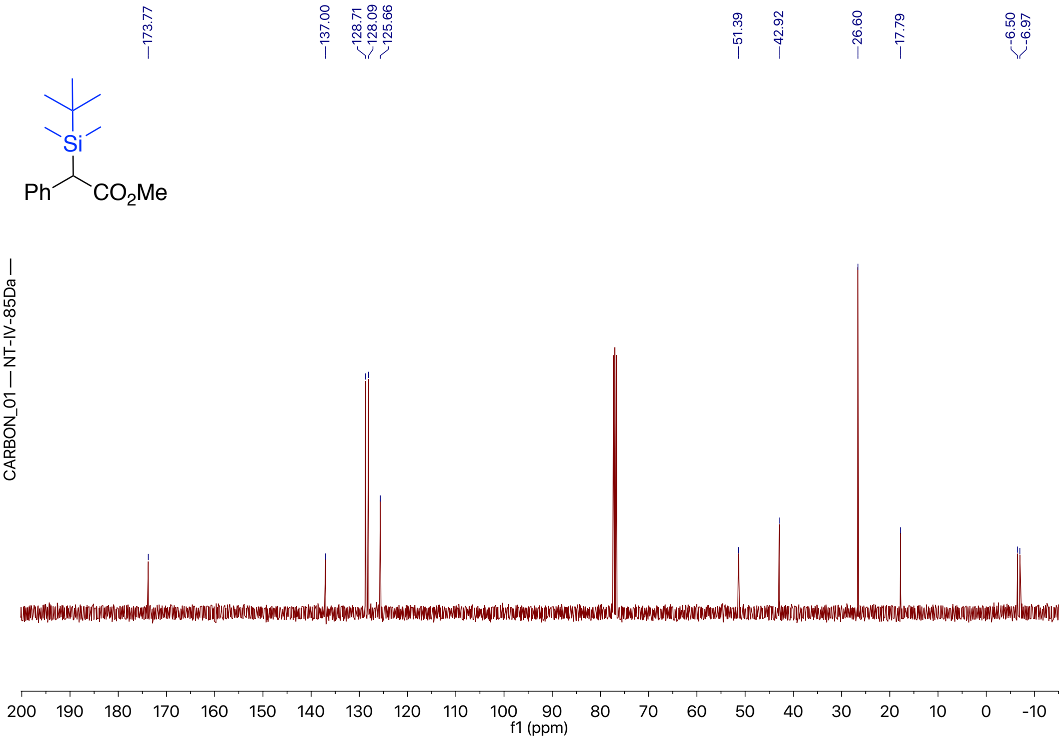

Methyl α-phenyl-α-tri-*n*-hexylsilylacetate (2v)

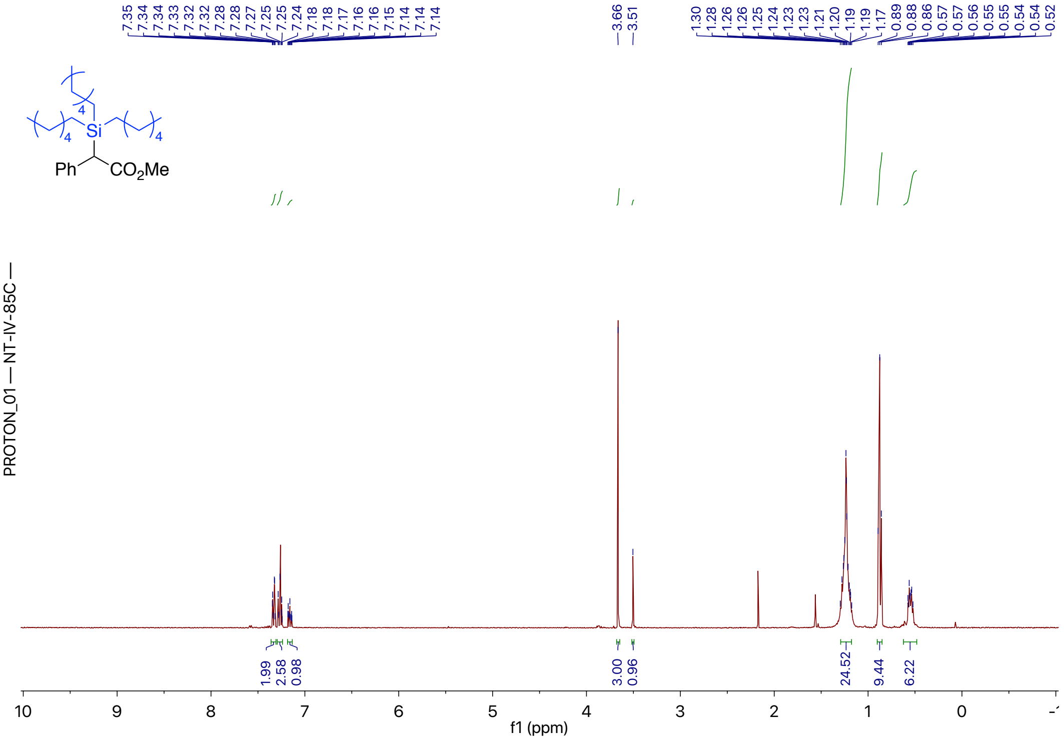

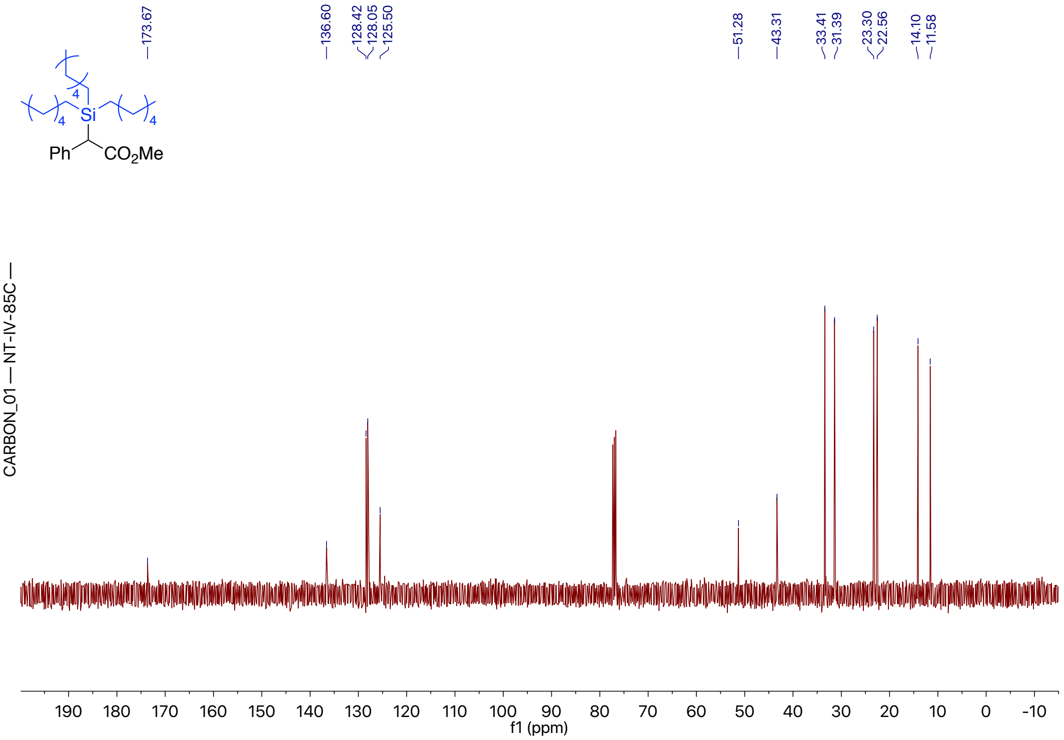

Methyl 2-hydroxy-2-phenylacetate (3a)

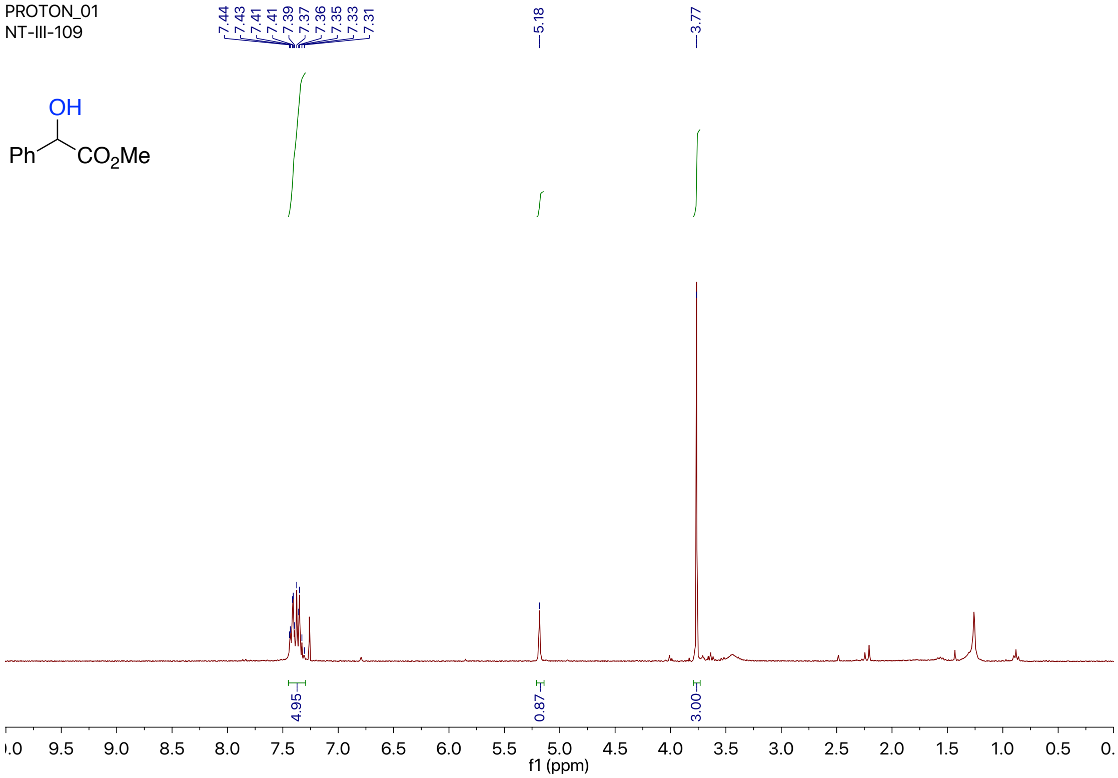

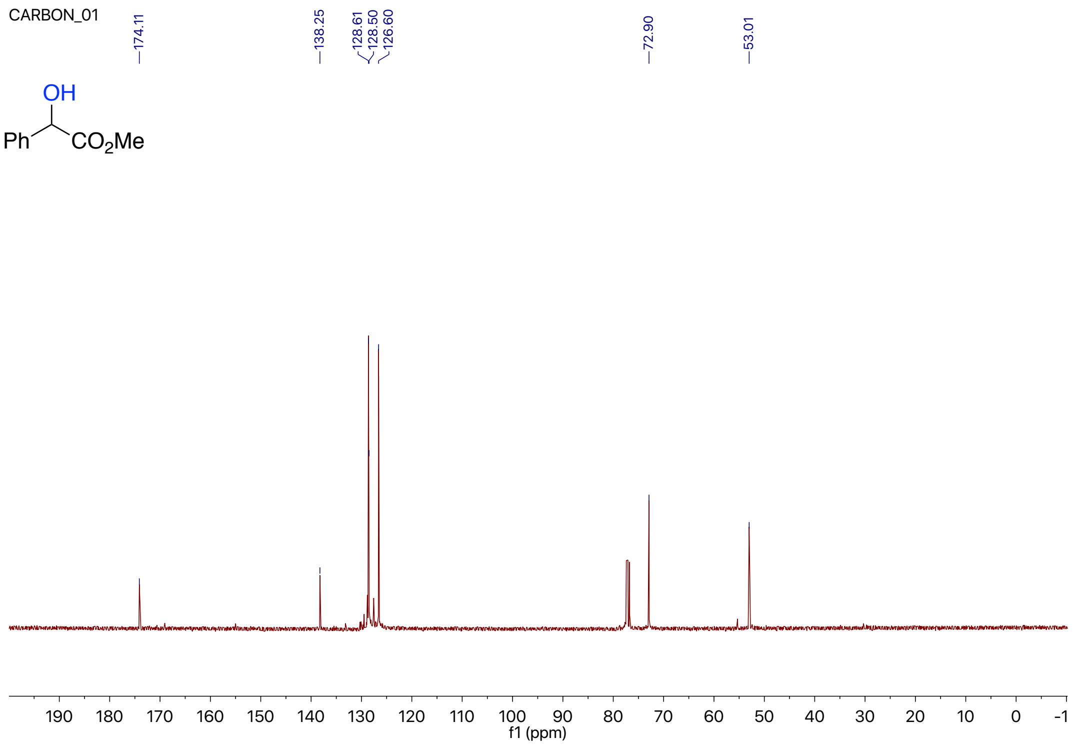

Methyl 2-methoxy-2-phenylacetate (3b)

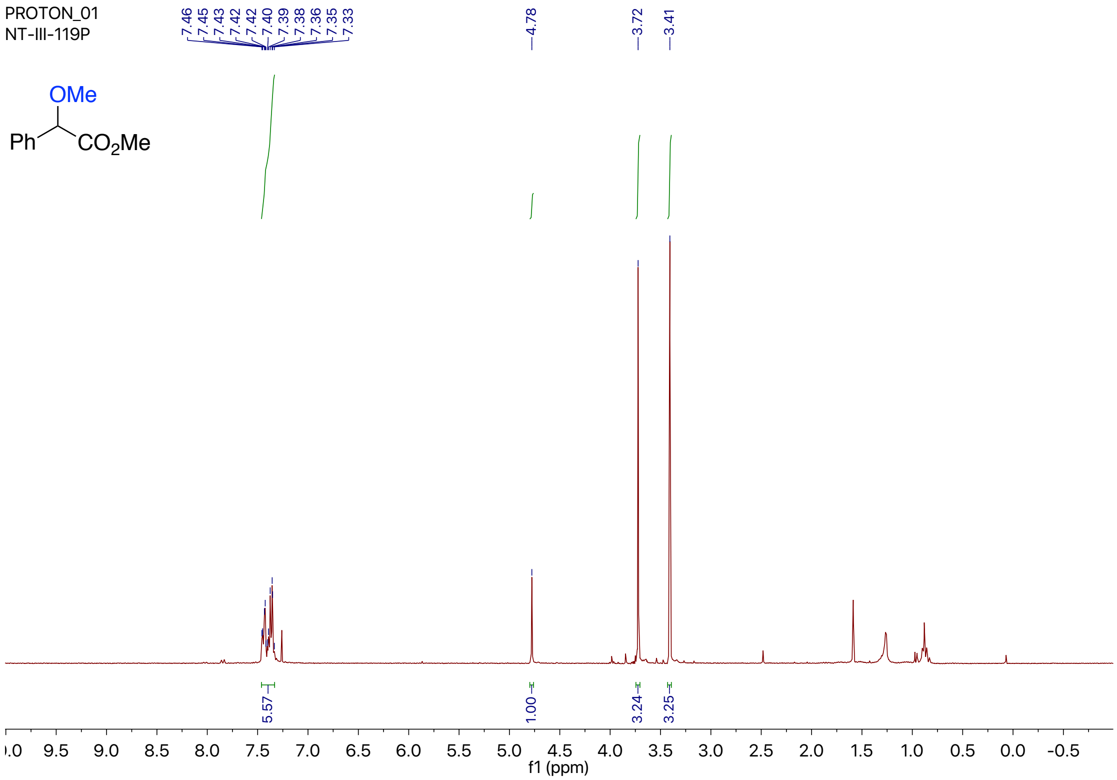

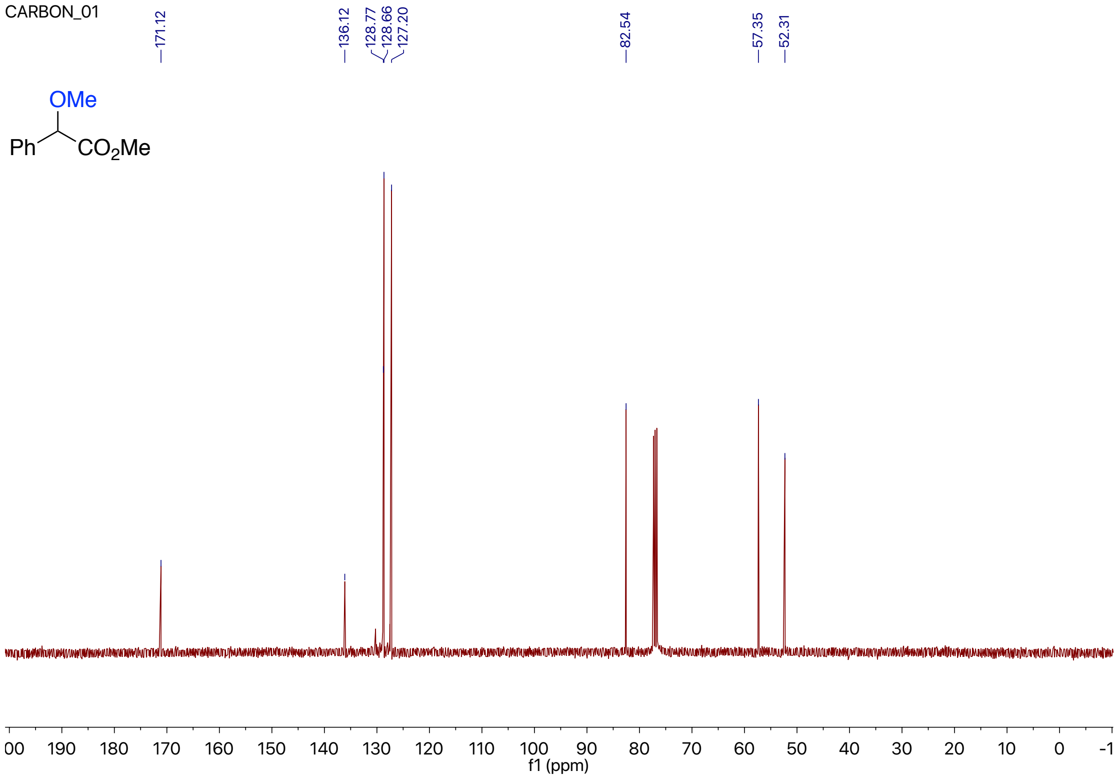

Methyl 2-(allyloxy)-2-phenylacetate (3c)

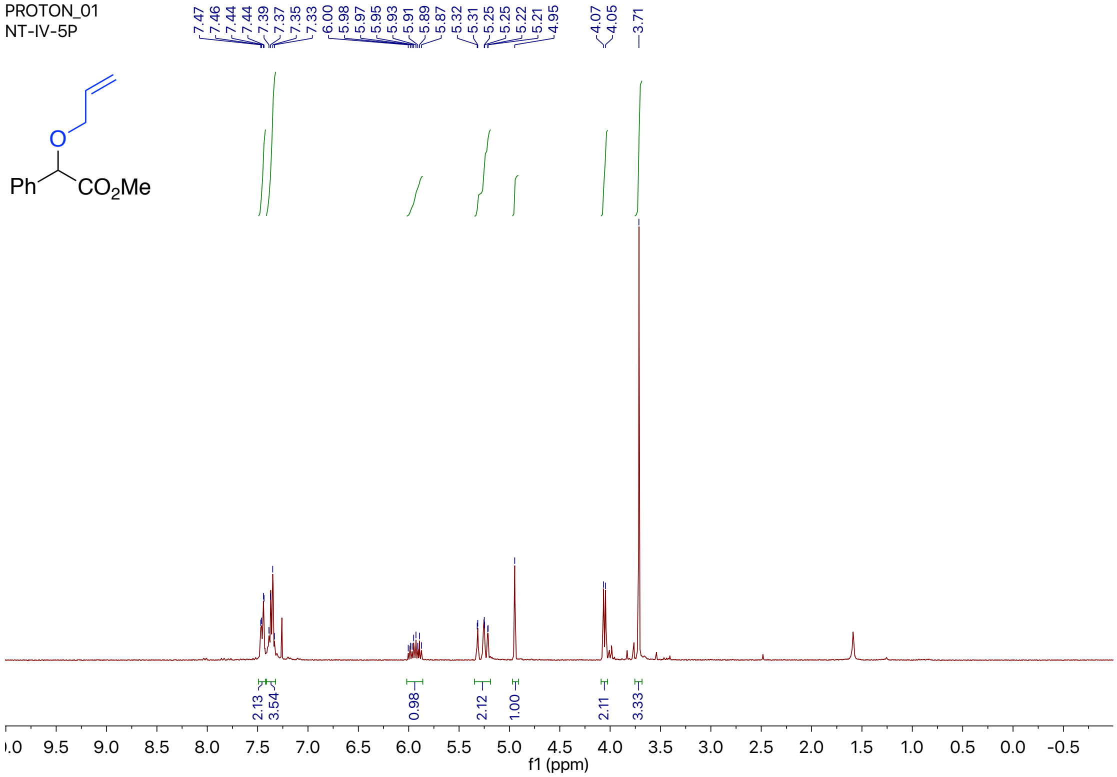

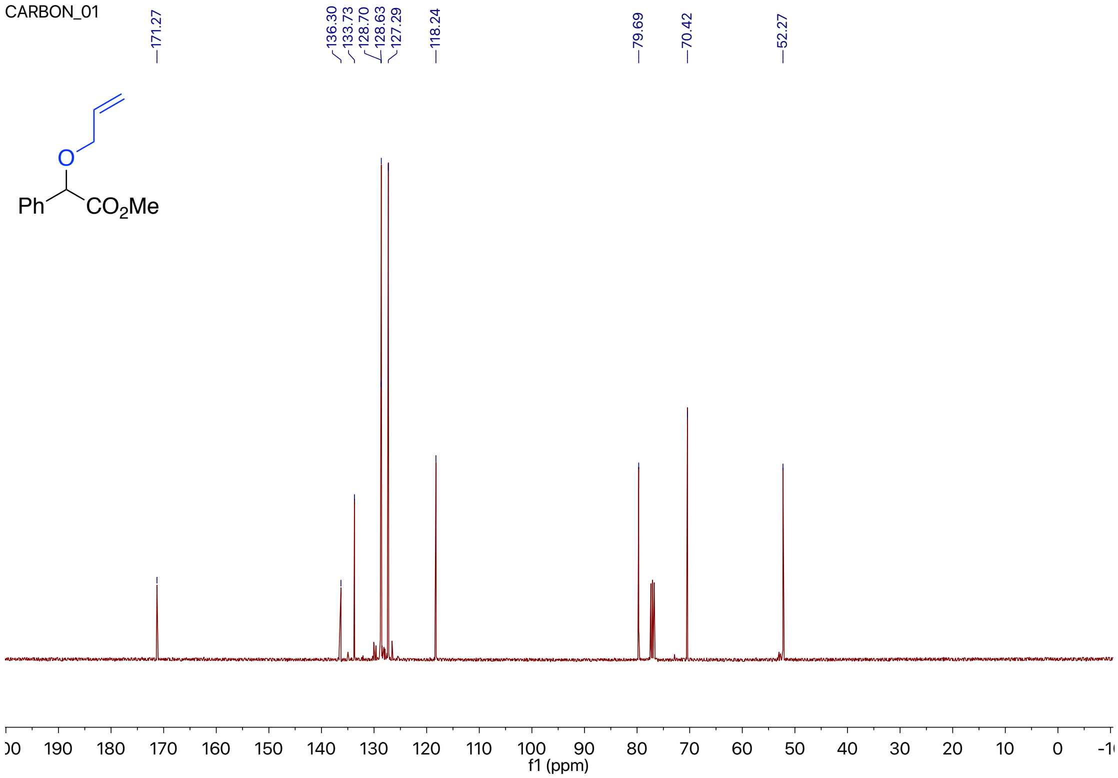

Methyl 2-acetoxy-2-phenylacetate (3d)

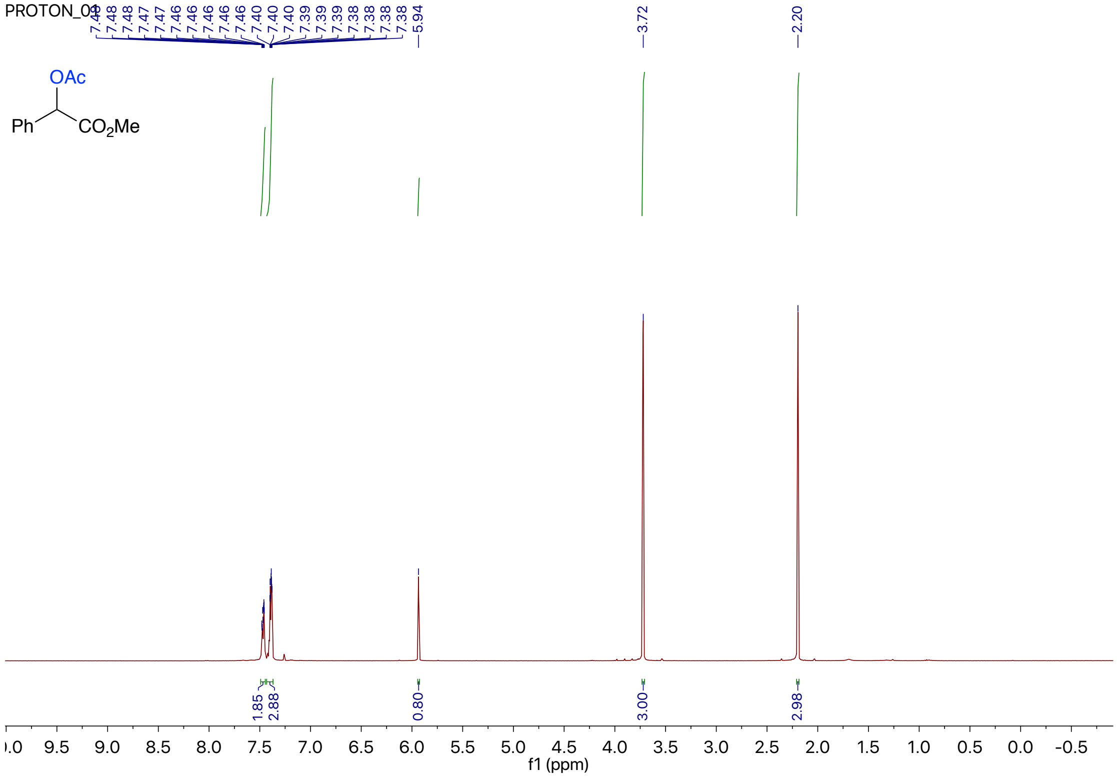

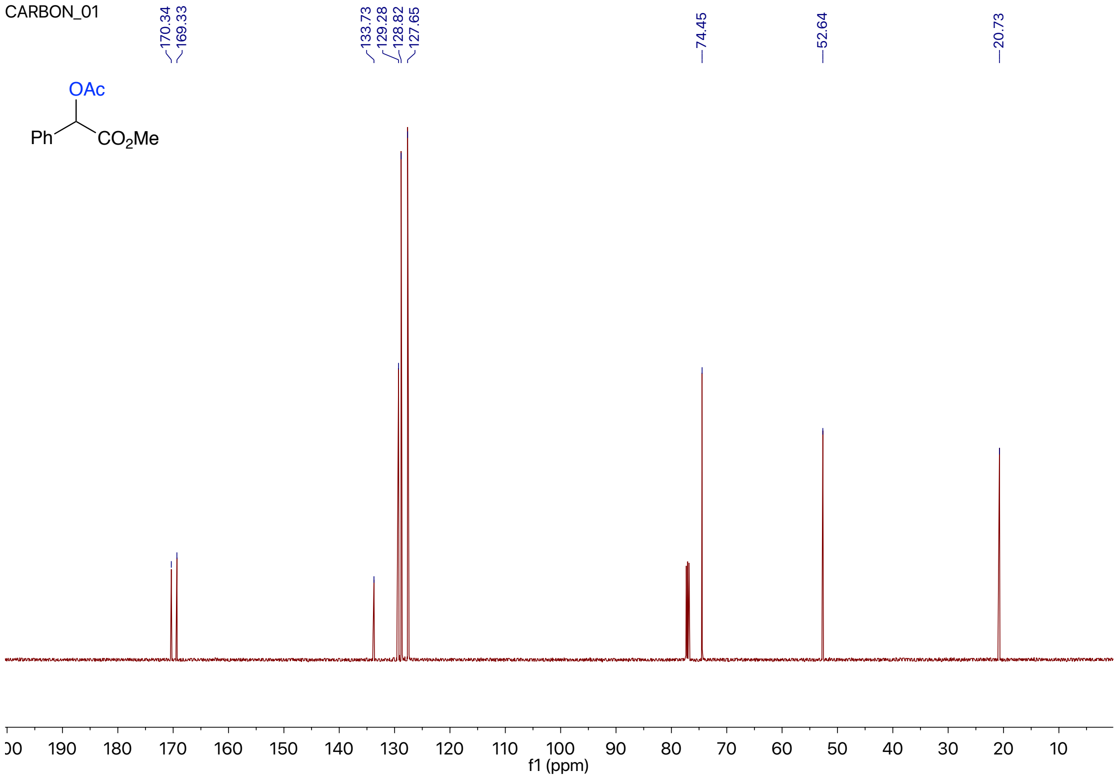

Methyl 2-(di-*n*-butylamino)-2-phenylacetate (3e)

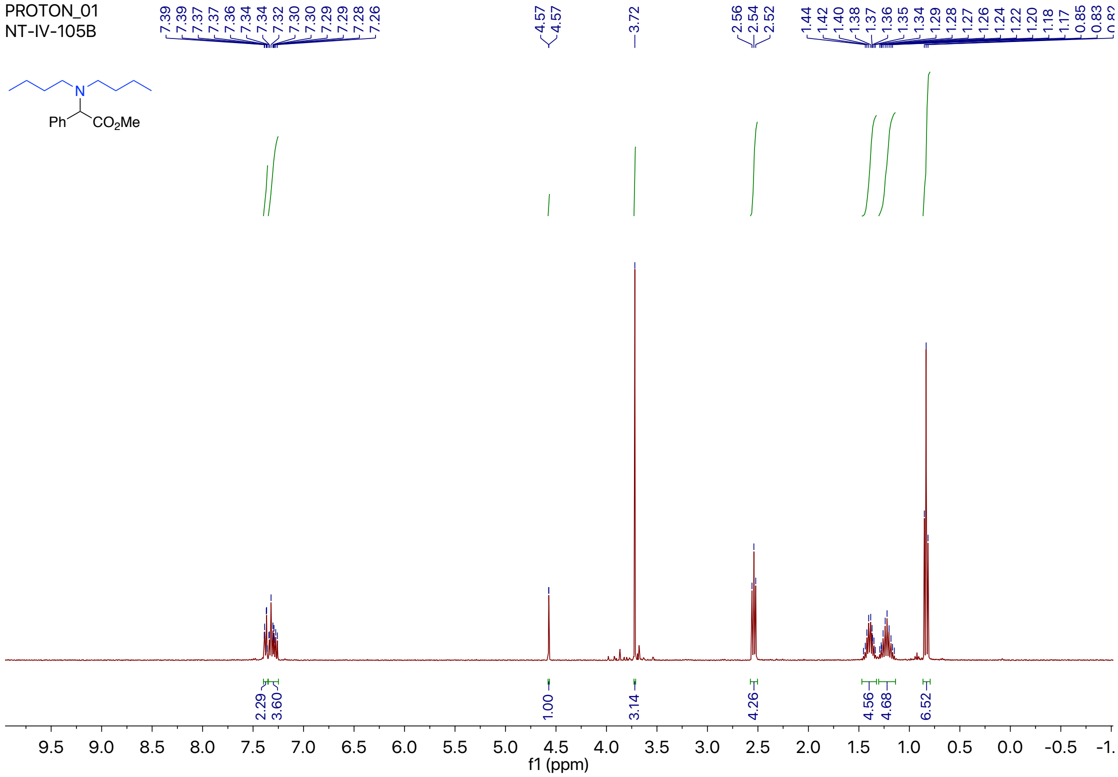

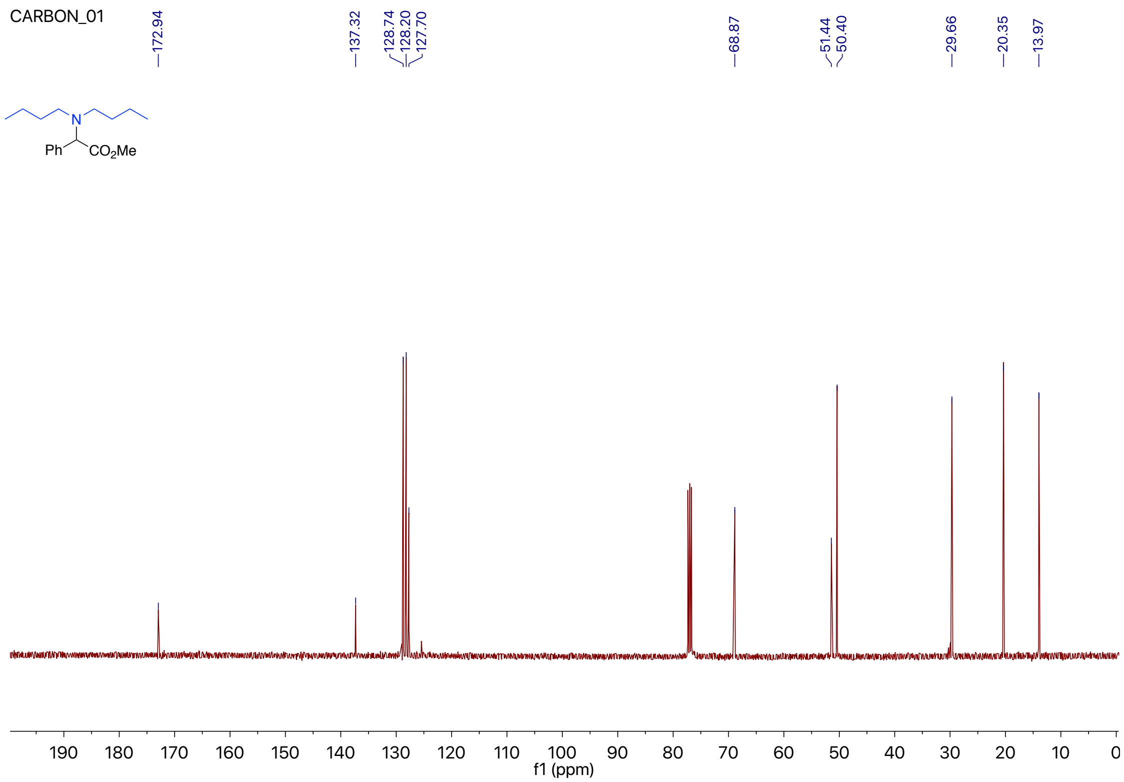

Methyl 2-(methyl(phenyl)amino)-2-phenylacetate (3f)

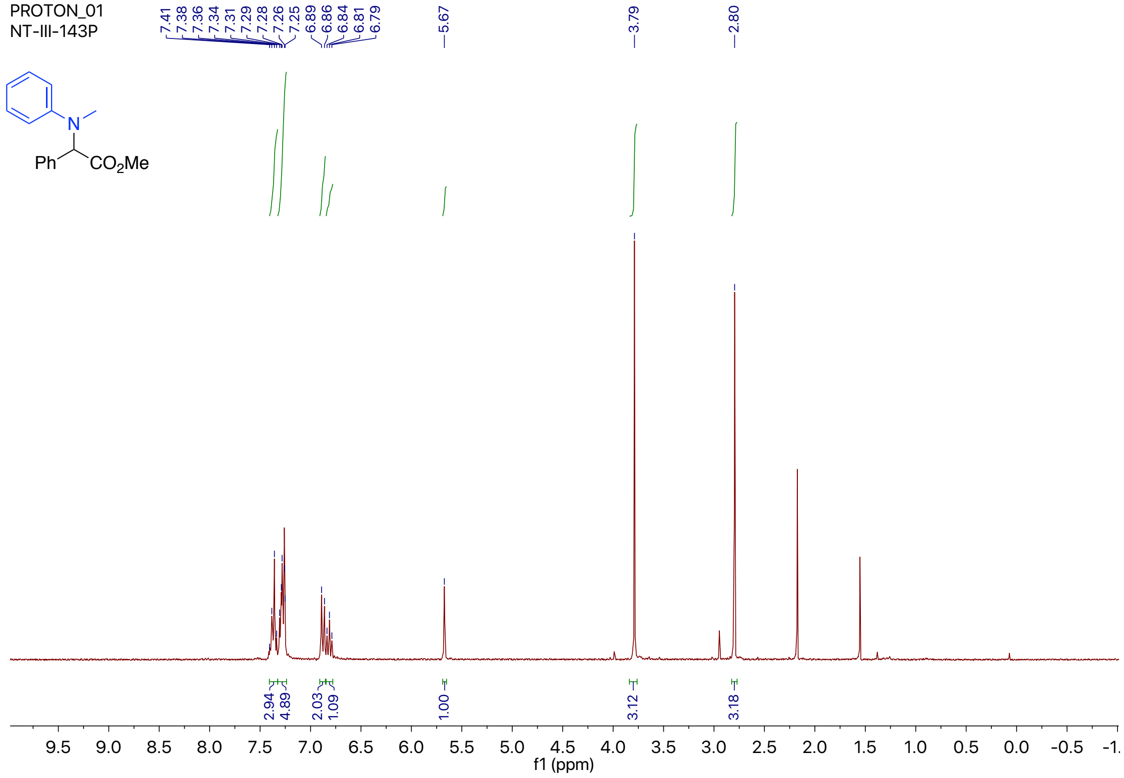

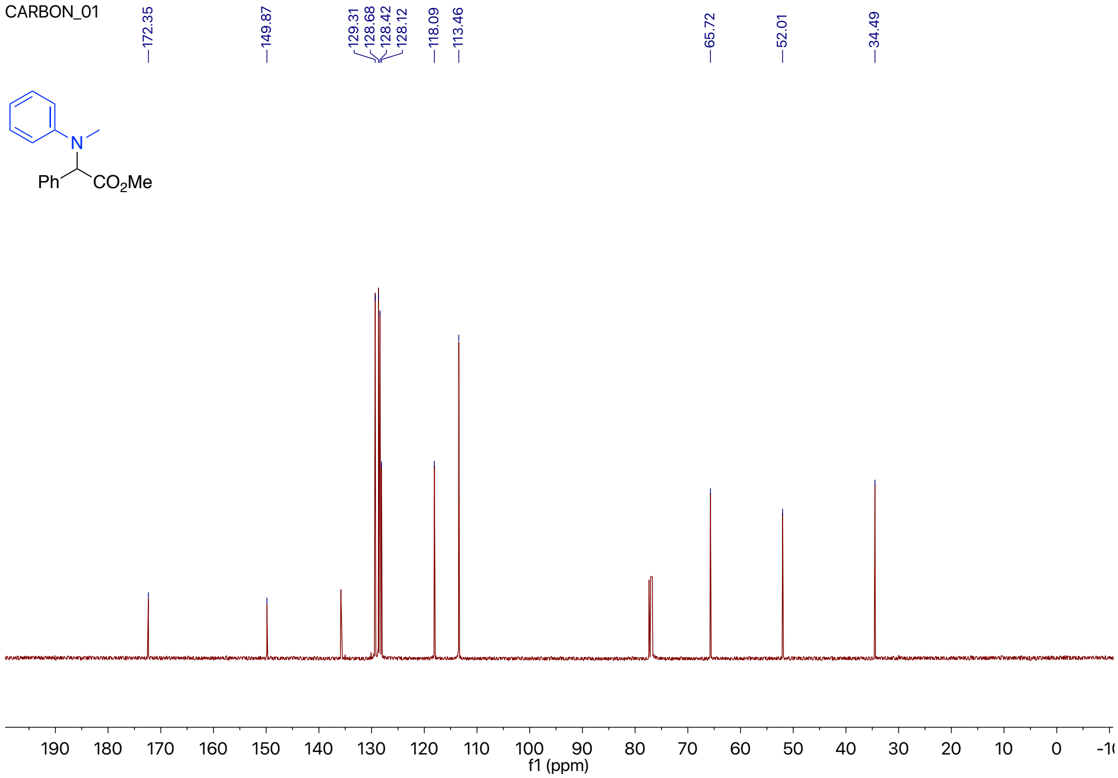

Methyl 2-phenyl-2-(phenylamino)acetate (3g)

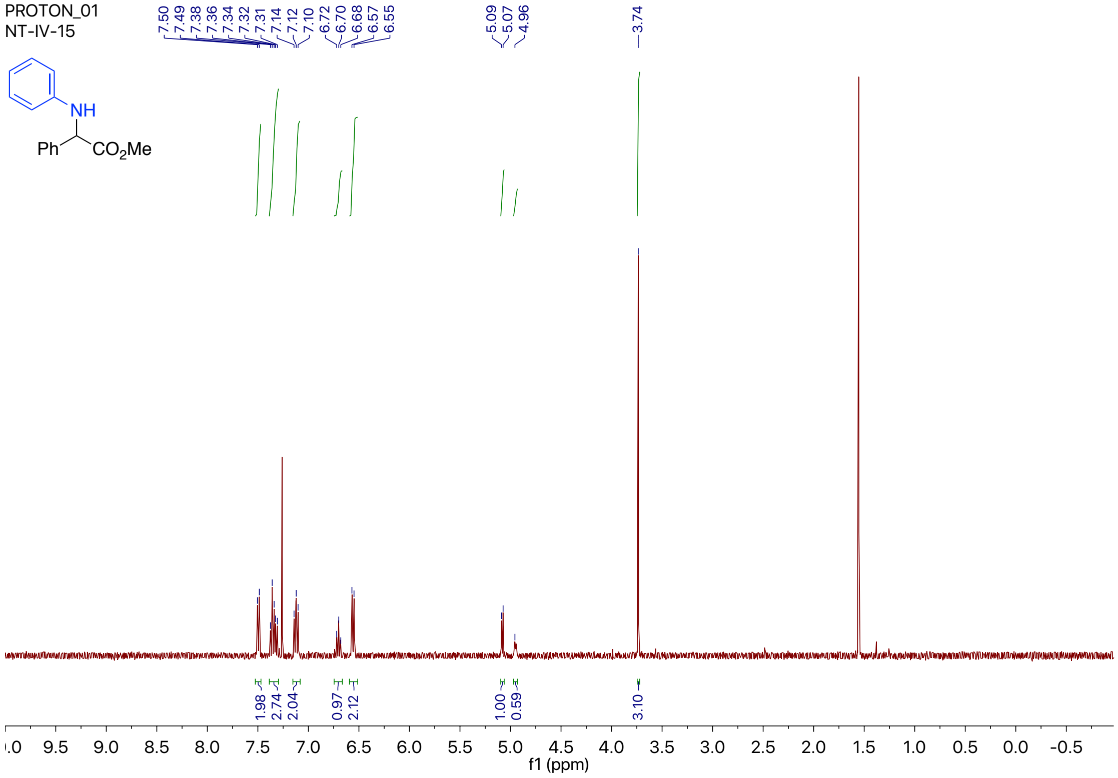

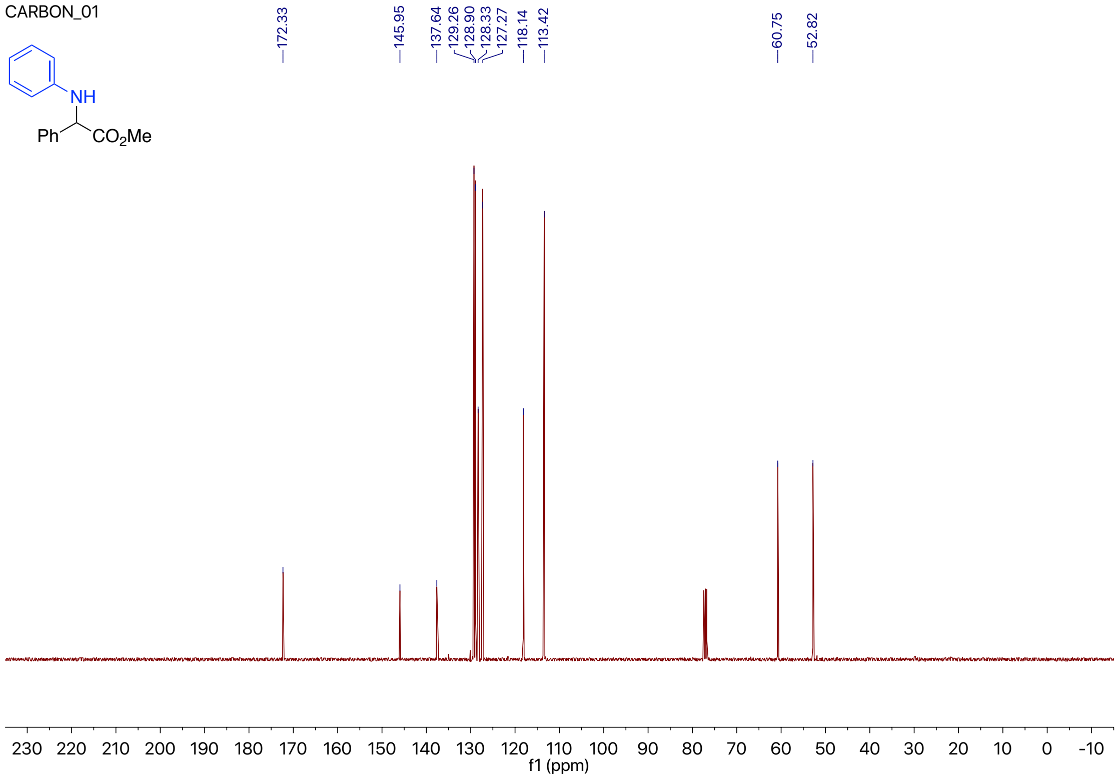

Methyl 2-((2-methoxyphenyl)amino)-2-phenylacetate (3h)

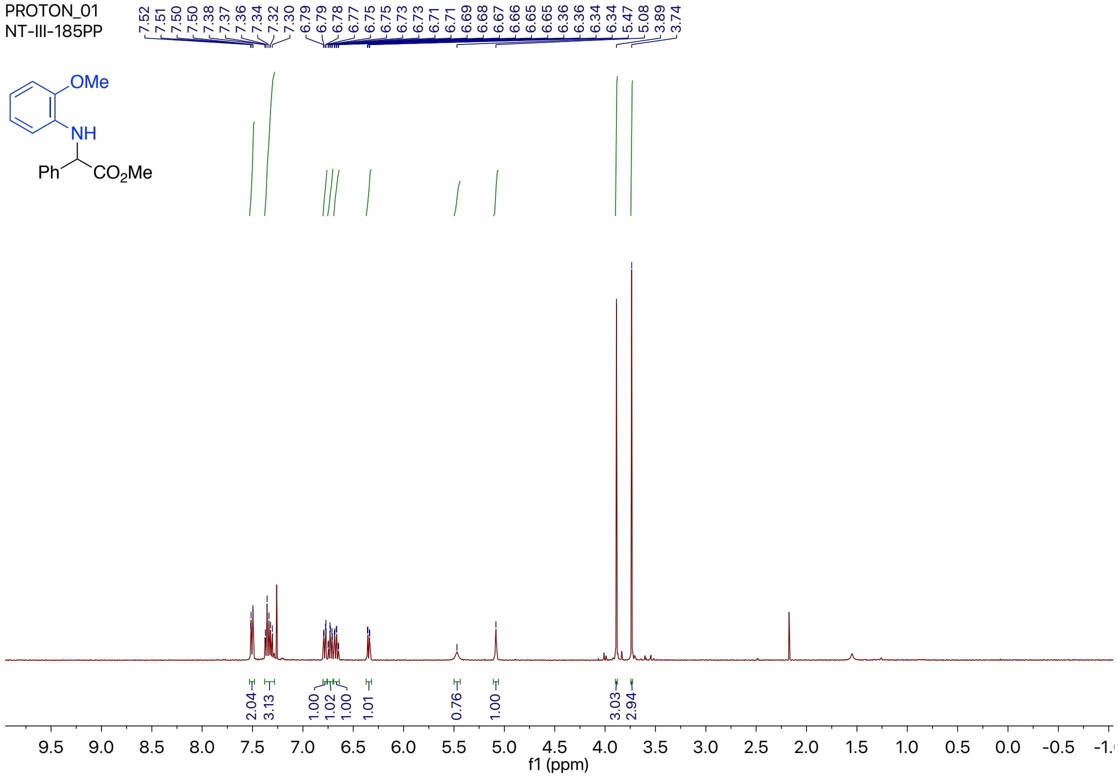

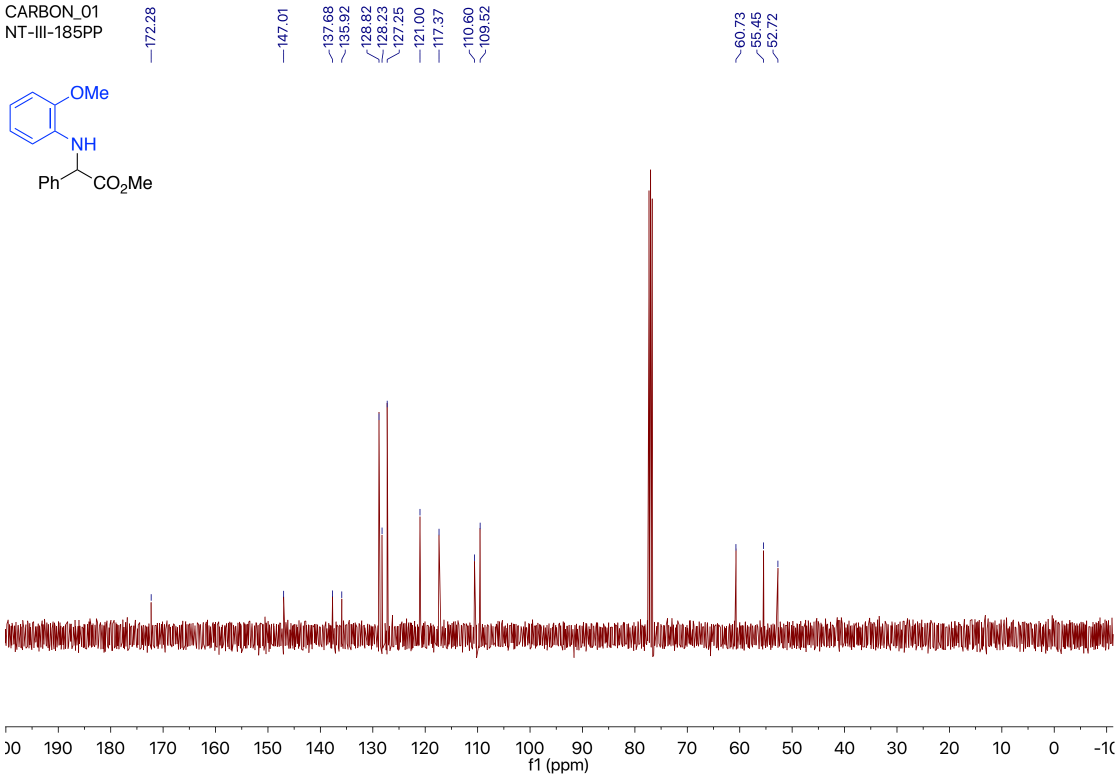

Methyl 2-((3-methoxyphenyl)amino)-2-phenylacetate (3i)

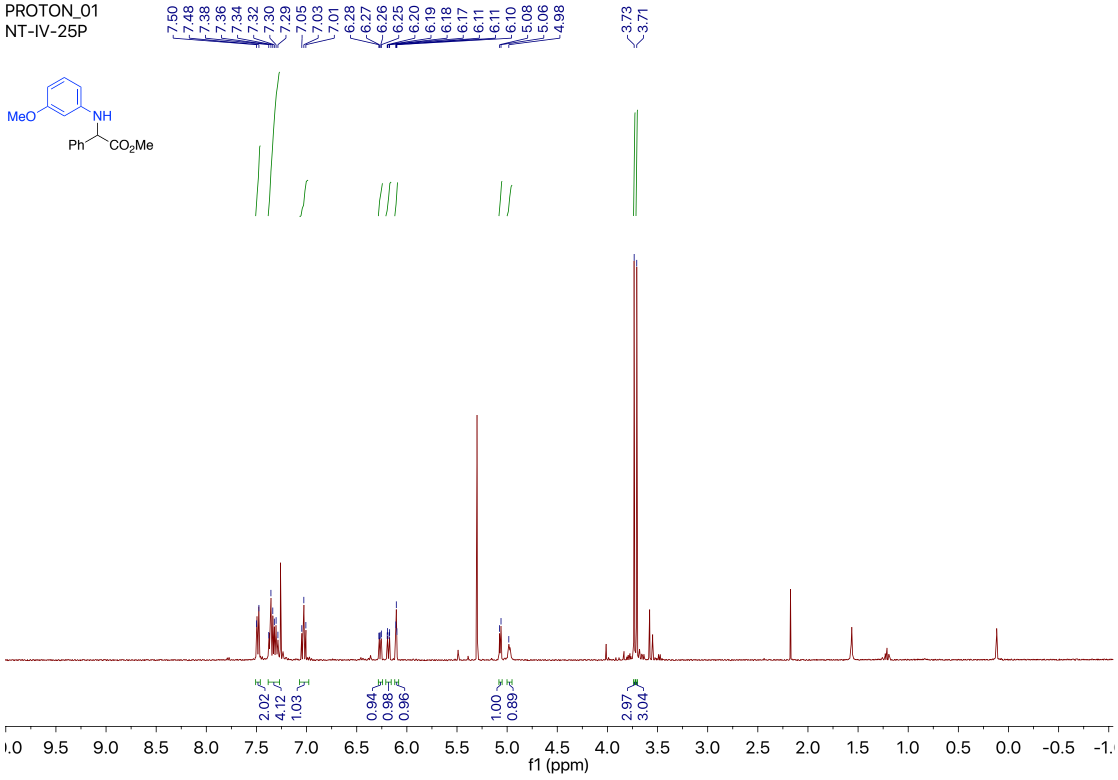

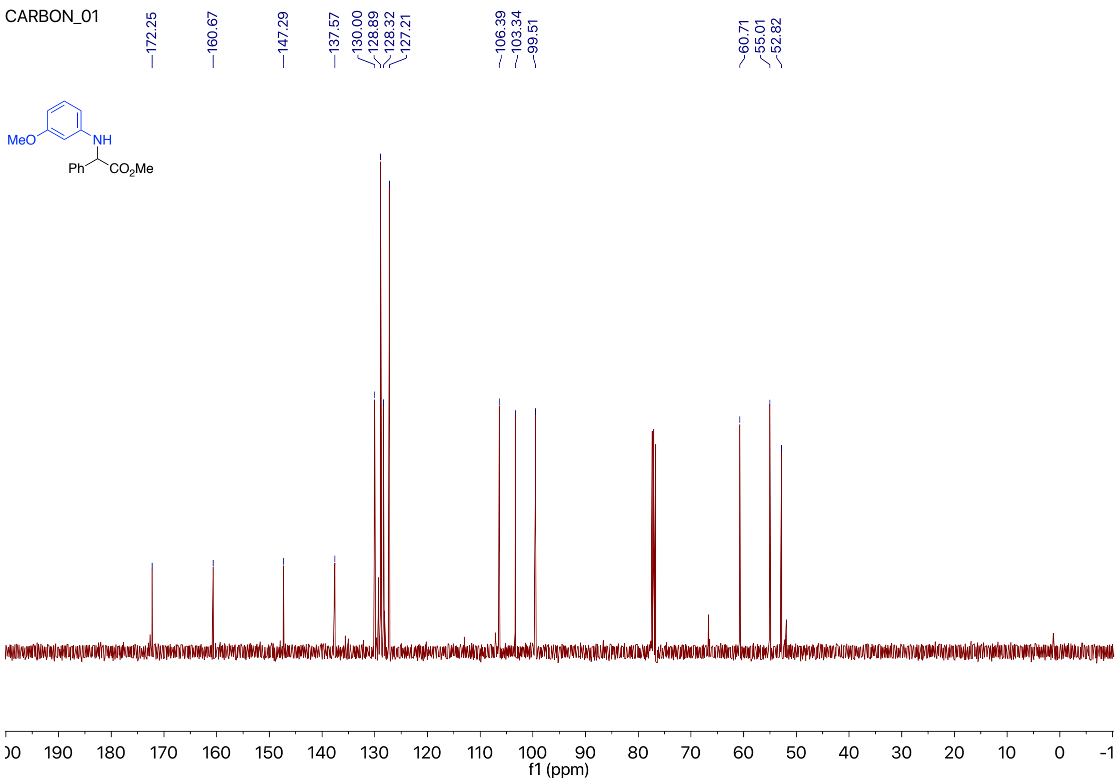

Methyl 2-phenyl-2-(phenylthio)acetate (3j)

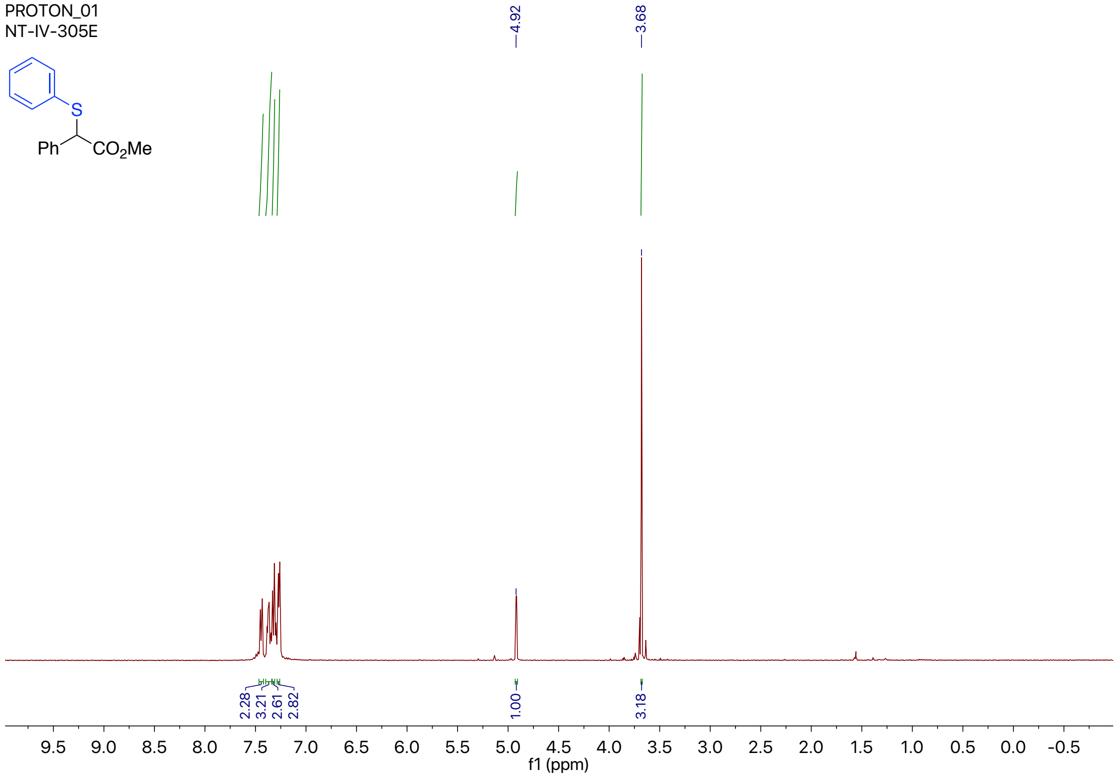

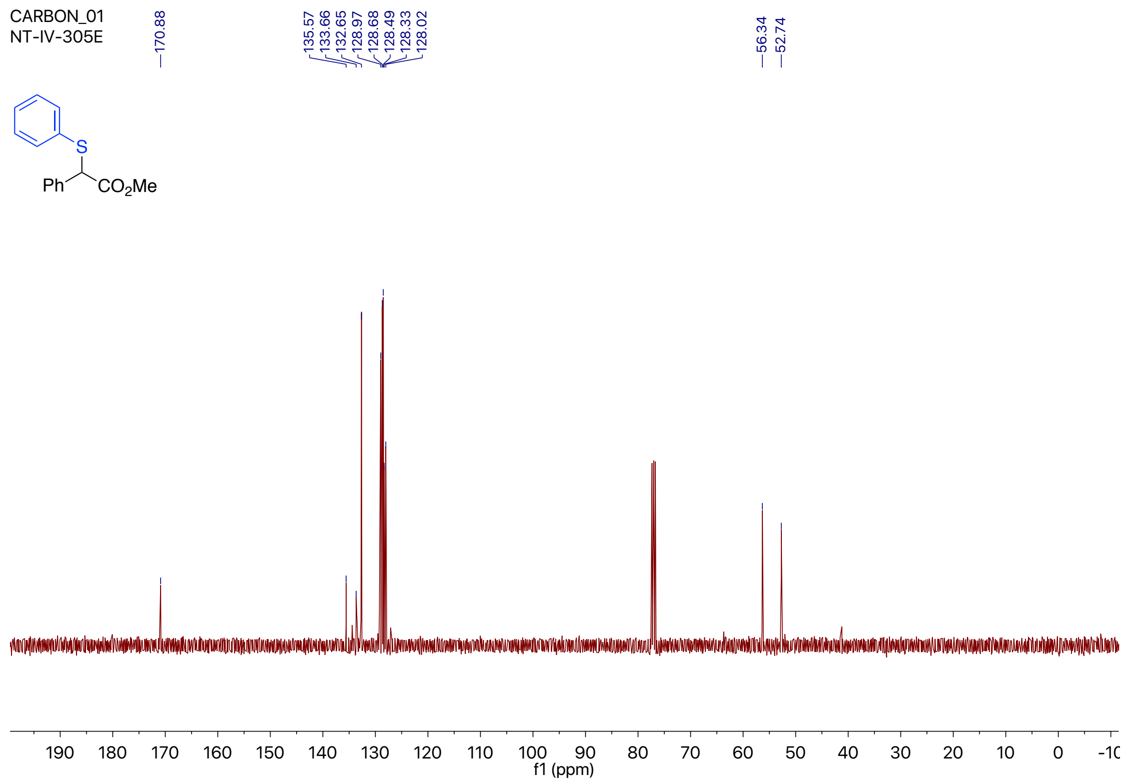

Methyl 2-((4-methoxyphenyl)thio)-2-phenylacetate (3k)

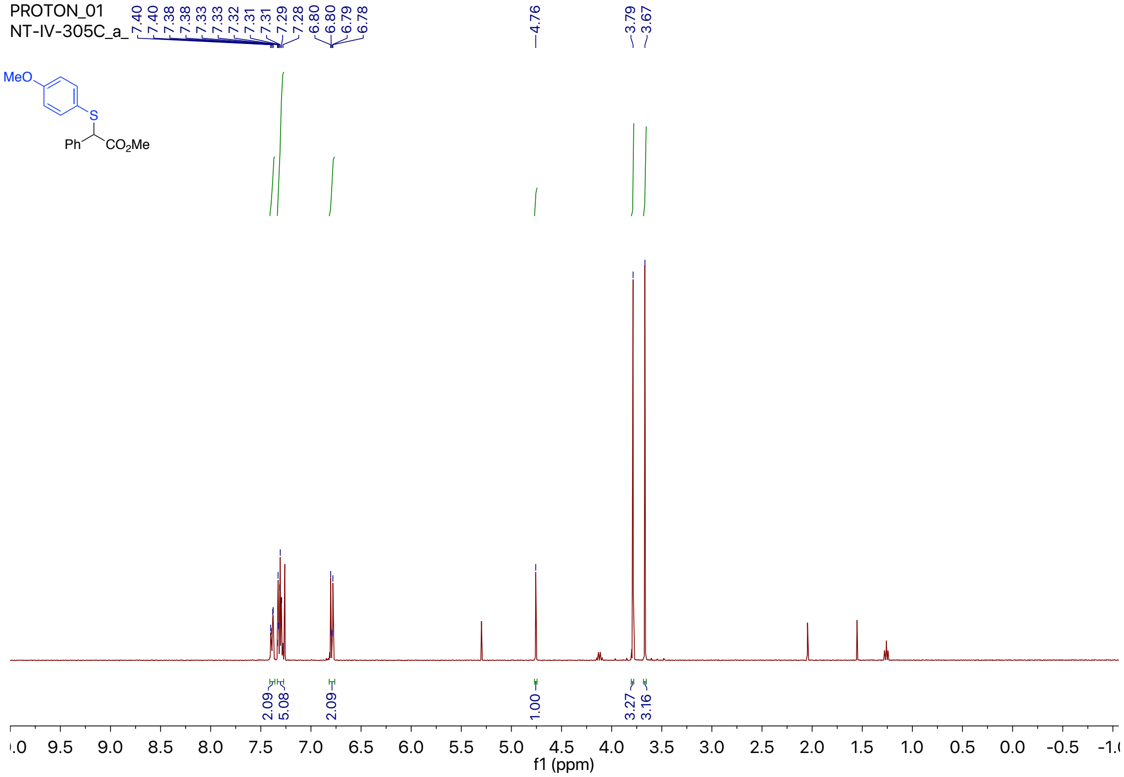

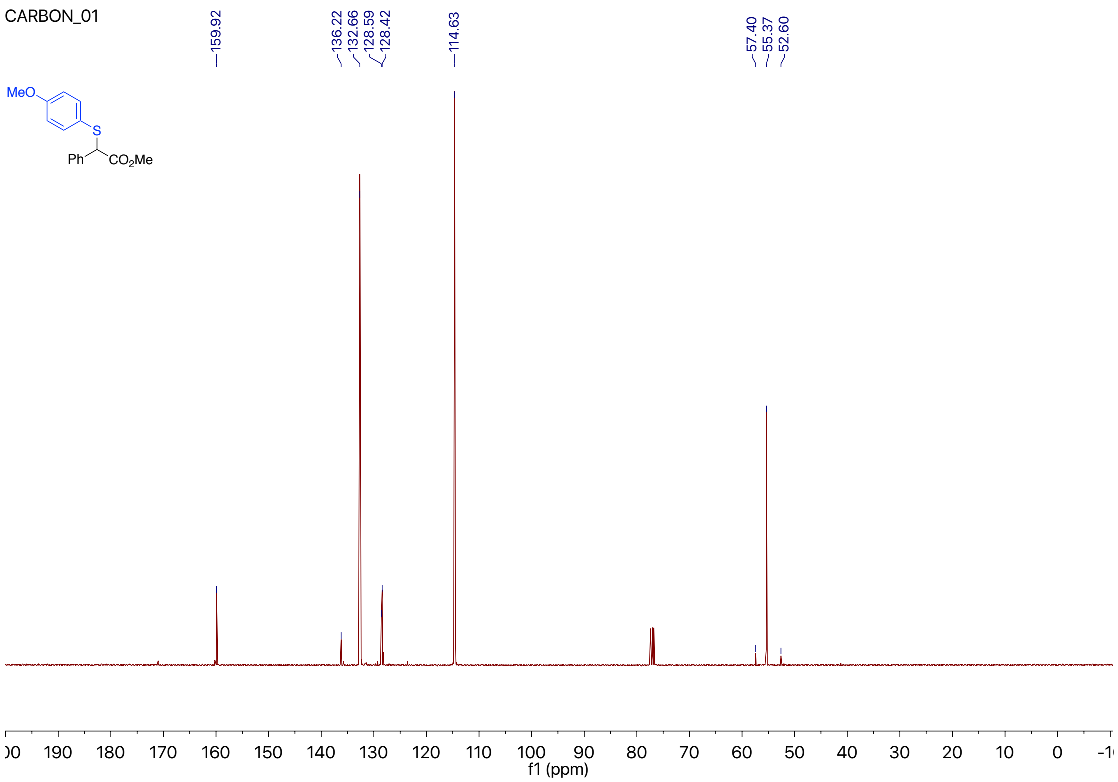

Methyl 2-((4-bromophenyl)thio)-2-phenylacetate (3l)

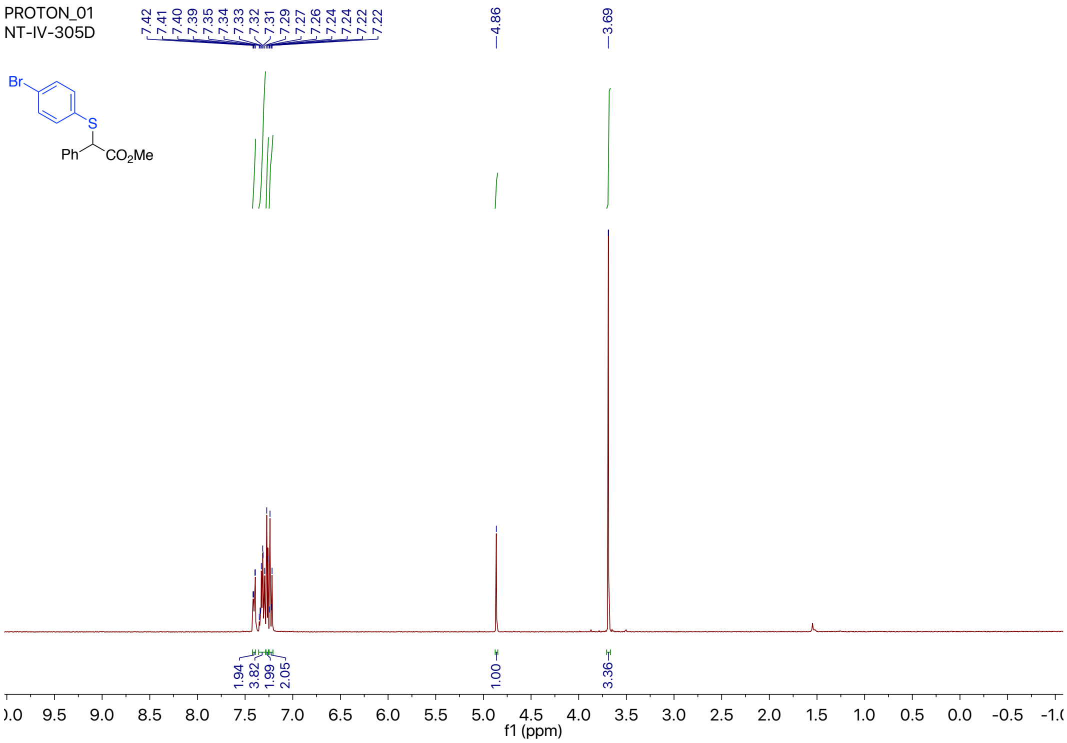

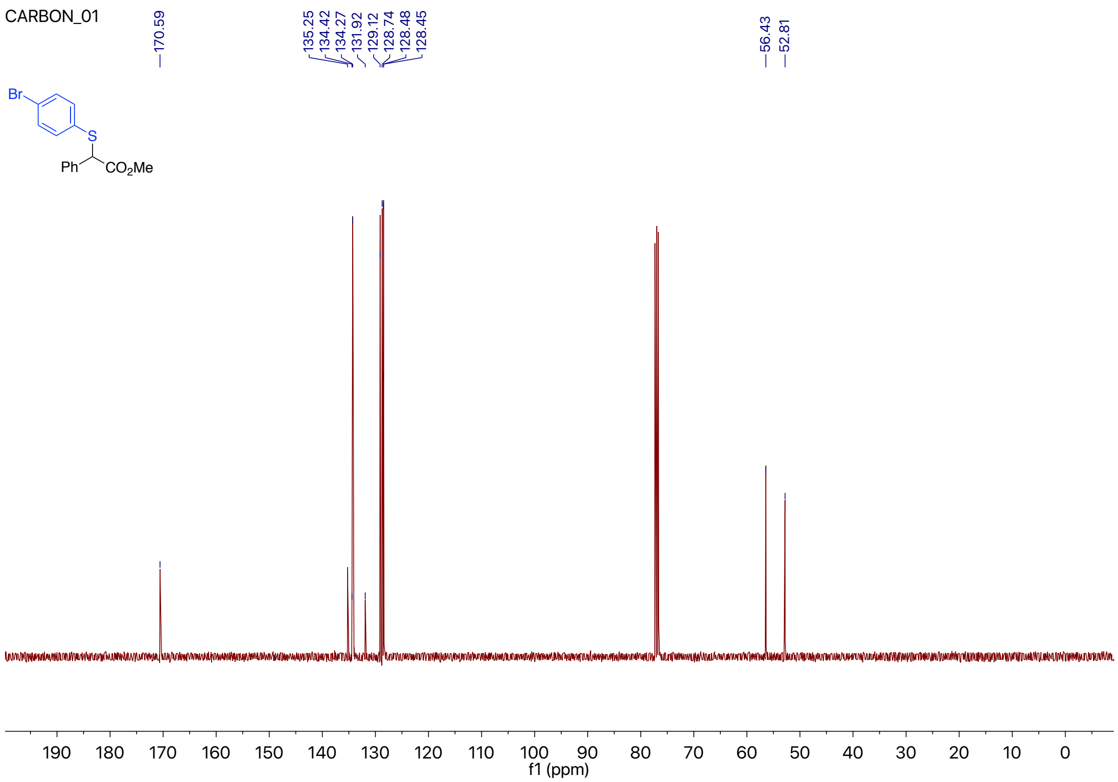

Methyl 2-(benzylthio)-2-phenylacetate (3m)

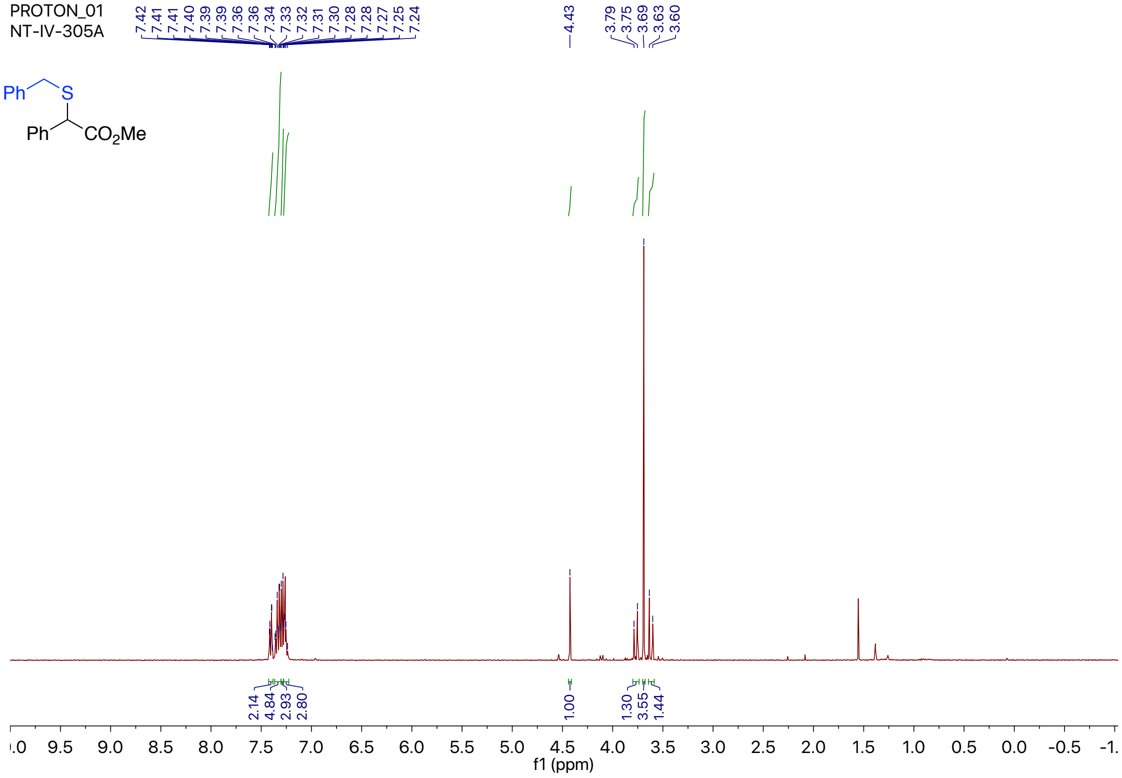

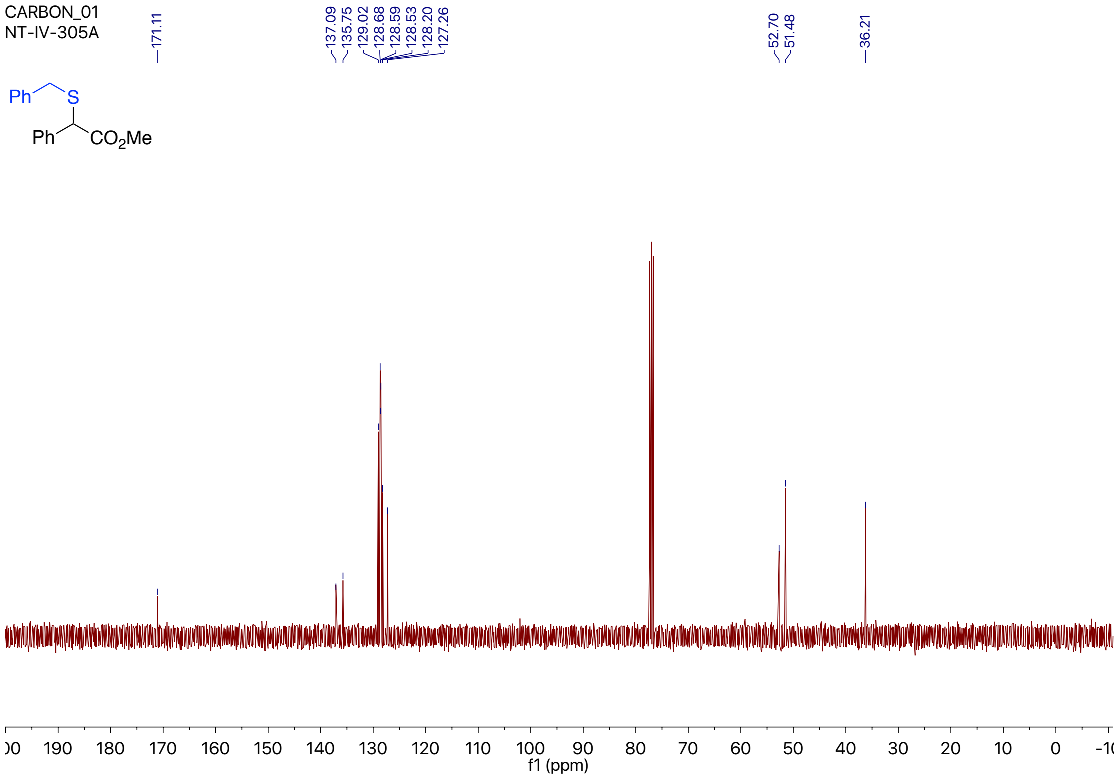
CARBON_01
NT-IV-305A
Ph
S
Ph
CO2Me
171.11
137.09
135.75
129.02
128.68
128.59
128.53
128.20
127.26
52.70
51.48
36.21
f1 (ppm)
